# The effects of social determinants of health on acquired immune deficiency syndrome in a low-income population of Brazil: a retrospective cohort study of 28.3 million individuals

**DOI:** 10.1016/j.lana.2023.100554

**Published:** 2023-07-17

**Authors:** Iracema Lua, Andrea F. Silva, Nathalia S. Guimarães, Laio Magno, Julia Pescarini, Rodrigo V.R. Anderle, Maria Yury Ichihara, Mauricio L. Barreto, Carlos A.S. Teles Santos, Louisa Chenciner, Luis Eugênio Souza, James Macinko, Ines Dourado, Davide Rasella

**Affiliations:** a Institute of Collective Health, Federal University of Bahia (UFBA), Salvador, Bahia, Brazil; b Department of Health, State University of Feira de Santana (UEFS), Feira de Santana, Bahia, Brazil; c Center for Data and Knowledge Integration for Health (CIDACS), Gonçalo Moniz Institute, Oswaldo Cruz Foundation (FIOCRUZ), Salvador, Bahia, Brazil; d Department of Life Sciences, State University of Bahia (UNEB), Salvador, Bahia, Brazil; e Faculty of Epidemiology and Population Health, London School of Hygiene & Tropical Medicine, London, UK; f Nuffield Department of Population Health, University of Oxford, Richard Doll Building, Oxford, UK; g Oxford University Hospitals, Oxford, UK; h Department of Infection and Immunity, St George's University London, London, UK; i Departments of Health Policy and Management and Community Health Sciences, UCLA Fielding School of Public Health, Los Angeles, California, USA; j ISGlobal, Hospital Clinic - Universitat de Barcelona, Barcelona, Spain

**Keywords:** Social determinants of health, Acquired immune deficiency syndrome, Socioeconomic factors, Poverty, Educational attainments, Ethnicity

## Abstract

**Background:**

Social determinants of health (SDH) include factors such as income, education, and race, that could significantly affect the human immunodeficiency virus and acquired immunodeficiency syndrome (HIV/AIDS). Studies on the effects of SDH on HIV/AIDS are limited, and do not yet provide a systematic understanding of how the various SDH act on important indicators of HIV/AIDS progression. We aimed to evaluate the effects of SDH on AIDS morbidity and mortality.

**Methods:**

A retrospective cohort of 28.3 million individuals was evaluated over a 9-year period (from 2007 to 2015). Multivariable Poisson regression, with a hierarchical approach, was used to estimate the effects of SDH—at the individual and familial level—on AIDS incidence, mortality, and case-fatality rates.

**Findings:**

A total of 28,318,532 individuals, representing the low-income Brazilian population, were assessed, who had a mean age of 36.18 (SD: 16.96) years, 52.69% (14,920,049) were female, 57.52% (15,360,569) were pardos, 34.13% (9,113,222) were white/Asian, 7.77% (2,075,977) were black, and 0.58% (154,146) were indigenous. Specific socioeconomic, household, and geographic factors were significantly associated with AIDS-related outcomes. Less wealth was strongly associated with a higher AIDS incidence (rate ratios—RR: 1.55; 95% confidence interval—CI: 1.43–1.68) and mortality (RR: 1.99; 95% CI: 1.70–2.34). Lower educational attainment was also greatly associated with higher AIDS incidence (RR: 1.46; 95% CI: 1.26–1.68), mortality (RR: 2.76; 95% CI: 1.99–3.82) and case-fatality rates (RR: 2.30; 95% CI: 1.31–4.01). Being black was associated with a higher AIDS incidence (RR: 1.53; 95% CI: 1.45–1.61), mortality (RR: 1.69; 95% CI: 1.57–1.83) and case-fatality rates (RR: 1.16; 95% CI: 1.03–1.32). Overall, also considering the other SDH, individuals experiencing greater levels of socioeconomic deprivation were, by far, more likely to acquire AIDS, and to die from it.

**Interpretation:**

In the population studied, SDH related to poverty and social vulnerability are strongly associated with a higher burden of HIV/AIDS, most notably less wealth, illiteracy, and being black. In the absence of relevant social protection policies, the current worldwide increase in poverty and inequalities—due to the consequences of the COVID-19 pandemic, and the effects of war in the Ukraine—could reverse progress made in the fight against HIV/AIDS in low- and middle-income countries (LMIC).

**Funding:**

10.13039/100000060National Institute of Allergy and Infectious Diseases (NAIDS), 10.13039/100000002National Institutes of Health (NIH), US Grant Number: 1R01AI152938.

**Research in context**

**Evidence before this study**

Various definitions of social determinants of health (SDH) express the idea that the living and working conditions of population groups are related to and influence their health, and that socioeconomic inequalities may lead to health inequalities. To investigate studies that evaluated possible associations between SDH and outcomes related to HIV infection/AIDS, we used the Medline and Embase electronic databases through keywords: 'social determinants of health' AND ('human immunodeficiency virus' OR 'acquired immune deficiency syndrome'), with the addition of filters for study type AND ('case control study' OR 'cohort analysis' OR 'comparative study' OR 'cross sectional study' OR 'longitudinal study' OR 'multicenter study' OR 'observational study' OR 'retrospective study'), and publication type ('article' OR 'article in press'). The search was performed without any language restrictions, until December 20, 2022. Additional searches were then made from the references found in the identified articles.

We found 145 searches and identified 15 studies that, to a certain extent, observed that geographic, socioeconomic, and individual level factors were associated with HIV/AIDS outcomes. However, the results were limited to specific populations, used only cross-sectional or aggregate data, had a relatively small sample size, and findings were often inconsistent.

While different HIV/AIDS outcomes and SDH were assessed in these studies, no study has been conducted to evaluate the effects of a wide range of SDH on several AIDS-related outcomes (number of new AIDS cases, AIDS mortality, and case-fatality rates) in a low- and middle-income country (LMIC), such as Brazil, using large cohort data and robust analytical methods.

**Added value of this study**

Our findings show the relevant role of SDH associated with socioeconomic vulnerability in all of the outcomes assessed. Our study provided a unique opportunity to use the largest single, longitudinal dataset of socioeconomic and health data existing in any LMIC, to assess the effects of SDH on AIDS-related outcomes (incidence, mortality, and case-fatality rates) in a LMIC. To the best of our knowledge, this is the first nationwide, longitudinal study, with an large individual sample to show the effect of SDH on AIDS-related outcomes, with strong and consistent findings. They were all influenced by SDH, with an emphasis on geographic (region and area of residence), socioeconomic (income, education, and race/skin color), and living condition (home infrastructure) factors.

**Implications of all the available evidence**

Our findings show that the most economically disadvantaged groups of the population—the poor, black people, the illiterate, and those with inadequate housing—are at greater risk of becoming sick, and dying from AIDS. Understanding the influence of SDH on the health/disease process of AIDS is urgent, to tackle the disease, especially in countries characterized by large economic and health disparities, such as Brazil. Strategies aimed at health education, prevention of infection, timely diagnosis, early initiation of antiretroviral therapy (ART), and adherence to treatment, are crucial to tackle the disease, and are already applied worldwide. However, direct investments in the health sector should be coupled with those to improve social inequalities, focusing on socioeconomic determinants.

## Introduction

The human immunodeficiency virus (HIV) and acquired immunodeficiency syndrome (AIDS) are responsible for a decades-long epidemic, resulting in more than 40 million deaths.^1^ Among its driving factors, the social determinants of health (SDH), understood as the social conditions in which people grow, live and work,^2^ have the potential to influence the risk of acquiring HIV, when, and whether HIV testing is offered, the time of diagnosis, the ability to access healthcare services during and after diagnosis, adherence to ART, the risk of AIDS-related morbidity and mortality, and the risk of onward HIV transmission.

The worst AIDS-related outcomes are still tied to social inequalities, which impede progress towards ending the HIV/AIDS epidemic.^3^ Understanding these structural relationships is essential, to create policies and services that address social inequalities as the root of the problem. While a number of studies have assessed the associations between SDH and HIV/AIDS-related outcomes, including geographic,^4^^,^^5^ socioeconomic,^6^, ^7^, ^8^, ^9^, ^10^, ^11^ household-related,^12^ and demographic-related factors,^6^^,^^8^, ^9^, ^10^, ^11^^,^^13^ several of these present inconsistent findings (i.e., the wealthier are more exposed,^4^ high AIDS rates in municipalities with better living conditions,^12^ or that socioeconomic factors had no significant effect^13^), presumably due to the use of cross-sectional surveys,^7^^,^^8^ aggregate-level data,^7^^,^^11^^,^^12^ small study samples,^8^ or very specific subpopulations.^5^^,^^7^, ^8^, ^9^^,^^13^ However, no study has been conducted to answer the effect of a wide range of SDH on key AIDS epidemiological indicators (number of new AIDS cases, AIDS mortality, and case-fatality rates) in a low- and middle-income country (LMIC), such as Brazil, using large cohort data and robust analytical methods.

Despite Brazil’s pioneering epidemic response^14^—as the first middle-income country (MIC) to offer free ART to all people living with HIV/AIDS (PLWHA), and widespread, free HIV testing—in 2020 the mortality rate in the country was 6 per 100,000 inhabitants.^15^ Of the new infections in Latin America recorded in 2020, almost half (48%) occurred in Brazil.^15^ Brazil is also one of the most unequal countries in the world, and SDH has been shown to strongly influence the distribution of several infectious diseases among its population.^16^

Our study aims to evaluate the effects of SDH on AIDS-related outcomes, namely incidence, mortality, and case-fatality rates, using a large cohort of individuals over a long study period, in Brazil.

## Methods

This retrospective cohort study was based on de-identified, linked, registry data of 28.3 million Brazilians, from 2007 to 2015. The population studied was identified by selecting individuals from the 100 Million Brazilians Cohort,^17^ created by linking data from national social protection programs and governmental registries reporting all deaths and diseases, including HIV/AIDS.

### Study population and settings

**Fig. 1:**
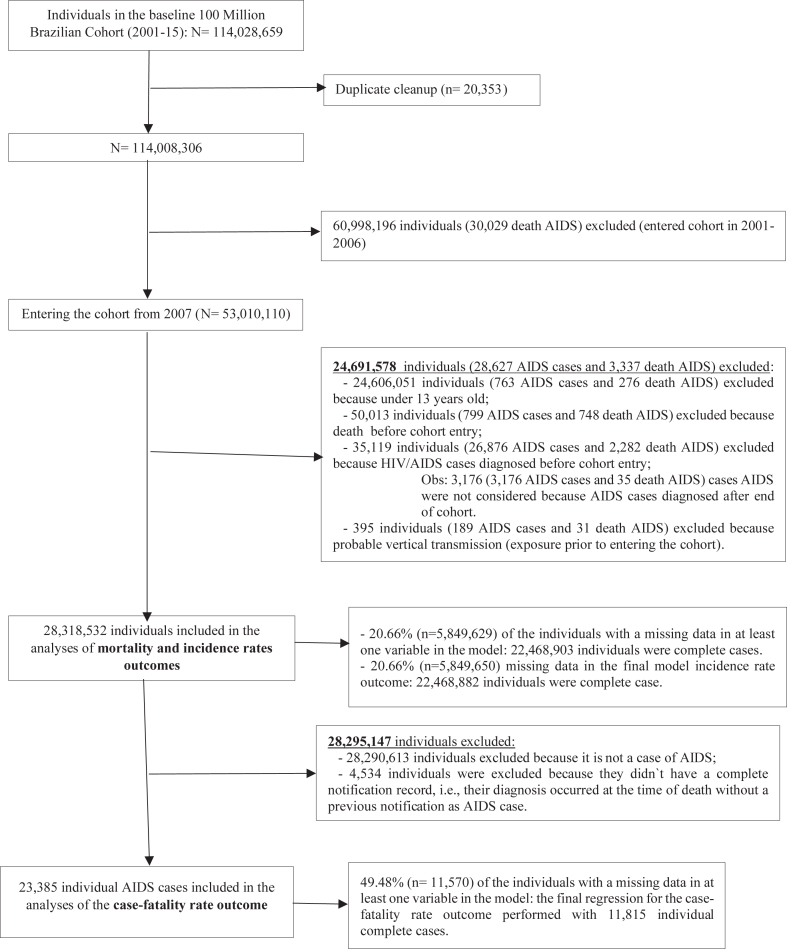
Flowchart of the construction of the study cohort (Brazil, 2007–2015).

The process of selecting individuals into the cohort is shown in Fig. 1. We included all individuals ≥13 years who were enrolled in the cohort during the study period. In Brazil, the epidemiological investigation of HIV/AIDS differs for adults and children, with the age cut-off set at 13 years old. In this study, we focused on adults, to evaluate infections arising from individual living conditions, excluding vertical transmissions. Individuals who had already been diagnosed with AIDS, or who died of AIDS before January 2007, were also excluded.

This study adheres to Brazilian research regulations, and its protocol^18^ was approved by the Collective Health Institute Research Ethics Committee at the Federal University of Bahia (ISC/UFBA), number 41691315.0.0000.5030 (Opinion nº: 3.783.920).

### Data sources and dataset linkages

The study cohort comprises linked data from individuals listed in the Unified Registry of Brazilians (*CadÚnico*), used to identify people eligible for social protection programs in Brazil; data on all AIDS diagnoses recorded in the Brazilian Ministry of Health Information System for Notifiable Diseases (*SINAN*); and data on mortality recorded in the Mortality Information System (*SIM*), from 2007 to 2015.

Details on construction of the 100 Million Brazilians Cohort, from which our study cohort originates, and its extensively evaluated and validated linkage procedures, have been described in previous publications,^17^ and the appendix (p. 2). We emphasize that this cohort is partially representative of the Brazilian population, although it comprises approximately 56% of its entire population. It only includes individuals who apply for social welfare programs, or benefits, who usually have lower incomes, and may not be representative of the entire population. However, this created a unique opportunity to evaluate the effects of SDH on populations excluded from previous HIV/AIDS studies.

### Statistical analysis

We used multivariable Poisson regression models—as in similar SDH studies—to estimate crude and adjusted associations for different SDH with AIDS outcomes over the study period.^16^ Poisson regression models are widely used to analyze cohorts,^19^^,^^20^ since they allow inclusion of ‘compensation/exposure’ terms (the time during which an individual is exposed to the risk of a certain outcome), and enable generation of incidence rate ratios, which facilitate the interpretation of the results.^21^ Sensitivity analyses also tested alternative modeling approaches, including logistic, survival, negative binomial, and zero-inflated regression models (Supplementary Tables S1–S3 in the appendix, pp. 7–12), to ensure the robustness of our findings.

Follow-up (person-time) was defined as years from the date of admission to the cohort until the time of AIDS diagnosis (incidence), AIDS-related death (mortality and case-fatality rates), death from other causes, or end of the study timeframe. For case-fatality rates, follow-up commenced from the date of AIDS diagnosis.

**Fig. 2:**
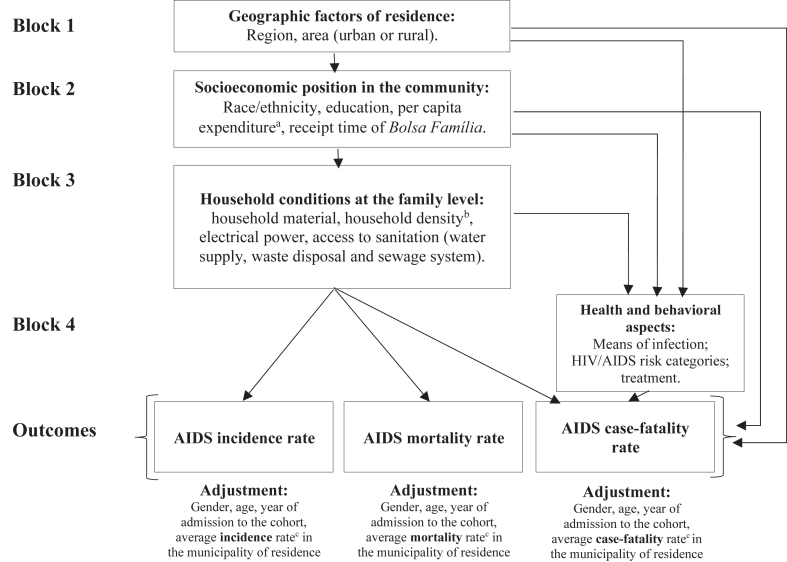
Conceptual model: Hierarchical effect of SDH on AIDS incidence, mortality, and case-fatality rates. ^a^Per capita family expenditure proportional to the national, monthly minimum wage: constructed as a proxy for the wealth level, from the sum of all family expenditures (e.g., water and electrical power, food, cooking gas, rent, medication, transportation, and others), divided by the number of residents, and calculated in proportion to the minimum wage for the year of admission to the cohort. The result was divided into 5 wealth levels, considering those with per capita family expenditures of 1 or more minimum wages as the highest wealth quintile, and those with no declared expenditure as the lowest wealth quintile. ^b^Household density: derived from the number of residents per room in the home. ^c^Average rates calculated from the sum of the annual municipal rates, divided by 9 years (2007–2015). SINAN and SIM data was used, and aggregated at municipal and annual levels in Brazil, from 2007 to 2015.

The analysis used a hierarchical approach^16^ with complete cases, whereby distal, intermediate and proximal determinants were assessed in blocks (Fig. 2). Accordingly, consecutive, adjusted Poisson regression models were estimated using robust standard errors, clustered in the municipality of residence, and separately for each outcome. Thus, each outcome was considered as a primary outcome.

The outcome variables available were i) new AIDS cases, defined by adapted CDC, Rio de Janeiro/Caracas, and death criteria; ii) AIDS-related death as the underlying cause, considering the International statistical classification of diseases and related health problems, 10th revision (ICD-10) codes related to AIDS (B20–B24) (Supplementary Charts S1 and S2 in the appendix, pp. 3–6). Explanatory variables were obtained at the cohort baseline and included in Block 1: geographic factors (region, area of residence); Block 2: socioeconomic factors (educational attainment, race/skin color, wealth level, duration of receipt of government conditional cash transfer programs—*Bolsa Família*—if qualified as a beneficiary); Block 3: household conditions (household building material, access to electrical power, household density, and sanitation conditions—type of water supply, waste disposal, and sewage system); or Block 4: behavioral factors (HIV exposure category, means of HIV transmission, and ART). Age, sex, year of admission into the cohort, and municipal level data on AIDS endemicity and mortality were included as adjustment variables in all analyses (Fig. 2).

We highlight that the ethnicity variable (Block 2) originally had five response categories (white, yellow/Asian, pardo, black, and indigenous). However, the yellow/Asian category represents a very small proportion of the Brazilian population. In our cohort, only 0.47% of individuals self-identified as yellow/Asian, and this segment also represents a small number of cases and deaths from AIDS (Supplementary Table S8 in the appendix, pp. 26–29). Due to the small sample number, with the majority having socioeconomic characteristics similar to those of individuals who self-identify as white in the country, and presenting low AIDS incidence and mortality rates (lower than those of white people, which is the reference group) (Supplementary Table S11 in the appendix, pp. 36–38), we opted to merge these two race/skin color categories (white/Asian), and highlight those who represent the most vulnerable groups: pardo, black, and indigenous people.

Models selections included retaining variables associated with AIDS outcomes in each model, with a p-value of ≤0.10 for the partial models, and 0.05 for the final model, as in previous studies.^16^ For the non-binary categorical variables, we also used overall p-value estimates for all response categories, obtained by Wald hypothesis tests on the model parameters. Model 1 included factors specific to each analysis block (intra-block analyses). After filtering the variables in each specific block, backwards, stepwise regression was performed with hierarchical input from each block (inter-block analyses). Thus, Model 2 includes variables from Blocks 1 and 2. Model 3 includes final variables from Model 2 and Block 3.

For each estimated model, listwise deletion was used to deal with missing data, because analyses comparing characteristics between individuals with complete and incomplete cases for each AIDS outcome (Supplementary Table S10 in the appendix, pp. 32–34) showed that missing data were randomly distributed. As a sensitivity analysis for case-fatality rates, proximal health and behavioral factors (Block 4) have also been included as independent variables in Model 4 (Fig. 2), since they are available in the SINAN dataset (variables not available for incidence and mortality rate analyses). Inclusion of these factors was expected to decrease the observed effects of some distal variables, since they may mediate SDH effects,^6^^,^^22^ and their inclusion may reduce the strength of associations.^23^

Additionally, specific sensitivity analyses were conducted (Supplementary Tables S4–S8 in the appendix, pp. 13–25): a) to verify the robustness of the estimates to potential confounding at aggregate level, including other adjustment variables at the municipal level (namely, primary health care coverage, number of specialized clinics, physicians, and hospital beds per 1000 inhabitants, Gini index, and extreme poverty and unemployment rates); b) to verify the influence of the quality level of surveillance data, only fitting the models for a subgroup of municipalities with high quality vital information; and c) check possible changes in effects over time, either by substantial changes in SDH effects, or alterations to HIV/AIDS health policies.

All analyses were performed using Stata® 15.0.

### Role of the funding source

This study was funded by the National Institute of Allergy and Infectious Diseases—NAIDS/NIH, Grant Number: 1R01AI152938.

The study funders played no role in the study design, data collection, data analysis, data interpretation, or writing of the paper. None of the authors were precluded from accessing study data, and they accepted the responsibility to submit for publication.

## Results

Among 28,318,532 Brazilians included in the cohort (Fig. 1), 27,919 new AIDS cases and 9530 deaths were identified in the follow-up. Since this is a closed cohort, the loss of follow-up in the cohort was due to death (717,202, 2.53%), and the annual loss rates are presented in the supplementary material (Supplementary Figure S1 in the appendix, p. 29).

### Social-demographic characteristics of the participants

For the cohort participants evaluated, they had a mean age of 36.18 (sd: 16.96), the majority were women (14,920,049, 52.69%), young adults (24–34.9 years old) (8,328,072, 29.41%), live in the Northeast (9,309,822, 32.88%) and Southeast (10,413,346, 36.77%) regions, and urban areas (22,688,901, 80.38%). With regards to socioeconomic status (Block 2), the majority had elementary school education (15,216,511, 59%), were pardo (15,360,569, 57.52%), classified as level 5 of wealth (lowest wealth) (7,946,836, 28.07%), and were Bolsa Família Program beneficiaries (18,195,279, 64.75%). They lived in houses with favorable conditions (Block 3), with the majority living in houses constructed with bricks (22,414,010, 81.34%), with electricity (24,490,897, 88.88%), a low domiciliary density (up to 2 residents per room) (24,712,132, 95.50%), a public water supply network (20,968,506, 76.10%), public garbage collection (22,257,375, 80.78%), and a sanitary sewage system (14,073,902, 52.18%) (Supplementary Table S8 in the appendix, pp. 26–28).

For those diagnosed with AIDS in the period, we observe similar proportions in the regions, with more cases in the Northeast (7993, 28.63%) and Southeast (9486, 33.98%). Higher proportions of AIDS cases were observed in men than in the general population (15,085, 54.03%), were young adults (24–34.9 years old) (10,822, 38.76%), resident in urban areas (25,121, 90.96%), had an elementary school education (17,200, 70.32%), were black (3431, 12.98%), and had the lowest level of wealth (11,813, 42.32%). Similarly, AIDS-related deaths were higher among men (5395, 56.61%) and young adults (24–34.9 years old) (3360, 35.26%), in the Southeast (3938, 41.32%), resident in urban areas (8659, 91.56%), had an elementary school education (6293, 73.89%), were black (1347, 14.84%), and had the lowest level of wealth (4143, 43.48%). In both cases, few differences in concentration were observed for household conditions (Block 3) (Supplementary Table S8 in the appendix, pp. 26–28).

The individuals who were no longer followed-up had similar social-demographic characteristics to those observed in the total sample (Supplementary Table S9 in the appendix, pp. 30–32).

### AIDS incidence, mortality, and case-fatality rates in the sample

The AIDS incidence rate was 20.22 per 100,000 person-years (py) (27,900/137,986,981); the AIDS mortality rate was 6.90 per 100,000 py (9528/138,068,520); and the AIDS case-fatality rate was 6.90 per 100 py (4040/58,517.92) (Table 1). There was a trend of increased AIDS incidence and mortality rates, and a downward trend in AIDS case-fatality rates over the study period (Supplementary Figure S2 in the appendix, pp. 39–40).

**Table 1:** Incidence, mortality, and case-fatality rates from AIDS in Brazilians aged 13 years or older (N = 28,318,532) recorded in CadÚnico from 2007 to 2015, stratified by geographic, socioeconomic, household characteristics and health and behavioral aspects.

	AIDS cases in cohort		AIDS death in cohort		AIDS death in PLWA cohort	
	n	Incidence/100,000 py (95% CI)	n	Mortality/100,000 py (95% CI)	n	Case-fatality rate/100 py (95% CI)
**Person-years (py) at risk**	137,986,981		138,068,520		58,517.92	
**Cohort**	27,900	20.22 (19.98–20.46)	9528	6.90 (6.76–7.04)	4040	6.90 (6.69–7.12)
**Follow-up time**						
Mean (sd)	4.87 (2.75)		4.88 (2.75)		2.62 (2.15)	
Median (IQR)	4.59 (2.49–7.51)		4.60 (2.49–7.52)		2.21 (0.75–4.02)	
**Block 1—geographic factors**						
Region of residence	N = 28,316,715		N = 28,316,748		N = 22,334	
North	2533	16.93 (16.28–17.60)	751	5.02 (4.67–5.39)	339	6.57 (5.90–7.30)
Northeast	7992	15.66 (15.32–16.00)	2372	4.64 (4.46–4.84)	1193	6.71 (6.34–7.11)
Southeast	9480	20.27 (19.86–20.68)	3937	8.41 (8.15–8.68)	1383	7.46 (7.07–7.86)
South	5958	40.07 (39.06–41.10)	1875	12.59 (12.04–13.18)	819	6.27 (5.85–6.71)
Mid-West	1933	18.73 (17.92–19.59)	593	5.74 (5.30–6.23)	306	7.71 (6.89–8.62)
Area of residence	N = 28,226,360		N = 28,316,748		N = 22,084	
Rural	2497	8.08 (7.77–8.40)	798	2.58 (20.41–2.77)	387	7.25 (6.56–8.01)
Urban	25,103	23.57 (23.28–23.86)	8657	8.12 (7.95–8.30)	3626	6.89 (6.66–7.11)
**Block 2—socioeconomic status**						
Education	N = 25,790,719		N = 25,790,750		N = 19,430	
Illiterate/never went to school	2329	17.68 (16.98–18.42)	1011	7.67 (7.21–8.16)	448	10.18 (9.28–11.17)
Literate/pre-school	161	14.13 (12.11–16.49)	48	4.21 (31.17–5.59)	19	6.11 (3.90–9.58)
Elementary school	17,186	22.18 (21.85–22.51)	6291	8.11 (7.91–8.32)	2604	7.30 (7.02–7.58)
High school	4413	16.86 (16.37–17.36)	1096	4.18 (3.94–4.44)	471	5.48 (5.01–6.00)
Higher education	353	20.94 (18.87–23.24)	69	4.09 (3.23–5.18)	27	3.94 (2.70–5.75)
Race/skin color	N = 26,703,594		N = 26,703,624		N = 21,105	
White/Asian	8663	20.80 (20.37–21.24)	2972	7.13 (6.88–7.39)	1200	6.45 (6.10–6.83)
Brown	14,215	18.32 (18.02–18.62)	4721	6.08 (5.61–6.26)	2092	7.01 (6.71–7.31)
Black	3429	32.51 (31.44–33.61)	1347	12.76 (12.09–13.46)	534	7.80 (7.17–8.49)
Indigenous	103	13.05 (10.76–15.83)	33	4.18 (2.97–5.88)	11	4.69 (2.60–8.47)
Wealth levels	N = 28,310,890		N = 28,310,923		N = 22,331	
Level 1 (Higher wealth)	1731	18.77 (17.91–19.68)	501	5.43 (4.98–5.93)	184	7.16 (6.19–8.27)
Level 2	3704	19.12 (18.52–19.75)	1227	6.33 (5.99–6.70)	506	9.01 (8.26–9.83)
Level 3	2858	20.47 (19.74–21.24)	947	6.78 (6.36–7.23)	383	8.18 (7.40–9.04)
Level 4	7793	21.33 (20.87–21.81)	2709	7.41 (7.14–7.70)	1150	6.55 (6.18–6.94)
Level 5 (Lower wealth)	11,807	20.05 (19.70–20.42)	4142	7.03 (6.82–7.25)	1815	6.47 (6.18–6.77)
Receipt time of *Bolsa Família*	N = 28,318,190		N = 28,318,223		N = 22,338	
Does not receive	6176	18.19 (17.75–18.65)	2227	6.56 (6.29–6.84)	856	8.24 (7.71–8.82)
Less than 2 years	4168	25.45 (24.69–26.23)	1678	10.24 (9.76–0.74)	683	9.54 (8.85–10.28)
Between 2 and 5 years	6342	24.76 (24.16–25.38)	2373	9.26 (8.89–9.64)	1000	8.18 (7.68–8.70)
Between 5 and 10 years	8323	19.96 (19.54–20.40)	2462	5.90 (5.67–6.14)	1136	5.44 (5.13–5.77)
More than 10 years	2891	14.20 (13.69–14.72)	788	3.87 (3.61–4.15)	365	4.64 (4.19–5.14)
**Block 3—household conditions**						
Household material	N = 27,553,988		N = 27,554,016		N = 21,704	
Brick	21,226	20.11 (19.84–20.38)	7317	6.93 (6.77–7.09)	3054	6.87 (6.63–7.12)
Wood	3844	25.02 (24.24–25.82)	1309	8.51 (8.06–8.99)	597	7.10 (6.55–7.69)
Mud and others	2033	13.87 (13.28–14.49)	652	4.45 (4.12–4.80)	303	6.56 (5.86–7.34)
Electrical power	N = 27,554,428		N = 27,554,456		N = 21,701	
Yes	22,899	19.42 (19.17–19.67)	7748	6.57 (6.42–6.71)	3270	6.74 (6.52–6.98)
No	4201	23.82 (23.11–24.55)	1528	8.66 (8.23–9.10)	682	7.59 (7.04–8.19)
Household density (residents/room)	N = 25,864,364		N = 25,864,390		N = 20,271	
Up to 2	23,511	19.68 (19.43–19.94)	8045	6.73 (6.59–6.88)	3409	6.83 (6.10–7.07)
More than 2	1807	24.15 (23.06–25.29)	639	8.53 (7.90–9.22)	297	7.14 (6.38–8.01)
Water supply	N = 27,554,446		N = 27,554,474		N = 21,701	
Public network	21,922	22.18 (21.89–22.47)	7476	7.56 (7.39–7.73)	3149	6.80 (6.56–7.04)
Others	5177	14.10 (13.72–14.49)	1799	4.90 (4.68–5.13)	803	7.22 (6.73–7.73)
Waste disposal	N = 27,554,109		N = 27,554,137		N = 21,702	
Public collection	23,870	22.90 (22.61–23.19)	8191	7.85 (7.68–8.02)	3463	6.85 (6.62–7.08)
Not collected	3230	10.31 (9.96–10.67)	1085	3.46 (3.26–3.67)	490	7.12 (6.51–7.77)
Sewage system	N = 26,969,245		N = 26,969,272		N = 21,493	
Sewage network	15,474	24.08 (23.70–24.46)	5506	8.56 (8.34–8.79)	2181	6.66 (6.39–6.95)
Septic tank	3723	18.39 (17.81–18.99)	1168	5.77 (5.44–6.11)	539	6.65 (6.11–6.95)
Others	7633	15.43 (15.09–15.78)	2504	5.06 (4.87–5.26)	1195	7.32 (6.92–7.75)
**Block 4—health and behavioral aspects**						
Treatment					N = 22,338	
Yes	–	–	–	–	2855	5.62 (5.42–5.83)
No	–	–	–	–	1185	15.39 (14.54–16.29)
Exposure category					N = 18,823	
Homosexual	–	–	–	–	224	3.91 (3.43–4.45)
Bisexual	–	–	–	–	107	4.60 (3.81–5.56)
Heterosexual	–	–	–	–	2414	5.79 (5.56–6.02)
People who inject drugs	–	–	–	–	210	11.56 (10.10–13.23)
Means of transmission					N = 18,849	
Blood transfusion					4	7.725 (2.90–20.58)
Accident with biological material					0	–
Sexual, with person of the opposite sex					2413	5.78 (5.56–6.02)
Sexual, with person of the same sex or both					332	4.12 (3.70–4.59)
Injecting drugs					211	11.54 (10.08–13.20)

Abbreviations: PLWA: people living with AIDS; CI: confidence interval; sd: standard deviation; IQR: interquartile range.

“-” variable not included in the model.

Table 1 shows AIDS-outcomes stratified by the main variables of interest. The measures of crude associations between strata are shown in Supplementary Table S11 (pp. 36–38).

### Effect of the main SDH on AIDS outcomes

The hierarchical approach allowed the selection of the SDH that produce significant effects for AIDS outcomes. For the AIDS incidence and mortality outcomes, the same set of variables was maintained in the final models: all variables from blocks 1 (geographical factors) and 2 (socioeconomic status) of analysis were maintained in the final models; for block 3 (household conditions), the variables water supply and waste disposal were excluded in the intra-block analyses (models 1) and the variable sewage system was excluded in the final inter-block analyses (models 3), all other variables were maintained in the final models (Table 2).

**Table 2:** Effect of social determinants of health on AIDS incidence and mortality rates in Brazil, results of Hierarchy Analysis using multivariable Poisson Regression adjusted for gender, age, year of admission to the cohort and municipal HIV/AIDS endemicity and mortality in the period (2007–2015).

	AIDS incidence rate					AIDS mortality rate				
	Model 1			Model 2	Model 3	Model 1			Model 2	Model 3
	Intra-block analyses			Inter-block analyses (Blocks 1 + 2)	Inter-block analyses (Blocks 1 + 2 + 3)	Intra-block analyses			Inter-block analyses (Blocks 1 + 2)	Inter-block analyses (Blocks 1 + 2 + 3)
	RR (95% CI)			RR (95% CI)	RR (95% CI)	RR (95% CI)			RR (95% CI)	RR (95% CI)
**Block 1—geographic factors**	N = 28,219,514			N = 24,673,018	N = 22,468,882	N = 28,219,545			N = 24,673,044	N = 22,468,903
Region of residence	a			a	a	a			a	a
North	1.00			1.00	1.00	1.00			1.00	1.00
Northeast	1.09 (0.99–1.20)			1.10 (1.00–1.20)	1.22 (1.11–1.35)	0.98 (0.89–1.08)			0.98 (0.89–1.09)	1.16 (1.03–1.31)
Southeast	1.09 (0.99–1.20)			1.13 (1.03–1.23)	1.26 (1.15–1.39)	1.20 (1.04–1.39)			1.23 (1.05–1.45)	1.46 (1.20–1.78)
South	1.29 (1.17–1.43)			1.41 (1.70–1.55)	1.48 (1.35–1.63)	1.16^a^ (0.99–1.35)			1.24 (1.04–1.47)	1.36 (1.14–1.63)
Mid-West	1.15 (1.02–1.30)			1.16 (1.03–1.32)	1.28 (1.13–1.45)	1.03 (0.91–1.16)			1.06 (0.92–1.22)	1.21 (1.03–1.42)
Area of residence										
Rural	1.00			1.00	1.00	1.00			1.00	1.00
Urbana	1.93 (1.81–2.05)			2.05 (1.92–2.20)	2.17 (2.02–2.33)	2.03 (1.84–2.24)			2.17 (1.96–2.40)	2.35 (2.11–2.62)
**Block 2—socioeconomic status**	N = 24,706,061					N = 24,710,440				
Education	a			a	a	a			a	a
Illiterate/never went to school	1.38 (1.19–1.60)			1.51 (1.31–1.73)	1.46 (1.26–1.68)	2.67 (1.97–3.63)			2.93 (2.14–4.02)	2.76 (1.99–3.82)
Literate/pre-school	1.04 (0.84–1.29)			1.12 (0.91–1.38)	1.10 (0.89–1.36)	1.48 (1.00–2.21)			1.59 (1.06–2.40)	1.46 (0.95–2.25)
Elementary school	1.41 (1.22–1.62)			1.45 (1.27–1.65)	1.43 (1.25–1.65)	2.55 (1.90–3.44)			2.58 (1.92–3.48)	2.50 (1.83–3.41)
High school	0.95 (0.84–1.07)			0.96 (0.85–1.08)	0.97 (0.85–1.09)	1.30 (0.94–1.78)			1.28 (0.93–1.75)	1.23 (0.88–1.71)
Higher education	1.00			1.00	1.00	1.00			1.00	1.00
Race/skin color	a			a	a	a			a	a
White/Asian	1.00			1.00	1.00	1.00			1.00	1.00
Brown	1.09 (1.04–1.14)			1.17 (1.12–1.23)	1.17 (1.12–1.22)	1.12 (1.05–1.20)			1.20 (1.12–1.28)	1.19 (1.12–1.27)
Black	1.51 (1.42–1.60)			1.56 (1.48–1.65)	1.53 (1.45–1.61)	1.73 (1.57–1.90)			1.75 (1.62–1.89)	1.69 (1.57–1.83)
Indigenous	0.81 (0.64–1.04)			1.16 (0.91–1.46)	1.12 (0.87–1.43)	0.87 (0.61–1.25)			1.25 (0.87–1.80)	1.20 (0.82–1.75)
Wealth levels	a			a	a	a			a	a
Level 1 (Higher wealth)	1.00			1.00	1.00	1.00			1.00	1.00
Level 2	1.08 (1.01–1.14)			1.09 (1.03–1.16)	1.07 (1.01–1.14)	1.20 (1.08–1.34)			1.23 (1.10–1.37)	1.17 (1.04–1.32)
Level 3	1.24 (1.14–1.34)			1.29 (1.20–1.39)	1.27 (1.17–1.37)	1.40 (1.20–1.63)			1.48 (1.29–1.71)	1.42 (1.21–1.66)
Level 4	1.34 (1.23–1.47)			1.37 (1.27–1.48)	1.32 (1.23–1.43)	1.60 (1.40–1.82)			1.66 (1.47–1.88)	1.58 (1.36–1.82)
Level 5 (Lower wealth)	1.56 (1.41–1.72)			1.60 (1.48–1.73)	1.55 (1.43–1.68)	1.94 (1.68–2.25)			2.08 (1.81–2.40)	1.99 (1.70–2.34)
Receipt time of *Bolsa Família*^a^				a	a	a			a	a
Does not receive	1.00			1.00	1.00	1.00			1.00	1.00
Less than 2 years	1.11 (1.05–1.18)			1.11 (1.05–1.17)	1.08 (1.03–1.14)	1.23 (1.14–1.32)			1.22 (1.13–1.31)	1.18 (1.08–1.29)
Between 2 and 5 years	1.04 (0.99–1.09)			1.05^a^ (1.00–1.11)	1.05^a^ (1.00–1.10)	1.05 (0.96–1.15)			1.06 (0.97–1.15)	1.05 (0.96–1.14)
Between 5 and 10 years	0.83 (0.79–0.88)			0.87 (0.83–0.92)	0.86 (0.81–0.91)	0.64 (0.60–0.69)			0.68 (0.63–0.73)	0.66 (0.61–0.72)
More than 10 years	0.67 (0.62–0.72)			0.72 (0.68–0.77)	0.70 (0.65–0.75)	0.44 (0.40–0.49)			0.49 (0.44–0.54)	0.47 (0.42–0.52)
**Block 3—household conditions**	N = 25,267,933					N = 25,267,958				
Household material	a				a	a				a
Brick	1.00				1.00	1.00				1.00
Wood	1.23 (1.16–1.29)			–	1.21 (1.15–1.28)	1.27 (1.16–1.38)			–	1.31 (1.19–1.43)
Mud and others	1.11 (1.04–1.18)			–	1.07 (1.00–1.14)	1.04 (0.94–1.15)			–	1.10 (0.99–1.21)
Electrical power										
Yes	1.00				1.00	1.00				1.00
No	1.19 (1.13–1.25)			–	1.15 (1.09–1.21)	1.31 (1.20–1.43)			–	1.24 (1.16–1.33)
Household density (residents/room)										
Up to 2	1.00				1.00	1.00				1.00
More than 2	1.15 (1.07–1.23)			–	1.15 (1.09–1.22)	1.23 (1.13–1.35)			–	1.23 (1.12–1.35)
Water supply										
Public network	1.00					1.00				
Others	0.92 (0.87–0.97)			–	–	0.97 (0.90–1.06)			–	–
Waste disposal										
Public collection	1.00					1.00				
Not collected	0.63 (0.59–0.67)			–	–	0.60 (0.54–0.67)			–	–
Sewage system	a					a				
Sewage network	1.00					1.00				
Septic tank	0.95 (0.90–1.00)			–	–	0.89 (0.81–0.97)			–	–
Others	0.93 (0.89–0.97)			–		0.87 (0.81–0.94)			–	–
**Model analysis**	Block 1	Block 2	Block 3			Block 1	Block 2	Block 3		
Mean VIF	2.14	2.78	1.63	3.09	3.07	2.12	2.77	1.62	3.07	3.06
Deviance goodness-of-fit (p-value)	1.00	1.00	1.00	1.00	1.00	1.00	1.00	1.00	1.00	1.00

Abbreviations: CI: confidence interval; RR: rate ratios.

“-” variable not included in the model.

a Overall p-value ≤0.05.

For the AIDS case-fatality rates outcome, all variables from blocks 2 (socioeconomic status) and 4 (health and behavioral aspects) of the analysis were maintained in all hierarchical selection steps. The variable area of residence (block 1: geographical factors) was excluded in the final inter-block analyses (model 4). For block 3 (household conditions), the intra-block analyses (model 1) suggested the exclusion of the variables household material, water supply and waste disposal, and in the final model (model 4) the variable electrical power was also excluded, while the other variables (household density and sewage system) were maintained in the final model (model 4) (Table 3).

**Table 3:** Effect of social determinants of health on AIDS case-fatality rate over the study period in Brazil, results of Hierarchy Analysis using multivariable Poisson Regression adjusted for gender, age, year of admission to the cohort and municipal HIV/AIDS endemicity and mortality in the period (2007–2015).

	Model 1				Model 2	Model 3	Model 4
	Intra-block analyses				Inter-block analyses (Blocks 1 + 2)	Inter-block analyses (Blocks 1 + 2 + 3)	Inter-block analyses (Blocks 1 + 2+3 + 4)
	RR (95% CI)				RR (95% CI)	RR (95% CI)	RR (95% CI)
**Block 1—geographic factors**	N = 18,583				N = 15,674	N = 14,085	N = 11,815
Region of residence							b
North	1.00				1.00	1.00	1.00
Northeast	0.98 (0.85–1.15)				1.00 (0.85–1.19)	1.11 (0.93–1.34)	0.96 (0.76–1.21)
Southeast	0.97 (0.80–1.17)				1.00 (0.80–1.25)	1.16 (0.91–1.49)	0.81 (0.64–1.03)
South	0.86 (0.73–1.00)				0.88 (0.73–1.06)	1.02 (0.84–1.24)	0.92 (0.71–1.18)
Mid-West	0.98 (0.81–1.19)				1.04 (0.84–1.29)	1.13 (0.90–1.42)	1.09 (0.82–1.45)
Area of residence							
Rural	1.00				1.00	1.00	
Urbana	0.96 (0.82–1.12)				1.01 (0.85–1.20)	1.14 (0.94–1.39)	–
**Block 2—socioeconomic status**	N = 15,836						
Education	a				a	a	a
Illiterate/never went to school	2.65 (1.56–4.51)				2.56 (1.48–4.40)	2.30 (1.31–4.01)	1.86 (1.02–3.41)
Literate/pre-school	1.46 (0.68–3.15)				1.41 (0.65–3.09)	1.32 (0.61–2.88)	1.23 (0.52–2.90)
Elementary school	2.32 (1.39–3.86)				2.25 (1.34–3.78)	2.04 (1.19–3.50)	1.73 (0.97–3.06)
High school	1.76 (1.02–3.02)				1.70^a^ (0.98–2.93)	1.49 (0.84–2.64)	1.35 (0.75–2.42)
Higher education	1.00				1.00	1.00	1.00
Race/skin color	a				a	b	
White/Asian	1.00				1.00	1.00	1.00
Brown	1.16 (1.06–1.27)				1.10 (0.99–1.22)	1.10 (0.99–1.22)	1.09 (0.96–1.24)
Black	1.24 (1.10–1.40)				1.19 (1.05–1.33)	1.16 (1.03–1.32)	1.14 (1.01–1.30)
Indigenous	0.68 (0.28–1.64)				0.66 (0.27–1.59)	0.74 (0.30–1.82)	0.58 (0.22–1.55)
Wealth levels							
Level 1 (Higher wealth)	1.00				1.00	1.00	1.00
Level 2	1.25 (1.03–1.51)				1.25 (1.04–1.51)	1.23^a^ (1.00–1.53)	1.18 (0.90–1.53)
Level 3	1.20 (0.91–1.57)				1.20 (0.92–1.56)	1.14 (0.86–1.52)	1.07 (0.79–1.45)
Level 4	1.14 (0.93–1.39)				1.16 (0.94–1.43)	1.16 (0.91–1.47)	1.01 (0.78–1.32)
Level 5 (Lower wealth)	1.18 (0.95–1.48)				1.22^a^ (0.97–1.54)	1.23 (0.95–1.61)	1.09 (0.81–1.45)
Receipt time of *Bolsa Família*	a				a	a	a
Does not receive	1.00				1.00	1.00	1.00
Less than 2 years	1.28 (1.08–1.52)				1.29 (1.10–1.52)	1.28 (1.08–1.53)	1.23 (1.02–1.50)
Between 2 and 5 years	1.13^a^ (0.98–1.31)				1.15 (1.01–1.32)	1.11 (0.97–1.28)	1.02 (0.87–1.21)
Between 5 and 10 years	0.86 (0.74–0.99)				0.87 (0.75–0.99)	0.83 (0.71–0.96)	0.77 (0.65–0.91)
More than 10 years	0.69 (0.58–0.83)				0.70 (0.581–0.84)	0.66 (0.55–0.80)	0.57 (0.46–0.72)
**Block 3—household conditions**	N = 16,856						
Household material							
Brick	–				–	–	–
Wood	–				–	–	–
Mud and others	–				–	–	–
Electrical power							
Yes	1.00					1.00	
No	1.16 (1.02–1.31)				–	1.13 (1.01–1.27)	–
Household density (residents/room)							
Up to 2	1.00					1.00	1.00
More than 2	1.21 (1.05–1.40)				–	1.23 (1.05–1.43)	1.35 (1.12–1.63)
Water supply							
Public network	–				–	–	–
Others	–				–	–	–
Waste disposal							
Public collection	–				–	–	–
Not collected	–				–	–	–
Sewage system	b					a	a
Sewage network	1.00					1.00	1.00
Septic tank	1.07 (0.94–1.23)				–	1.10 (0.96–1.26)	1.05 (0.89–1.24)
Others	1.13 (1.02–1.25)				–	1.14 (1.02–1.28)	1.17 (1.03–1.33)
**Block 4—health and behavioral aspects**	N = 15,785						
Treatment							
Yes	1.00						1.00
No	2.50 (2.13–2.92)				–	–	2.58 (2.22–3.00)
Exposure category	a						a
Homosexual	1.00				–	–	1.00
Bisexual	1.10 (0.86–1.40)				–	–	1.06 (0.79–1.43)
Heterosexual	1.67 (1.40–1.98)						1.56 (1.29–1.90)
People who inject drugs	2.98 (2.38–3.72)				–	–	3.05 (2.36–3.94)
**Model analysis**	Block 1	Block 2	Block 3	Block 4			
Mean VIF	3.62	3.96	2.24	3.70	4.54	4.40	4.04
Deviance goodness-of-fit (p-value)	≤0.001	≤0.001	≤0.001	≤0.001	≤0.001	≤0.001	≤0.001

Abbreviations: CI: confidence interval; RR: rate ratio.

“-” variable not included in the model.

a Overall p-value ≤0.05.

b Overall p-value ≤0.10.

From these selection steps, we obtained the final models with the adjusted effect of SDH on AIDS incidence, mortality (Table 2), and case-fatality rates (Table 3).

#### (a) Geographic factors

The region of residence was associated with incidence and mortality; individuals living in the South (rate ratios—RR: 1.48; 95% confidence interval—CI: 1.35–1.63) or Central-West (RR: 1.28; 95% CI: 1.13–1.45) of Brazil were most likely to become AIDS cases. People living in the Southeast (RR: 1.46; 95% CI: 1.20–1.78) and South (RR: 1.36; 95% CI: 1.14–1.63) of Brazil experienced a higher risk of AIDS-related mortality (Table 3). The area of residence also influences the risk of becoming an AIDS case, and subsequent mortality, with individuals living in urban areas experiencing a higher risk of becoming an AIDS case (RR: 2.17; 95% CI: 2.02–2.33) and suffering an AIDS-related death (RR: 2.35; 95% CI: 2.11–2.62) (Table 2).

#### (b) Socioeconomic status

With regards to socioeconomic indicators, illiterate people had a 46% higher risk of becoming an AIDS case (RR: 1.46; 95% CI: 1.26–1.68) and a 176% higher risk of an AIDS-related death (RR: 2.76; 95% CI: 1.99–3.82), when compared to those who had completed higher education. Similarly, the risk was higher for those who had only completed elementary school, both for incidence (RR: 1.43; 95% CI: 1.25–1.65) and mortality (RR: 2.50; 95% CI: 1.83–3.41) (Table 2). For people living with AIDS (PLWA), illiteracy increased the risk of the case-fatality rate by 130% (RR: 2.30; 95% CI: 1.31–4.01), and not having completed primary education increased this rate by 104% (RR: 2.04; 95% CI: 1.19–3.50) (Table 3).

Black people had a 53% higher risk of becoming an AIDS case (RR: 1.53; 95% CI: 1.45–1.61), and a 69% higher risk of an AIDS-related death (RR: 1.69; 95% CI: 1.57–1.83) than those who self-identified as white or of Asian heritage (Table 2). Among PLWA, individuals who self-identified as black had higher case-fatality rates (RR: 1.16; 95% CI: 1.03–1.32) than those who self-identified as white or of Asian heritage (Table 3).

Wealth was closely related to AIDS outcomes, with the risk strongly increasing as the wealth level decreases. Individuals with lower wealth levels were 55% more likely to become an AIDS case (RR: 1.55; 95% CI: 1.43–1.68), and 99% more likely to die (RR: 1.99; 95% CI: 1.70–2.34) (Table 2). Among PLWA, wealth loses statistical significance, but the poorest categories (lower wealth) had worse outcomes than those with greater wealth (Table 3).

#### (c) Receipt of government conditional cash transfer programs—*Bolsa Família*

The duration of *Bolsa Família* receipt also showed associations with all outcomes under study. Individuals who received *Bolsa Família* for less than two years had a higher risk of becoming ill (RR: 1.08; 95% CI: 1.03–1.14) and dying from AIDS (RR: 1.18; 95% CI: 1.08–1.29), when compared to those who did not meet the criteria for receiving the benefit. A protective effect was observed for long-term receipt, with those receiving the benefit for between 5 and 10 years, or more than 10 years, having a lower risk of becoming ill (RR: 0.86, 95% CI: 0.81–0.91; RR: 0.70, 95% CI: 0.65–0.75, respectively) and dying from AIDS (RR: 0.66; 95% CI: 0.61–0.72; RR: 0.47; 95% CI: 0.42–0.52, respectively) (Table 2). Among PLWA, similar results are found, with a higher risk of case-fatality rate for those who had received the benefit for less than two years (RR: 1.28; 95% CI: 1.08–1.53), and lower risk for those who had received the benefit longer: either between 5 and 10 years (RR: 0.83; 95% CI: 0.71–0.96), or more than 10 years (RR: 0.66; 95% CI: 0.55–0.80) (Table 3).

### Sensitivity analyses

All sensitivity analyses produced similar estimates, demonstrating the robustness of the results. Different model specifications—in terms of alternative sets of individual-level and aggregate-level variables—did not significantly change the SDH effect estimates, and analyses performed solely with a small set of municipalities with a high quality of vital information, produced similar results. In the analyses for potential changes over time of the association between SDH and AIDS-related outcomes, the effects of all SDH remained similar throughout the study period. In some SDH, such as education, race/skin color, wealth levels, and length of time receiving Bolsa Família, there was a slight effect on the increase (Supplementary Tables S7 and S8 in the appendix, pp. 21–25). The complete results of the extensive sensitivity analyses performed are available in the Appendix.

## Discussion

Our study used a cohort of 28.3 million Brazilians, over 9 years, to comprehensively evaluate the effects of major SDH on AIDS morbidity and mortality. To our knowledge, this study is the largest and most comprehensive evaluation of the effects of SDH on infectious diseases and, in particular, HIV/AIDS. We found an exceptionally strong effect for black people and a dose-response relationship for the wealth level and educational attainment on AIDS incidence, mortality, and case-fatality rates, in addition to statistically significant effects related to geographic factors and several other SDH. Lower wealth may characterize socio-structural resource limitations, and be closely related to social exclusion and food insecurity, which are potential barriers to early diagnosis, and initiation, or adherence to HIV/AIDS treatment.^6^^,^^24^ There is also evidence that poverty, in addition to being associated with an HIV risk, is related to reduced access to treatment and poorer health outcomes among PLWHA.^25^

Our results reveal strong effects of educational attainment on the three AIDS outcomes assessed. These significant relationships may be explained by factors ranging from access, understanding, and ability to act on health information (including prevention, importance of testing, and treatment), different sexual risk behaviors, and access to healthcare. Previous studies have shown that individuals with less education tend to be exposed to more risky sexual behaviors,^22^ have a higher risk of late HIV/AIDS diagnosis,^9^ and experience less access and adherence to ART.^6^

In our study, people who self-identified as black had higher AIDS-related incidence, mortality, and case-fatality rates. Similarly, in high-income countries, incidence and mortality^25^ are higher among black people. This higher risk is considered a consequence of structural racism, including racial health inequalities for access, and the quality of healthcare services,^26^ since there is no evidence that genetic factors are responsible.^25^ Structural racism in Brazil, as in many other parts of the world, also manifests in worse housing, lower paid employment, and unfair labor conditions, further perpetuating racial inequalities and subjecting people to worse HIV-related outcomes overall.^27^

With regards to other SDH, household-related conditions (household construction material, electricity, and household density) had significant relationships with AIDS-related incidence, mortality, and case-fatality rates, with higher risks among those living in conditions of lower socioeconomic status.^12^ Brazilian regions with greater development and infrastructure (South and Southeast), and urban areas, were also associated with a higher HIV/AIDS burden. In addition to providing greater access to health services and information,^28^ and therefore greater potential to detect the disease,^29^ large cities in these regions are characterized by a range of factors, including health behaviors that may contribute to higher HIV/AIDS morbidity.^12^

Analysis of the Bolsa Família conditional cash transfer (CCT) program, showed expected effects. For individuals who received a CCT for a short period of time (less than 2 years), a positive association was observed with AIDS outcomes, which may be related to the characteristics of greater social vulnerability of individuals eligible for the program. On the other hand, for those who received the CCT for a longer period of time (5 years or more), a protective effect was observed, revealing a longer-term positive effect of the program for AIDS outcomes. This may be due to the poverty-relief effect of the program’s cash allowances, and conditionalities to receive these benefits, such as the need to monitor their health at primary health care services, and school attendance for children and adolescents. This brings families closer to the services that make the diagnosis, and health education actions, which enable advancement in the level of schooling. Systematic reviews have already demonstrated that the CCT may reduce risky sexual behaviors (such as sex work), and consequent HIV infection.^30^

In the analyses of factors associated with AIDS case-fatality rates, the effects of several SDH decreased when we included behavioral and other proximal variables in the model (indicated as Block 4 in the analysis). This was expected, due to their mediation effects^23^: that is, when including variables that are part of the causal pathway in regression models which focus on evaluating the effects of distal determinants—such as SDH—on HIV/AIDS, these mediating variables decrease the values of the statistical association between SDH and AIDS-outcomes. However, if the goal of the analyses is to evaluate the direct effects of SDH on AIDS, these mediating factors should not be included in statistical models. When we focused on these proximal factors, it was observed that persons who inject drugs (PWID), or did not receive ART, had a higher risk of dying from AIDS. Moreover, individuals who self-identified as heterosexual were more likely to experience death than their counterparts. These associations were expected, since PWID are most often poor and have more difficulties in adhering to treatment.^10^ It is expected that, without ART, almost all individuals with AIDS would die, but the timeframe necessary to estimate this result would require longer cohorts.^31^

Our results reveal that the entire chain of AIDS-related events is influenced by SDH, whether through a higher risk of infection (due to economic, cultural, or geographic barriers to preventive measures, and higher-risk sexual behaviors), or a greater risk of acquiring an advanced disease, and dying from it (due to less access to diagnosis, early treatment, advanced healthcare, and hospitalization). It is understood, therefore, that SDH, especially wealth levels, education, and race/skin color, are strongly associated with the dynamics of HIV/AIDS infection and survival.

This study has a number of limitations. The first is that the study cohort focuses on the poorest half of the Brazilian population. While this could limit the representativeness of the study population, it may also be considered a strength of our analyses, considering that SDH mainly exert their effects on individuals with a lower socioeconomic status. This large cohort includes a high number of extremely poor individuals, who are usually not considered, or underrepresented, in traditional, observational, or randomized studies.

Another limitation is heterogeneity in Brazil’s 5570 municipalities’ ability to deliver effective and timely AIDS diagnoses, as well as adequate access to health services and follow-up of PLWHA. Thus, living in a municipality with higher or lower endemicity, health surveillance capacity, health infrastructure, and socio-environmental conditions may contribute to different levels of detection and management of HIV/AIDS outcomes. In order to correct for this possible bias, first we assessed the dependency of outcomes at the municipal, state, or region of residence level. Second, we assessed the adjustment for county variables representing health infrastructure and socio-environmental conditions. And third, we evaluated estimates restricted to counties with adequate quality of vital information. We detected no change in the estimates for the different models evaluated as a sensitivity analysis (Supplementary Tables S1–S8 in the appendix, pp. 7–25). Thus, we opted for more conservative models (Poisson regression), including all municipalities—to ensure external validity—and the entire period of analysis—to ensure the longest follow-up period. Analyses for specific sub-periods of time showed similar effects of SDH on AIDS-related outcomes, and enable us to infer that changes in health policies aimed at HIV/AIDS do not alter our findings, and AIDS outcomes should still be influenced by SDHs after 2015.

The analysis of case-fatality rates also has specific limitations, since AIDS may have a relatively long survival period.^31^ Even if higher socioeconomic vulnerability could be associated with a greater severity of the disease, and decreased survival time, there is the possibility of underestimation of the real AIDS case-fatality rate. Nevertheless, the long study and potential follow-up period—that reached almost 9 years in some cases—of AIDS cases, allowed us to obtain reliable estimates of the influence of SDH on case-fatality rates over the period. Moreover, the associations between SDH and AIDS case-fatality rates showed similar expected results as associations with other outcomes, demonstrating the robustness of the analyses along the causal chain of all the disease outcomes.

Our results show—for the first time in such a comprehensive way and with a large number of individuals—that SDH play a major role in the burden of HIV/AIDS in a highly unequal MIC, such as Brazil, and wealth, education, and race have a particularly strong effect on AIDS incidence, mortality, and case-fatality rates.

These findings have important implications for HIV/AIDS prevention and control programs in other LMICs. This study lends evidence to assertions that investments to reduce social inequalities, with a focus on the most important SDH that affect HIV/AIDS, should be promoted and coupled with current biomedical and behavioral interventions. Investments in public health policies are required, to expand access, and ensure equity of care, allowing those with greater social vulnerabilities to be prioritized. HIV/AIDS policies also need to broaden their view on social vulnerabilities and health prevention actions, and the discrimination that the poor, black people, and the illiterate suffer to access health services, should be effectively addressed.

Without the implementation of significant interventions to improve SDH, especially social protection programs, there is a risk that the current increase in poverty rates and social vulnerabilities—partly due to the COVID-19 pandemic, the effects of the war in the Ukraine, and the global inflation crisis—could reverse progress made in the fight against HIV/AIDS over the last decades, and hamper achievement of the HIV/AIDS-related Sustainable Development Goals.

## Contributors

DR, ID, LES, and JM developed the study concept. MLB, MYI, and CASTS collected the data. IL, DR, AFS, and JP designed the study and investigation. IL, AFS, LM, and NSG have accessed and verified the data. IL, AFS, LM, and NSG analyzed the data, and wrote the first draft. DR, ID, LES, JM, and LM supervised and validated the study process. RA managed the project, resources, and software. All authors had access to all the data reported in the study. All authors contributed to data interpretation and validation, reviewed, and edited the manuscript.

## Data sharing statement

The protocol for creation of the 100 Million Brazilians Cohort, and the 100 Million Brazilians Cohort profile is available in the publications referenced in the article. Further material is available at: https://cidacs.bahia.fiocruz.br/en/platform/cohort-of-100-millionbrazilians. The linkage protocols are explained in the referenced publications, and the codes are available at: https://gitHub.com/gcgbarbosa/cidacs-rl. Individual-level data will not be available for sharing, due to confidentiality and ethical issues.

## Declaration of interests

The authors declare no competing interests.
