# Supplementary material for: The effects of social determinants of health on acquired immune deficiency syndrome in a low-income population of Brazil: a retrospective cohort study of 28.3 million individuals
Source: Lancet Reg Health Am. 2023 Jul 17;24:100554. doi: 10.1016/j.lana.2023.100554 (PMC10372893; doi:10.1016/j.lana.2023.100554)
Supplement: Supplementary Material [file mmc1.pdf]

SUPPLEMENTARY MATERIAL

The effects of social determinants of health on acquired immune deficiency syndrome in a low-income population of Brazil: a retrospective cohort study of 28.3 million individuals

Iracema Lua, PhD; Andrea F Silva, PhD; Nathalia Sernizon Guimarães, PhD; Laio Magno, PhD; Julia Pescarini, PhD; Rodrigo VR Anderle, PhD; Maria Y Ichihara, PhD; Mauricio L Barreto, PhD; Carlos AS Teles Santos, PhD; Louisa Chenciner, MD MSc; Luis Eugênio Souza, PhD; James Macinko, PhD; Ines Dourado, PhD; Davide Rasella, PhD

SUMMARY

**1. The '100 Million Brazilian Cohort'**	2
**2.Outcome Variables**	3
**Chart S1.** Definition criteria for the outcome variables	3
**Chart S2.** Formulas used to calculate AIDS incidence, mortality, and case-fatality rates	5
**3.Sensitivity Analyses**	7
**Table S1**. Sensitivity Analyses of model AIDS incidence rate: alternative regression models (N=22,468,882).	7
**Table S2.** Sensitivity Analyses of model AIDS mortality rate: alternative regression models (N=22,468,903).	9
**Table S3.** Sensitivity Analyses of model AIDS case-fatality rate over the study period: alternative regression models (N= 11,815).	11
**Table S4.** Sensitivity Analyses of model AIDS incidence rate: Quality of Vital Information and aggregate municipal variables for adjustment	13
**Table S5.** Sensitivity Analyses of model AIDS mortality rate: Quality of Vital Information and aggregate municipal variables for adjustment	16
**Table S6.** Sensitivity Analyses of model AIDS case-fatality rate over the study period: Quality of Vital Information and aggregate municipal variables for adjustment	18
**Table S7.** Sensitivity Analyses of model AIDS incidence rate: Effects over time	21
**Table S8.** Sensitivity Analyses of model AIDS mortality rate: Effects over time	24
**4. Descriptive analysis**	26
**Table S8.** Description of Brazilians aged 13 years and older followed (N = 28,318,532) from the 100 million cohort, Brazil, 2007-2015.	26
**Figure S1.** Annual loss-to-follow-up rates	29
**Table S9.** Description of Brazilians aged 13 years and older who died (n = 717,202) during the cohort, Brazil, 2007-2015.	30
**Table S10.** Analyses of missing data: comparing characteristics between the study population with complete and incomplete cases for analyses of the final models for each AIDS outcome.	32
**5.Crude Analyses:** Incidence, mortality, and case fatality rates from AIDS by SDH strata	35
**Table S11.** Incidence, mortality, and case fatality rates from AIDS in Brazilians aged 13 years or older (N = 28,318,532) recorded in CadÚnico from 2007 to 2015, stratified by geographic, socioeconomic, household characteristics and health and behavioral aspects (crude analyses).	36
**6. Trend of AIDS morbidity and mortality indicators**	39
**Figure S2.** Trend of AIDS morbidity and mortality indicators of Brazilians aged 13 years and older registered in *CadÚnico* in the period from 2007 to 2015.	40
**REFERENCES**	41

1. The '100 Million Brazilian Cohort'

The '100 Million Brazilian Cohort' has as its database the Single Registry of Brazilians for Social Programs (in Portuguese: Cadastro Único para Programas Sociais - CadÚnico), its baseline includes a total of 131,697,800 individuals, about 62% of the Brazilian population, who entered in different periods from 2001 to 2018.[1] The version used for this study comprises records from January 1, 2007 to December 31, 2015.

CadUnico is an administrative database that contains personal information (age, gender, race, education, and others) and household information (household density, family income, structural characteristics of the residence, and others) of low-income Brazilians, has 246 variables with demographic and socioeconomic information at the individual and family level. It is a social tool that identifies and characterizes individuals and their families, allowing the government to know the socioeconomic aspect of the poorest and use it for the selection of social programs.[1,2]

The cohort database is managed by the Center for Integration of Data and Knowledge in Health (in Portuguese: Centro de Integração de Dados e Conhecimento em Saúde - CIDACS / FIOCRUZ)[1], which aims to facilitate ongoing research and evaluation of social determinants and the effects of social policies and programs in health contexts in Brazil. To this end, linkage is made of these administrative records with the records of the health information systems (SIS) of the country, such as the National System of Disease Notification (SINAN) and the Mortality Information System (SIM),[1] including events related to HIV/AIDS.

The codes and algorithms for linking the databases are done by CIDACS, with assurance of the security of the identified data and two-step CIDACS-RL (https://gitHub.com/gcgbarbosa/cidacs-rl) use. Five identifying variables are used to perform probabilistic likage between the various databases: the date of birth, county of residence, gender, name, and mother's name of each individual presented in each of the databases.[1,3,4] For cases that are not identical the linkages are done based on a similarity score for all pairwise comparisons, ranging from 0 to 1, those with the highest similarity scores are considered to be linked pairs. The quality of each linkage for all causes between CadUnico, SINAN, and SIM was extensively evaluated and validated.[5,6]

For this study, the researchers responsible made additional linkage by including aggregate variables at the municipal level, using the IBGE code of the municipality of residence of the individual in the CadÚnico baseline and his/her year of entry into the cohort. This linkage allowed us to assess the health infrastructure in the municipality (e.g. coverage of Family Health Strategy - FHS, specialized clinics rate, physicians per 1K, hospital beds per 1K), epidemiological situation regarding the outcome under analysis (e.g. average outcome rate in the municipality of residence), socio-environmental conditions of the municipality (e.g. gini index, and extreme poverty and unemployment rates), and Quality of Vital Information. Thus expand the possibilities of studying the social determinants of health.

It is an database that allows the conduction of several social and health researches, however, it has some limitations regarding the completeness of the information, with biases of information from the interviewee since it is self-referred data, and with partial representativeness of the Brazilian population since it is composed of the portion of the population with greater social vulnerability.

2. Outcome Variables

The outcome variables of the models is available were i. new AIDS cases, according to the criteria established by the Brazilian Ministry of Health,[7] and ii. AIDS-related death, considering as underlying cause the International statistical classification of diseases and related health problems, 10th revision (ICD-10) codes that refer to AIDS (B20-B24)[8] (Chart S1).

Chart S1. Definition criteria for the outcome variables

AIDS OUTCOMES/ CRITERIA	DEFINITIONS	SPECIFICS
**New AIDS cases**		
1. Modified CDC Criteria	An AIDS case is any individual 13 years of age or older with laboratory evidence of HIV[a] infection and a diagnosis of at least one disease indicative of AIDS.	Diseases indicative of AIDS:<br>– Invasive cervical cancer,<br>– Candidosis of the esophagus<br>– Candidosis of the trachea, bronchus or lung,<br>– Cytomegalovirosis (except liver, spleen or lymph nodes),<br>– Extrapulmonary cryptococcosis,<br>– Chronic intestinal cryptosporidiosis > 1 month,<br>– Mucocutaneous herpes simplex > 1 month,<br>– Disseminated histoplasmosis,<br>– Chronic intestinal isosporidiosis > 1 month,<br>– Progressive multifocal leukoencephalopathy<br>– Non-Hodgkin's lymphoma and other lymphomas,<br>– Primary lymphoma of the brain<br>– Disseminated mycobacteriosis except tuberculosis and leprosy<br>– Pneumocystis carinii pneumonia,<br>– Reactivation of Chagas disease (meningoencephalitis and/or myocarditis),<br>– Salmonellosis (recurrent non-typhoidal sepsis),<br>– Cerebral toxoplasmosis,<br>– CD4+ T-lymphocyte count less than 350 cells/mm3.
2. Rio de Janeiro/Caracas Criteria	An AIDS case is any individual 13 years of age or older with laboratory evidence of HIV[a] infection and at least a total of 10 points, according to the scale of signs, symptoms or diseases	Scale of points:<br>– Kaposi's sarcoma (10)<br>– Disseminated/extra-pulmonary/non-cavitary tuberculosis (10)<br>– Oral Candidosis or Hairy Leukoplakia (5)<br>– Cavitary or unspecified pulmonary tuberculosis (5)<br>– Herpes zoster in individual younger than or equal to age 60 (5)<br>– Central nervous system dysfunction (5)<br>– Diarrhea equal or longer than 1 month (2)

		– Fever of 38ºC or more for 1 month or longer (2)*<br>– Caquexia or weight loss greater than 10% (2)*<br>– Asthenia greater than or equal to 1 month (2)*<br>– Persistent dermatitis (2)<br>– Anemia and/or lymphopenia and/or thrombocytopenia (2)<br>– Persistent cough or any pneumonia (2)*<br>– Lymphadenopathy greater than or equal to 1cm, greater than or equal to 2 extra-inguinal sites and for a time greater than or equal to 1 month (2)<br>*Tuberculosis is excluded as the cause
3. Death Criterion	An AIDS case is considered to be any individual aged 13 years or more, whose Death Certificate has mention of HIV infection or AIDS (or equivalent terms) in any of its fields, in addition to disease (s) associated with HIV infection. And who, after epidemiological investigation, cannot be fit into any of the other current AIDS case definition criteria.	-
**AIDS-related deaths**	Including those with ICD-10 deaths recorded as B20, B21, B23 or B24 as notified on the medical death certificate (DO-SIM) as the underlying cause of death.	**B20-Human immunodeficiency virus [HIV] disease resulting in infectious and parasitic diseases**<br>B20.0-HIV disease resulting in mycobacterial infections<br>B20.1-HIV disease resulting in other bacterial infections<br>B20.2-HIV disease resulting in cytomegalic disease<br>B20.3-HIV disease resulting in other viral infections<br>B20.4-HIV disease resulting in candidiasis<br>B20.5-HIV disease resulting in other mycoses<br>B20.6-HIV disease resulting in Pneumocystis jirovecii pneumonia<br>B20.7-HIV disease resulting in multiple infections<br>B20.8-HIV disease resulting in other infectious and parasitic diseases<br>B20.9-HIV disease resulting in unspecified infectious or parasitic disease<br>**B21-Human immunodeficiency virus [HIV] disease resulting in malignant neoplasms**<br>B21.0-HIV disease resulting in Kaposi's sarcoma<br>B21.1-HIV disease resulting in Burkitt's lymphoma<br>B21.2-HIV disease resulting in other types of non-Hodgkin's lymphoma<br>B21.3-HIV disease resulting in other malignant neoplasms of the lymphatic, hematopoietic and related tissues<br>B21.7-HIV disease resulting in multiple malignant neoplasms<br>B21.8-HIV disease resulting in other malignant neoplasms<br>B21.9-HIV disease resulting in unspecified malignant neoplasm<br>**B22-Human immunodeficiency virus [HIV] disease resulting in other specified diseases**<br>B22.0-HIV disease resulting in encephalopathy<br>B22.1-HIV disease resulting in lymphatic interstitial pneumonitis<br>B22.2-HIV disease resulting in emaciation syndrome<br>B22.7-HIV disease resulting in multiple illnesses classified elsewhere<br>**B23-Human immunodeficiency virus [HIV] disease resulting in other diseases**<br>B23.0-Acute HIV infection syndrome<br>B23.1-HIV disease resulting in generalized (persistent) lymphadenopathy<br>B23.2-HIV disease resulting in hematological and immunological abnormalities not elsewhere classified

		B23.8-HIV disease resulting in other specified conditions<br>**B24-Human immunodeficiency virus [HIV] disease not elsewhere specified**

Notes: [a] Laboratory evidence of HIV infection = two reactive screening tests (with different antigens or methodological principles); or one reactive confirmatory test.

From the identification of AIDS cases and deaths it was possible to calculate the AIDS incidence, mortality, and case-fatality rates. All AIDS outcomes calculated are rates, that is, we use the time of follow-ups of individuals on exposure (person-time) as the denominator. We used time in years for the estimates, as presented in Chart S2.

Chart S2. Formulas used to calculate AIDS incidence, mortality and case-fatality rates

AIDS outcome	Numerator	Denominator	Basis of 10
**Incidence rate**	New AIDS cases (Chart S1)	Person-years considering the follow-up time of individuals from the date of entry into the cohort until the date of AIDS diagnosis, death, or end of the cohort (12/31/2015).	100,000 py
**Mortality rate**	AIDS-related deaths (Chart S1)	Person-years considering the follow-up time of individuals from the date of entry into the cohort until the date of death from AIDS, death from other causes, or end of the cohort (12/31/2015).	100,000 py
**Case-Fatality rate**	AIDS-related deaths (Chart S1)	Person-years considering the follow-up time of individuals from the date of AIDS diagnosis until the date of death from AIDS, death from other causes, or end of cohort (12/31/2015).	100 py

Abbreviation: person-year (py)

For the calculations of incidence-rate ratios (IRRs), or just rate ratios (RR), Poisson regression models use the same rates (considering follow-up time), which are estimated by comparison groups and the rates between them are calculated, always considering the reference group (lowest exposure).

When running Poisson regression models in stata,[9] to find the probability of *k* events in an exposure of size *E*, we divide *E* into n subintervals *E1*, *E2*, . . . , *En*, and approximate the answer as the binomial probability of observing *k* successes in *n* trials. If we let $n \rightarrow \infty$, we obtain the Poisson distribution.

In the Poisson regression model, the incidence rate (or mortality or case-fatality-rates) for the *j*th observation is assumed to be given by:

$$r_j = e^{\beta 0+\beta 1x1,j+\cdots+\beta kxk,j}$$

If E_j is the exposure, the expected number of events, C_j, will be:

$$Cj = E_j e^{\beta 0+\beta 1x1,j+\cdots+\beta kxk,j} = e^{\ln(Ej)+\beta 0+\beta 1x1,j+\cdots+\beta kxk,j}$$

Without including the command options exposure () or offset (), *Ej* is assumed to be 1 (exposure is unknown). Comparing rates is most easily done by calculating IRRs.

For example, the relative incidence rate of AIDS cases for skin color white relative to skin color Black, we want to hold all the x's in the model constant except one, say, the *i*th. The IRR for a one-unit change in x_i is:

$$e^{\ln(E)+\beta 1x1+\cdots+\beta i\,(xi+1)+\cdots+\beta kxk} / e^{\ln(E)+\beta 1x1+\cdots+\beta ixi+\cdots+\beta kxk} = e^{\beta i}$$

More generally, the IRR for a Δ_{xi} change in x_i is e $\beta_i \Delta x_i$.

3. Sensitivity Analyses

Sensitivity analyses were performed to confirm the Poisson regression model as adequate for the analyses of the associations of social determinants of health with AIDS outcomes. To this end, alternative regression models were conducted with estimation of logistic, survival, negative binomial and zero-inflated regression models were run using the same structure.

It can be observed that the measures of associations and their respective 95% confidence intervals (95% CI) estimated by the different statistical models (Table S1, S2 and S3) are very similar for all outcomes assessed. We only observe an instability in the association measures between year of cohort entry in the AIDS incidence (Table S1) and mortality (Table S2) models. Confirming that the use of the Poisson regression models presented in the main article are adequate for the data.

Table S1. Sensitivity Analyses of model AIDS incidence rate: alternative regression models (N = 22,468,882).

	**Poisson regressions**	**Logistic regressions**[a]	**Zero-inflated Poisson regressions** (Zero obs = 22,447,471)	**Negative binomial regressions**	**Survival model**
	**RR (95% CI)**	**RR (95% CI)**	**RR (95% CI)**	**RR (95% CI)**	**HR (95% CI)**
**BLOCK 1 – GEOGRAPHIC FACTORS**					
**Region of residence**					
North	1.00	1.00	1.00	1.00	1.00
Northeast	1.22 (1.11-1.35)	1.24 (1.12-1.36)	1.25 (1.13-1.38)	1.26 (1.13-1.40)	1.22 (1.11-1.35)
Southeast	1.26 (1.15-1.39)	1.27 (1.16-1.39)	1.28 (1.16-1.41)	1.29 (1.17-1.43)	1.26 (1.15-1.39)
South	1.48 (1.35-1.63)	1.48 (1.35-1.63)	1.52 (1.37-1.68)	1.54 (1.38-1.72)	1.49 (1.35-1.63)
Mid-West	1.28 (1.13-1.45)	1.27 (1.13-1.44)	1.31 (1.15-1.48)	1.32 (1.16-1.51)	1.28 (1.13-1.44)
**Area of residence**					
Rural	1.00	1.00	1.00	1.00	1.00
Urbana	2.17 (2.02-2.33)	2.16 (2.01-2.33)	2.16 (2.01-2.33)	2.18 (2.03-2.33)	2.16 (2.02-2.33)
**BLOCK 2 – SOCIOECONOMIC STATUS**					
**Education**					
Illiterate/Never went to school	1.46 (1.26-1.68)	1.46 (1.27-1.69)	1.47 (1.27-1.71)	1.49 (1.28-1.73)	1.46 (1.26-1.68)
Literate/pre-school	1.10 (0.89-1.36)	1.10 (0.89-1.36)	1.11 (0.89-1.38)	1.11 (0.89-1.39)	1.10 (0.88-1.36)
Elementary school	1.43 (1.25-1.65)	1.44 (1.25-1.65)	1.45 (1.25-1.67)	1.46 (1.26-1.68)	1.43 (1.25-1.65)
High school	0.97 (0.85-1.09)	0.97 (0.85-1.09)	0.96 (0.85-1.10)	0.96 (0.84-1.10)	0.96 (0.85-1.10)
Higher education	1.00	1.00	1.00	1.00	1.00
**Race/skin color**					
White/Asian	1.00	1.00	1.00	1.00	1.00
Pardo	1.17 (1.12-1.22)	1.18 (1.13-1.23)	1.17 (1.12-1.23)	1.18 (1.12-1.23)	1.17 (1.12-1.22)
Black	1.53 (1.45-1.61)	1.53 (1.46-1.62)	1.55 (1.47-1.63)	1.57 (1.49-1.66)	1.53 (1.45-1.61)
Indigenous	1.12 (0.87-1.43)	1.16 (0.91-1.48)	1.12 (0.87-1.46)	1.13 (0.86-1.47)	1.12 (0.87-1.43)
**Wealth levels**					
Level 1 (Higher wealth)	1.00	1.00	1.00	1.00	1.00
Level 2	1.07 (1.01-1.14)	1.06 (0.99-1.13)	1.08 (1.01-1.15)	1.08 (1.01-1.16)	1.07 (1.01-1.14)
Level 3	1.27 (1.17-1.37)	1.25 (1.16-1.36)	1.29 (1.18-1.40)	1.30 (1.19-1.41)	1.27 (1.17-1.37)
Level 4	1.32 (1.23-1.43)	1.30 (1.20-1.41)	1.34 (1.24-1.45)	1.36 (1.25-1.48)	1.32 (1.23-1.43)
Level 5 (Lower wealth)	1.55 (1.43-1.68)	1.46 (1.35-1.58)	1.59 (1.46-1.73)	1.62 (1.48-1.77)	1.55 (1.43-1.68)
**Receipt time of Bolsa Família**					

Does not receive	1.00	1.00	1.00	1.00	1.00
Less than 2 years	1.08 (1.03-1.14)	1.09 (1.03-1.15)	1.08 (1.02-1.14)	1.08 (1.03-1.14)	1.08 (1.03-1.14)
Between 2 and 5 years	1.05 (1.00-1.10)	1.05 (1.00-1.11)	1.05 (0.99-1.10)	1.05 (0.99-1.10)	1.05 (1.00-1.10)
Between 5 and 10 years	0.86 (0.81-0.91)	0.86 (0.82-0.91)	0.85 (0.81-0.91)	0.85 (0.80-0.90)	0.86 (0.81-0.91)
More than 10 years	0.70 (0.65-0.75)	0.69 (0.64-0.73)	0.69 (0.64-0.74)	0.68 (0.63-0.74)	0.70 (0.65-0.75)
**BLOCK 3 – HOUSEHOLD CONDITIONS**					
**Household material**					
Brick	1.00	1.00	1.00	1.00	1.00
Wood	1.21 (1.15-1.28)	1.22 (1.15-1.28)	1.23 (1.16-1.31)	1.24 (1.17-1.33)	1.21 (1.15-1.28)
Mud and others	1.07 (1.00-1.14)	1.05 (0.99-1.12)	1.07 (1.00-1.14)	1.07 (1.00-1.14)	1.07 (1.00-1.14)
**Electrical power**					
Yes	1.00	1.00	1.00	1.00	1.00
No	1.15 (1.09-1.21)	1.15 (1.09-1.21)	1.15 (1.09-1.22)	1.16 (1.09-1.22)	1.15 (1.09-1.21)
**Household density (**residents/room)					
up to 2	1.00	1.00	1.00	1.00	1.00
more than 2	1.15 (1.09-1.22)	1.15 (1.09-1.22)	1.15 (1.08-1.22)	1.15 (1.08-1.22)	1.15 (1.09-1.22)
**ADJUSTMENT VARIABLES**					
**Gender**					
Female	1.00	1.00	1.00	1.00	1.00
Male	1.20 (1.12-1.278)	1.20 (1.12-1.28)	1.22 (1.14-1.30)	1.23 (1.14-1.33)	1.20 (1.12-1.28)
**Age**					
Adolescents (13-17 years old)	1.00	1.00	1.00	1.00	1.00
Young (17-23.9 years old)	2.92 (2.70-3.16)	2.98 (2.76-3.22)	3.01 (2.77-3.28)	3.08 (2.81-3.38)	2.92 (2.70-3.16)
24-34.9 years old	4.86 (4.49-5.25)	5.08 (4.69-5.50)	5.06 (4.68-5.47)	5.23 (4.81-5.69)	4.85 (4.49-5.25)
35-44.9 years	5.26 (4.87-5.68)	5.48 (5.07-5.93)	5.50 (5.10-5.94)	5.72 (5.24-6.25)	5.25 (4.86-5.68)
45-54.9 years	3.97 (3.61-4.36)	4.18 (3.79-4.60)	4.12 (3.74-4.55)	4.25 (3.83-4.73)	3.96 (3.60-4.36)
55-64.9 years	2.14 (1.90-2.40)	2.29 (2.04-2.58)	2.18 (1.94-2.45)	2.22 (1.97-2.49)	2.13 (1.90-2.40)
Elderly (65 years or older)	0.85 (0.74-.97)	0.94 (0.82-1.08)	0.86 (0.75-0.98)	.86 (0.75-0.99)	0.84 (0.74-0.97)
**Year of admission to the cohort**					
2007	1.00	1.00	1.00	1.00	1.00
2008	1.13 (1.08-1.18)	3.01 (2.84-3.19)	1.13 (1.07-1.18)	1.13 (1.07-1.18)	1.12 (1.07-1.17)
2009	1.10 (1.04-1.17)	7.17 (6.75-7.61)	1.09 (1.03-1.16)	1.08 (1.01-1.15)	1.10 (1.03-1.16)
2010	1.07 (1.01-1.13)	13.35 (12.60-14.14)	1.06 (0.99-1.12)	1.05 (0.97-1.12)	1.06 (0.99-1.13)
2011	1.12 (1.04-1.21)	35.74 (32.99-38.71)	1.10 (1.02-1.19)	1.09 (1.00-1.19)	1.10 (1.02-1.20)
2012	1.09 (1.02-1.17)	59.26 (55.17-63.65)	1.07 (0.99-1.16)	1.05 (0.97-1.15)	1.07 (0.98-1.16)
2013	0.99 (0.91-1.09)	97.74 (90.72-105.31)	0.97 (0.88-1.06)	0.95 (0.86-1.04)	0.96 (0.87-1.05)
2014	1.00 (0.92-1.09)	140.61 (130.81-151.14)	0.96 (0.88-1.05)	0.94 (0.85-1.03)	0.93 (0.84-1.02)
2015	0.93 (0.79-1.09)	128.29 (113.55-144.95)	0.89 (0.76-1.04)	0.86 (0.72-1.01)	0.83 (0.70-0.97)
**Municipal HIV/AIDS endemicity and surveillance in the period (2007-2015)**					
Average AIDS morbidity rate	1.03 (1.03-1.04)	1.03 (1.03 -1.04)	1.03 (1.03-1.04)	1.03 (1.03-1.04)	1.03 (1.03-1.03)

Notes: [a]The logistic regression model included of follow-up time as a compensation term (offset) and cluster-robust standard errors (municipality of residence), in addition to point estimates and delta method standard errors for converting OR to RR, calculated using the logit post-estimation command in STATA (adjrr)[9].

Abbreviations: RR: Rate Ratios; CI: confidence interval; HR: Hazard Ratio

Table S2. Sensitivity Analyses of model AIDS mortality rate: alternative regression models (N=22,468,903).

	**Poisson Regressions**	**Logistic Regressions[a]**	**Zero-Inflated Poisson Regressions** (Zero obs=22,461,378)	**Negative Binomial Regressions**	**Survival model**
	**RR (95% CI)**	**RR (95% CI)**	**RR (95% CI)**	**RR (95% CI)**	**HR (95% CI)**
**BLOCK 1 – GEOGRAPHIC FACTORS**					
**Region of residence**					
North	1.00	1.00	1.00	1.00	1.00
Northeast	1.16 (1.03-1.31)	1.17 (1.04-1.33)	1.17 (1.03-1.33)	1.17 (1.03-1.33)	1.16 (1.03-1.32)
Southeast	1.46 (1.20-1.78)	1.47 (1.21-1.77)	1.46 (1.20-1.78)	1.46 (1.20-1.78)	1.46 (1.20-1.78)
South	1.36 (1.14-1.63)	1.36 (1.13-1.63)	1.38 (1.15-1.65)	1.38 (1.15-1.66)	1.36 (1.14-1.63)
Mid-West	1.21 (1.03-1.42)	1.20 (1.02-1.41)	1.22 (1.03-1.43)	1.22 (1.03-1.44)	1.21 (1.03-1.42)
**Area of residence**					
Rural	1.00	1.00	1.00	1.00	1.00
Urbana	2.35 (2.11-2.62)	2.36 (2.11-2.63)	2.35 (2.10-2.63)	2.35 (2.10-2.63)	2.35 (2.11-2.62)
**BLOCK 2 – SOCIOECONOMIC STATUS**					
**Education**					
Illiterate/Never went to school	2.76 (1.99-3.82)	2.77 (2.00-3.83)	2.79 (2.01-3.88)	2.81 (2.02-3.90)	2.76 (1.99-3.82)
Literate/pre-school	1.46 (0.95-2.25)	1.46 (0.95-2.24)	1.47 (0.95-2.28)	1.48 (0.96-2.29)	1.46 (0.95-2.24)
Elementary school	2.50 (1.83-3.41)	2.50 (1.83-3.40)	2.53 (1.85-3.45)	2.54 (1.85-3.48)	2.50 (1.83-3.41)
High school	1.23 (0.88-1.71)	1.23 (0.89-1.71)	1.23 (0.88-1.72)	1.23 (0.88-1.72)	1.23 (0.88-1.71)
Higher education	1.00	1.00	1.00	1.00	1.00
**Race/skin color**					
White/Asian	1.00	1.00	1.00	1.00	1.00
Pardo	1.19 (1.12-1.27)	1.20 (1.13-1.28)	1.20 (1.12-1.29)	1.20 (1.12-1.29)	1.19 (1.12-1.28)
Black	1.69 (1.57-1.83)	1.70 (1.57-1.84)	1.72 (1.58-1.88)	1.73 (1.58-1.90)	1.70 (1.57-1.83)
Indigenous	1.20 (0.82-1.75)	1.24 (0.85-1.80)	1.19 (0.80-1.76)	1.19 (0.81-1.77)	1.20 (0.82-1.75)
**Wealth levels**					
Level 1 (Higher wealth)	1.00	1.00	1.00	1.00	1.00
Level 2	1.17 (1.04-1.32)	1.16 (1.03-1.31)	1.17 (1.04-1.33)	1.17 (1.04-1.33)	1.17 (1.04-1.326)
Level 3	1.42 (1.21-1.66)	1.40 (1.19-1.64)	1.43 (1.21-1.68)	1.43 (1.21-1.68)	1.41 (1.21-1.66)
Level 4	1.58 (1.36-1.82)	1.55 (1.34-1.80)	1.59 (1.37-1.84)	1.59 (1.37-1.85)	1.57 (1.36-1.82)
Level 5 (Lower wealth)	1.99 (1.70-2.34)	1.88 (1.61-2.21)	2.02 (1.72-2.37)	2.03 (1.72-2.39)	1.99 (1.70-2.33)
**Receipt time of *Bolsa Família***					
Does not receive	1.00	1.00	1.00	1.00	1.00
Less than 2 years	1.18 (1.08-1.29)	1.19 (1.09-1.29)	1.18 (1.08-1.29)	1.19 (1.09-1.29)	1.18 (1.08-1.29)
Between 2 and 5 years	1.05 (0.96-1.14)	1.05 (0.97-1.14)	1.05 (0.96-1.14)	1.04 (0.96-1.14)	1.05 (0.96-1.14)
Between 5 and 10 years	0.66 (0.61-0.72)	0.66 (0.61-0.72)	0.65 (0.60-0.71)	0.65 (0.60-0.71)	0.66 (0.61-0.72)
More than 10 years	0.47 (0.42-0.52)	0.45 (0.41-0.51)	0.46 (0.41-0.51)	0.45 (0.40-0.51)	0.46 (0.42-0.52)
**BLOCK 3 – HOUSEHOLD CONDITIONS**					
**Household material**					
Brick	1.00	1.00	1.00	1.00	1.00
Wood	1.31 (1.19-1.43)	1.31 (1.20-1.43)	1.32 (1.20-1.45)	1.32 (1.20-1.45)	1.31 (1.20-1.43)

Mud and others	1.10 (0.99-1.21)	1.08 (0.98-1.20)	1.10 (0.99-1.21)	1.10 (0.99-1.21)	1.10 (0.99-1.21)
**Electrical power**					
Yes	1.00	1.00	1.00	1.00	1.00
No	1.24 (1.16-1.33)	1.25 (1.16-1.34)	1.25 (1.16-1.34)	1.25 (1.16-1.34)	1.24 (1.16-1.33)
**Household density (**residents/room)					
up to 2	1.00	1.00	1.00	1.00	1.00
more than 2	1.23 (1.12-1.35)	1.23 (1.12-1.35)	1.24 (1.12-1.37)	1.24 (1.12-1.37)	1.23 (1.12-1.35)
**ADJUSTMENT VARIABLES**					
**Gender**					
Female	1.00	1.00	1.00	1.00	1.00
Male	1.27 (1.20-1.34)	1.26 (1.19-1.33)	1.28 (1.21-1.35)	1.28 (1.21-1.36)	1.27 (1.20-1.34)
**Age**					
Adolescents (13-17 years old)	1.00	1.00	1.00	1.00	1.00
Young (17-23.9 years old)	3.84 (3.20-4.61)	3.91 (3.25-4.69)	3.90 (3.23-4.70)	3.92 (3.23-4.75)	3.85 (3.20-4.62)
24-34.9 years old	9.30 (7.93-10.90)	9.73 (8.30-11.41)	9.54 (8.11-1.12)	9.63 (8.13-11.40)	9.32 (7.95-1.09)
35-44.9 years	12.31 (10.30-14.70)	12.83 (10.73-15.35)	12.69 (10.63-15.15)	12.85 (10.70-15.43)	12.33 (10.33-14.73)
45-54.9 years	9.53 (8.02-11.32)	10.06 (8.47-11.95)	9.80 (8.24-1.16)	9.90 (8.28-11.83)	9.56 (8.05-1.13)
55-64.9 years	5.35 (4.43-6.47)	5.78 (4.77-6.99)	5.44 (4.50-6.57)	5.48 (4.53-6.62)	5.38 (4.45-6.50)
Elderly (65 years or older)	2.32 (1.83-2.93)	2.60 (2.05-3.29)	2.35 (1.86-2.97)	2.36 (1.87-2.97)	2.33 (1.85-2.95)
**Year of admission to the cohort**					
2007	1.00	1.00	1.00	1.00	1.00
2008	1.17 (1.08-1.27)	3.14 (2.89-3.41)	1.17 (1.08-1.27)	1.17 (1.08-1.27)	1.21 (1.12-1.32)
2009	1.10 (1.01-1.20)	7.22 (6.63-7.86)	1.09 (0.99-1.19)	1.09 (0.99-1.19)	1.16 (1.06-1.27)
2010	0.96 (0.88-1.05)	12.25 (11.17-13.44)	0.95 (0.86-1.04)	0.94 (0.85-1.04)	1.02 (0.93-1.13)
2011	1.11 (0.97-1.26)	37.31 (32.84-42.39)	1.10 (0.96-1.25)	1.09 (0.96-1.24)	1.20 (1.05-1.38)
2012	1.04 (0.92-1.17)	60.92 (54.69-67.87)	1.02 (0.91-1.15)	1.02 (0.90-1.15)	1.15 (1.02-1.31)
2013	0.89 (0.77-1.02)	99.11 (87.69-112.01)	0.87 (0.76-1.00)	0.86 (0.75-0.99)	1.00 (0.86-1.16)
2014	0.89 (0.74-1.08)	149.64 (127.64-175.43)	0.87 (0.73-1.05)	0.87 (0.72-1.04)	1.03 (0.85-1.26)
2015	1.01 (0.77-1.32)	159.43 (128.70-197.51)	0.98 (0.76-1.28)	0.97 (0.75-1.26)	1.23 (0.93-1.63)
**Municipal HIV/AIDS endemicity and surveillance in the period (2007-2015)**					
Average AIDS mortality rate	1.10 (1.081.12)	1.10 (1.08- 1.13)	1.11 (1.08-1.14)	1.11 (1.08-1.14)	1.10 (1.08-1.12)

Notes: [a]The logistic regression model included of follow-up time as a compensation term (offset) and cluster-robust standard errors (municipality of residence), in addition to point estimates and delta method standard errors for converting OR to RR, calculated using the logit post-estimation command in STATA (adjrr)[9].

Abbreviations: RR: Rate Ratio; CI: confidence interval; HR: Hazard Ratio

Table S3. Sensitivity Analyses of model AIDS case-fatality rate over the study period: alternative regression models (N= 11,815).

	Poisson regressions	Logistic regressions[a]	zero-inflated Poisson regressions (Zero obs==9,932)	negative binomial regressions	Survival model
	RR (95% CI)	RR (95% CI)	RR (95% CI)	RR (95% CI)	HR (95% CI)
**BLOCK 1 – GEOGRAPHIC FACTORS**					
**Region of residence**					
North	1.00	1.00	1.00	1.00	1.00
Northeast	0.96 (0.76-1.21)	0.89 (0.71-1.10)	0.75 (0.50-1.12)	0.68 (0.39-1.17)	0.95 (0.78-1.17)
Southeast	0.81 (0.64-1.03)	0.88 (0.70-1.10)	0.48 (0.32-0.71)	0.48 (0.29-0.81)	0.86 (0.70-1.07)
South	0.92 (0.71-1.18)	0.91 (0.72-1.14)	0.60 (0.38-0.94)	0.54 (0.31-0.95)	0.93 (0.74-1.17)
Mid-West	1.09 (0.82-1.45)	1.05 (0.80-1.37)	1.11 (0.64-1.91)	1.14 (0.62-2.08)	1.10 (0.86-1.42)
**BLOCK 2 – SOCIOECONOMIC STATUS**					
**Education**					
Illiterate/Never went to school	1.86 (1.02-3.41)	1.87 (1.09-3.22)	2.99 (1.22-7.35)	2.80 (0.65-1.22)	1.85 (1.03-3.30)
Literate/pre-school	1.23 (0.52-2.90)	0.94 (0.43-2.11)	22.24 (4.57-108.29)	3.55 (0.28-4.47)	1.23 (0.54-2.79)
Elementary school	1.73 (0.97-3.06)	1.69 (1.01-2.83)	2.97 (1.26-6.98)	2.68 (0.69-1.04)	1.71 (0.98-2.99)
High school	1.35 (0.75-2.42)	1.33 (0.78-2.27)	1.85 (0.76-4.52)	1.57 (0.39-6.31)	1.33 (0.75-2.36)
Higher education	1.00	1.00	1.00	1.00	1.00
**Race/skin color**					
White/Asian	1.00	1.00	1.00	1.00	1.00
Pardo	1.09 (0.96-1.24)	1.04 (0.93-1.16)	0.93 (0.73-1.18)	0.98 (0.71-1.36)	1.06 (0.94-1.20)
Black	1.14 (1.01-1.30)	1.10 (0.97-1.25)	0.82 (0.62-1.07)	1.11 (0.76-1.62)	1.13 (1.01-1.27)
Indigenous	0.58 (0.22-1.55)	0.84 (0.29-2.47)	0.89 (0.22-3.64)	0.85 (0.09-8.02)	0.64 (0.26-1.56)
**Wealth levels**					
Level 1 (Higher wealth)	1.00	1.00	1.00	1.00	1.00
Level 2	1.18 (0.90-1.53)	1.08 (0.88-1.33)	1.21 (0.72-2.05)	1.61 (0.94-2.77)	1.17 (0.92-1.50)
Level 3	1.07 (0.79-1.45)	1.03 (0.81-1.32)	1.46 (0.78-2.74)	1.75 (0.94-3.28)	1.09 (0.83-1.42)
Level 4	1.01 (0.78-1.32)	1.00 (0.80-1.24)	1.03 (0.62-1.72)	1.07 (0.58-1.98)	1.03 (0.81-1.31)
Level 5 (Lower wealth)	1.09 (0.81-1.45)	1.07 (0.84-1.35)	1.01 (0.58-1.76)	1.24 (0.64-2.40)	1.10 (0.85-1.44)
**Receipt time of *Bolsa Família***					
Does not receive	1.00	1.00	1.00	1.00	1.00
Less than 2 years	1.23 (1.02-1.50)	1.20 (1.02-1.42)	1.30 (0.89-1.89)	1.49 (0.94-2.36)	1.19 (1.00-1.42)
Between 2 and 5 years	1.02 (0.87-1.21)	1.01 (0.87-1.17)	1.05 (0.78-1.43)	0.92 (0.62-1.37)	1.02 (0.87-1.19)
Between 5 and 10 years	0.77 (0.65-0.91)	0.73 (0.63-0.85)	0.72 (0.52-1.00)	0.48 (0.31-0.75)	0.78 (0.66-0.91)
More than 10 years	0.57 (0.46-0.72)	0.52 (0.42-0.65)	0.46 (0.30-0.69)	0.30 (0.17-0.53)	0.58 (0.47-0.72)
**BLOCK 3 – HOUSEHOLD CONDITIONS**					
**Household density** (residents/room)					
up to 2	1.00	1.00	1.00	1.00	1.00
more than 2	1.35 (1.12-1.63)	1.23 (1.02-1.50)	1.22 (0.93-1.62)	1.26 (0.80-1.99)	1.30 (1.09-1.56)
**Sewage system**					
Sewage network	1.00	1.00	1.00	1.00	1.00
Septic tank	1.05 (0.89-1.24)	1.07 (0.92-1.26)	0.83 (0.60-1.13)	1.03 (0.69-1.54)	1.04 (0.89-1.22)

Others	1.17 (1.03-1.33)	1.13 (0.99-1.27)	1.11 (0.87-1.42)	1.33 (0.96-1.84)	1.14 (1.02-1.29)
**BLOCK 4 – HEALTH AND BEHAVIORAL ASPECTS**					
**Treatment**					
Yes	1.00	1.00	1.00	1.00	1.00
No	2.58 (2.22-3.00)	2.84 (2.57-3.15)	3.46 (2.62-4.57)	12.19 (9.17-16.20)	2.36 (2.11-2.65)
**Exposure Category**					
Homosexual	1.00	1.00	1.00	1.00	1.00
Bisexual	1.06 (0.79-1.43)	1.15 (0.86-1.52)	1.19 (0.70-2.02)	1.01 (0.52-1.94)	0.65 (0.54-0.79)
Heterosexual	1.56 (1.29-1.90)	1.61 (1.33-1.94)	1.64 (1.16-2.31)	1.98 (1.28-3.08)	0.73 (0.57-0.92)
People who inject drugs	3.05 (2.36-3.94)	3.16 (2.46-4.06)	2.39 (1.51-3.78)	8.92 (4.39-18.12)	1.89 (1.59-2.24)
**ADJUSTMENT VARIABLES**					
**Gender**					
Female	1.00	1.00	1.00	1.00	1.00
Male	1.36 (1.21-1.54)	1.26 (1.13-1.41)	1.40 (1.14-1.72)	1.49 (1.14-1.95)	1.31 (1.17-1.47)
**Age**					
Adolescents (13-17 years old)	1.00	1.00	1.00	1.00	1.00
Young (17-23.9 years old)	0.88 (0.66-1.18)	1.46 (1.08-1.97)	1.10 (0.70-1.75)	1.69 (0.90-3.21)	1.06 (0.80-1.41)
24-34.9 years old	1.11 (0.82-1.50)	2.17 (1.56-3.01)	1.50 (1.00-2.23)	2.91 (1.60-5.29)	1.42 (1.06-1.91)
35-44.9 years	1.24 (0.92-1.67)	2.67 (1.95-3.65)	1.78 (1.15-2.74)	4.90 (2.65-9.07)	1.63 (1.22-2.16)
45-54.9 years	1.39 (1.01-1.93)	2.97 (2.12-4.17)	2.54 (1.57-4.12)	6.11 (3.10-12.06)	1.79 (1.30-2.44)
55-64.9 years	2.46 (1.75-3.46)	4.40 (3.14-6.17)	3.68 (2.25-6.04)	14.32 (6.79-30.20)	2.88 (2.09-3.97)
Elderly (65 years or older)	3.25 (2.05-5.15)	5.05 (3.31-7.72)	2.77 (1.42-5.41)	15.36 (5.78-40.80)	3.51 (2.32-5.32)
**Year of admission to the cohort**					
2007	1.00	1.00	1.00	1.00	1.00
2008	1.11 (0.95-1.29)	2.06 (1.81-2.34)	1.01 (0.76-1.32)	1.22 (0.83-1.80)	1.05 (0.91-1.20)
2009	1.24 (1.07-1.44)	3.29 (2.91-3.71)	1.06 (0.77-1.46)	0.95 (0.62-1.46)	1.07 (0.94-1.23)
2010	1.04 (0.85-1.27)	3.94 (3.46-4.48)	0.98 (0.65-1.47)	0.84 (0.51-1.38)	0.86 (0.71-1.03)
2011	1.13 (0.86-1.48)	5.42 (4.77-6.16)	0.94 (0.58-1.53)	0.62 (0.32-1.20)	0.84 (0.66-1.08)
2012	1.28 (1.01-1.64)	6.46 (5.76-7.26)	1.19 (0.71-2.00)	0.51 (0.26-1.00)	0.86 (0.68-1.08)
2013	1.35 (0.93-1.97)	7.48 (6.61-8.46)	1.04 (0.56-1.92)	0.34 (0.14-0.78)	0.72 (0.51-1.02)
2014	2.74 (1.96-3.81)	8.79 (7.86-9.83)	3.10 (1.26-7.60)	1.07 (0.44-2.58)	1.04 (0.76-1.43)
2015	4.59 (2.29-9.20)	9.26 (8.14-10.53)	5.45 (1.80-1.65)	0.93 (0.26-3.37)	1.08 (0.57-2.07)
**Municipal HIV/AIDS endemicity and surveillance in the period (2007-2015)**					
Average AIDS case-fatality rate	1.02 (1.01-1.02)	1.02 (1.01-1.03)	1.03 (1.02-1.04)	1.05 (1.03-1.07)	1.02 (1.01-1.02)

Notes: [a]The logistic regression model included of follow-up time as a compensation term (offset) and cluster-robust standard errors (municipality of residence), in addition to point estimates and delta method standard errors for converting OR to RR, calculated using the logit post-estimation command in STATA (adjrr)[9].

Abbreviations: RR: Rate Ratio; CI: confidence interval; HR: Hazard Ratio

Additional sensitivity analyses were performed to check the influence of municipal characteristics on the estimates, with the inclusion of aggregate variables at the municipal level to adjust for confounding: health infrastructure in the municipality of residence (coverage of primary health care -Family Health Strategy (FHS), number of specialized clinics per 1000 population, number of physicians per 1000 population, number of hospital beds per 1000 population, epidemiological situation regarding the outcome under analysis (average outcome rate in the municipality of residence) and socio-environmental conditions of the municipality (gini index, and extreme poverty and unemployment rates). The estimates of RR and CI did not change significantly (Tables S4, S5 and S6), opting to maintain the reduced model in the main analyses and thus avoid overfitting.
We also verified the socio-spatial influence of the adequacy of of health information (health surveillance) among Brazilian municipalities, with Poisson regressions models estimated only with the inclusion of individuals living in municipalities with adequate quality of vital information. The criterion of adequacy of vital information was used as the work of Andrade and Szwarcwald[10], which considers five indicators: general mortality coefficient standardized by age; ratio between live births informed and estimated; average relative deviation of the mortality coefficient; average relative deviation of the birth rate; percentage of deaths without definition of the basic cause of death. All analyses produce similar estimates (Tables S4, S5 and S6), showing that the limitations of health information in some Brazilian municipalities do not influence the findings, allowing causal inferences to be made for the entire country.

Table S4. Sensitivity Analyses of model AIDS incidence rate: Quality of Vital Information and aggregate municipal variables for adjustment

	**Poisson regressions**			
	**Model 1** (N = 22,468,882)	**Model 2** (N=18,087,608)	**Model 3** (N= 22,353,373)	**Model 4** (N= 18,049,737)
	**RR (95% CI)**	**RR (95% CI)**	**RR (95% CI)**	**RR (95% CI)**
**BLOCK 1 – GEOGRAPHIC FACTORS**				
**Region of residence**				
North	1.00	1.00	1.00	1.00
Northeast	1.22 (1.11-1.35)	1.28 (1.15-1.43)	1.19 (1.08-1.31)	1.24 (1.12-1.38)
Southeast	1.26 (1.15-1.39)	1.29 (1.16-1.44)	1.13 (1.00-1.28)	1.15 (1.00-1.32)
South	1.48 (1.35-1.63)	1.55 (1.40-1.72)	1.35 (1.20-1.52)	1.38 (1.22-1.58)
Mid-West	1.28 (1.13-1.45)	1.31 (1.13-1.51)	1.18 (1.03-1.35)	1.19 (1.01-1.39)
**Area of residence**				
Rural	1.00	1.00	1.00	1.00
Urbana	2.17 (2.02-2.33)	2.18 (2.01-2.37)	2.03 (1.90-2.16)	2.04 (1.89-2.20)
**BLOCK 2 – SOCIOECONOMIC STATUS**				
**Education**				
Higher education	1.00	1.00	1.00	1.00
High school	0.97 (0.85-1.09)	0.94 (0.83-1.08)	0.95 (0.84-1.08)	0.93 (0.82-1.06)
Elementary school	1.43 (1.25-1.65)	1.40 (1.21-1.62)	1.42 (1.23-1.62)	1.39 (1.20-1.60)
Literate/pre-school	1.10 (0.89-1.36)	1.10 (0.88-1.38)	1.10 (0.89-1.36)	1.10 (0.87-1.38)
Illiterate/Never went to school	1.46 (1.26-1.68)	1.42 (1.21-1.65)	1.45 (1.26-1.68)	1.42 (1.22-1.65)
**Race/skin color**				
White/Asian	1.00	1.00	1.00	1.00
Pardo	1.17 (1.12-1.22)	1.17 (1.11-1.22)	1.17 (1.12-1.23)	1.17 (1.12-1.23)
Black	1.53 (1.45-1.61)	1.54 (1.46-1.63)	1.53 (1.45-1.62)	1.55 (1.46-1.64)
Indigenous	1.12 (0.87-1.43)	1.05 (0.78-1.40)	1.20 (0.94-1.52)	1.12 (0.85-1.47)

**Wealth levels**				
Level 1 (Higher wealth)	1.00	1.00	1.00	1.00
Level 2	1.07 (1.01-1.14)	1.06 (0.99-1.13)	1.07 (1.01-1.15)	1.06 (0.99-1.13)
Level 3	1.27 (1.17-1.37)	1.27 (1.17-1.38)	1.27 (1.17-1.38)	1.28 (1.17-1.39)
Level 4	1.32 (1.23-1.43)	1.30 (1.20-1.40)	1.33 (1.23-1.43)	1.30 (1.20-1.40)
Level 5 (Lower wealth)	1.55 (1.43-1.68)	1.54 (1.42-1.68)	1.57 (1.45-1.70)	1.56 (1.43-1.70)
**Receipt time of Bolsa Família**				
Does not receive	1.00	1.00	1.00	1.00
Less than 2 years	1.08 (1.03-1.14)	1.08 (1.02-1.14)	1.08 (1.03-1.14)	1.08 (1.02-1.14)
Between 2 and 5 years	1.05 (1.00-1.10)	1.05 (1.00-1.11)	1.05 (1.00-1.11)	1.05 (1.00-1.11)
Between 5 and 10 years	0.86 (0.81-0.91)	0.86 (0.81-0.92)	0.87 (0.82-0.92)	0.87 (0.82-0.93)
More than 10 years	0.70 (0.65-0.75)	0.72 (0.67-0.78)	0.72 (0.67-0.77)	0.73 (0.68-0.79)
**BLOCK 3 – HOUSEHOLD CONDITIONS**				
**Household material**				
Brick	1.00	1.00	1.00	1.00
Wood	1.21 (1.15-1.28)	1.23 (1.16-1.31)	1.23 (1.16-1.30)	1.25 (1.17-1.32)
Mud and others	1.07 (1.00-1.14)	1.08 (1.00-1.16)	1.13 (1.06-1.21)	1.14 (1.06-1.23)
**Electrical power**				
Yes	1.00	1.00	1.00	1.00
No	1.15 (1.09-1.21)	1.15 (1.09-1.22)	1.16 (1.10-1.22)	1.17 (1.10-1.23)
**Household density (**residents/room)				
up to 2	1.00	1.00	1.00	1.00
more than 2	1.15 (1.09-1.22)	1.14 (1.07-1.21)	1.14 (1.07-1.20)	1.13 (1.06-1.20)
**ADJUSTMENT VARIABLES**				
**Gender**				
Female	1.00	1.00	1.00	1.00
Male	1.20 (1.12-1.278)	1.23 (1.15-1.32)	1.20 (1.13-1.28)	1.24 (1.16-1.32)
**Age**				
Adolescents (13-17 years old)	1.00	1.00	1.00	1.00
Young (17-23.9 years old)	2.92 (2.70-3.16)	2.92 (2.69-3.18)	2.93 (2.71-3.17)	2.93 (2.69-3.19)
24-34.9 years old	4.86 (4.49-5.25)	4.85 (4.46-5.28)	4.84 (4.47-5.23)	4.84 (4.45-5.26)
35-44.9 years	5.26 (4.87-5.68)	5.22 (4.80-5.66)	5.22 (4.84-5.64)	5.20 (4.79-5.64)
45-54.9 years	3.97 (3.61-4.36)	4.03 (3.64-4.46)	3.96 (3.60-4.35)	4.03 (3.64-4.46)
55-64.9 years	2.14 (1.90-2.40)	2.16 (1.91-2.45)	2.13 (1.90-2.40)	2.16 (1.90-2.45)
Elderly (65 years or older)	0.85 (0.74-0.97)	0.87 (0.76-1.01)	0.85 (0.75-0.98)	0.88 (0.76-1.01)
**Year of admission to the cohort**				
2007	1.00	1.00	1.00	1.00
2008	1.13 (1.08-1.18)	1.13 (1.07-1.18)	1.13 (1.08-1.18)	1.12 (1.07-1.18)
2009	1.10 (1.04-1.17)	1.10 (1.04-1.17)	1.10 (1.04-1.16)	1.09 (1.03-1.16)
2010	1.07 (1.01-1.13)	1.08 (1.02-1.15)	1.07 (1.01-1.14)	1.08 (1.01-1.15)
2011	1.12 (1.04-1.21)	1.12 (1.03-1.21)	1.12 (1.04-1.22)	1.11 (1.02-1.21)
2012	1.09 (1.02-1.17)	1.09 (1.01-1.18)	1.10 (1.02-1.19)	1.08 (0.99-1.18)
2013	0.99 (0.91-1.09)	0.99 (0.90-1.08)	1.02 (0.93-1.11)	1.00 (0.90-1.10)

2014	1.00 (0.92-1.09)	0.99 (0.90-1.08)	1.02 (0.93-1.12)	0.99 (0.89-1.09)
2015	0.93 (0.79-1.09)	0.92 (0.78-1.09)	0.93 (0.79-1.10)	0.91 (0.77-1.08)
**Municipal aggregate variables**				
Municipal HIV/AIDS endemicity and surveillance in the period				
Average AIDS morbidity rate	1.03 (1.03-1.04)	1.03 (1.03-1.03)	1.03 (1.03-1.03)	1.03 (1.03-1.03)
Coverage of Family Health Strategy - FHS	-	-	0.95 (0.86-1.06)	0.94 (0.83-1.07)
Specialized clinics rate	-	-	1.29 (1.03-1.62)	1.26 (0.99-1.60)
Physicians per 1K	-	-	1.01 (0.97-1.05)	1.01 (0.98-1.05)
Hospital beds per 1K	-	-	0.99 (0.97-1.00)	0.98 (0.97-1.00)
Gini index	-	-	0.41 (0.22-0.77)	0.42 (0.21-0.87)
Extreme poverty rate	-	-	0.48 (0.30-0.77)	0.40 (0.23-0.68)
Unemployment rate	-	-	3.39 (1.57-7.34)	2.52 (1.01-6.33)

Model 1: Original model with all municipalities;
Model 2: Original model only with municipality with adequate Quality of Vital Information;
Model 3: Model with all municipalities and inclusion of municipal aggregate variables for adjustment;
Model 4: Model only with municipality with adequate Quality of Vital Information and inclusion of aggregate municipal variables for adjustment.
Abbreviations: RR: Rate Ratios; CI: confidence interval.

Table S5. Sensitivity Analyses of model AIDS mortality rate: Quality of Vital Information and aggregate municipal variables for adjustment

	Poisson regressions			
	**Model 1** (N =22,468,903)	**Model 2** (N=18,087,627)	**Model 3** (N= 22,353,394)	**Model 4** (N= 18,049,756)
	**RR (95% CI)**	**RR (95% CI)**	**RR (95% CI)**	**RR (95% CI)**
**BLOCK 1 – GEOGRAPHIC FACTORS**				
**Region of residence**				
North	1.00	1.00	1.00	1.00
Northeast	1.16 (1.03-1.31)	1.22 (1.07-1.40)	1.10 (0.96-1.26)	1.15 (1.00-1.33)
Southeast	1.46 (1.20-1.78)	1.50 (1.21-1.87)	1.31 (0.97-1.75)	1.32 (0.95-1.84)
South	1.36 (1.14-1.63)	1.42 (1.18-1.71)	1.29 (1.05-1.58)	1.29 (1.03-1.62)
Mid-West	1.21 (1.03-1.42)	1.20 (1.00-1.45)	1.12 (0.92-1.36)	1.08 (0.87-1.35)
**Area of residence**				
Rural	1.00	1.00	1.00	1.00
Urbana	2.35 (2.11-2.62)	2.35 (2.07-2.67)	2.15 (1.95-2.36)	2.15 (1.91-2.41)
**BLOCK 2 – SOCIOECONOMIC STATUS**				
**Education**				
Higher education	1.00	1.00	1.00	1.00
High school	1.23 (0.88-1.71)	1.26 (0.89-1.78)	1.21 (0.87-1.69)	1.24 (0.88-1.76)
Elementary school	2.50 (1.83-3.41)	2.49 (1.79-3.46)	2.46 (1.80-3.36)	2.46 (1.77-3.43)
Literate/pre-school	1.46 (0.95-2.25)	1.42 (0.89-2.26)	1.46 (0.95-2.25)	1.42 (0.89-2.26)
Illiterate/Never went to school	2.76 (1.99-3.82)	2.75 (1.94-3.89)	2.75 (1.98-3.83)	2.76 (1.94-3.92)
**Race/skin color**				
White/Asian	1.00	1.00	1.00	1.00
Pardo	1.19 (1.12-1.27)	1.18 (1.09-1.26)	1.19 (1.12-1.27)	1.18 (1.10-1.27)
Black	1.69 (1.57-1.83)	1.70 (1.57-1.84)	1.68 (1.55-1.82)	1.70 (1.56-1.84)
Indigenous	1.20 (0.82-1.75)	1.17 (0.75-1.83)	1.28 (0.88-1.86)	1.25 (0.81-1.95)
**Wealth levels**				
Level 1 (Higher wealth)	1.00	1.00	1.00	1.00
Level 2	1.17 (1.04-1.32)	1.17 (1.03-1.32)	1.17 (1.04-1.32)	1.16 (1.03-1.32)
Level 3	1.42 (1.21-1.66)	1.45 (1.23-1.71)	1.41 (1.20-1.66)	1.45 (1.23-1.72)
Level 4	1.58 (1.36-1.82)	1.54 (1.32-1.79)	1.57 (1.36-1.82)	1.54 (1.32-1.79)
Level 5 (Lower wealth)	1.99 (1.70-2.34)	2.01 (1.70-2.38)	2.01 (1.72-2.35)	2.03 (1.73-2.40)
**Receipt time of *Bolsa Família***				
Does not receive	1.00	1.00	1.00	1.00
Less than 2 years	1.18 (1.08-1.29)	1.17 (1.07-1.28)	1.18 (1.08-1.28)	1.17 (1.07-1.28)
Between 2 and 5 years	1.05 (0.96-1.14)	1.04 (0.95-1.14)	1.05 (0.96-1.13)	1.04 (0.96-1.14)
Between 5 and 10 years	0.66 (0.61-0.72)	0.65 (0.60-0.71)	0.67 (0.62-0.72)	0.66 (0.61-0.71)
More than 10 years	0.47 (0.42-0.52)	0.48 (0.43-0.55)	0.48 (0.42-0.53)	0.49 (0.43-0.56)
**BLOCK 3 – HOUSEHOLD CONDITIONS**				
**Household material**				
Brick	1.00	1.00	1.00	1.00
Wood	1.31 (1.19-1.43)	1.33 (1.22-1.46)	1.33 (1.22-1.44)	1.35 (1.24-1.48)

Mud and others	1.10 (0.99-1.21)	1.12 (1.00-1.26)	1.18 (1.06-1.31)	1.20 (1.07-1.35)
**Electrical power**				
Yes	1.00	1.00	1.00	1.00
No	1.24 (1.16-1.33)	1.25 (1.16-1.35)	1.25 (1.17-1.33)	1.26 (1.18-1.35)
**Household density (**residents/room)				
up to 2	1.00	1.00	1.00	1.00
more than 2	1.23 (1.12-1.35)	1.20 (1.09-1.33)	1.21 (1.10-1.33)	1.18 (1.07-1.31)
**ADJUSTMENT VARIABLES**				
**Gender**				
Female	1.00	1.00	1.00	1.00
Male	1.27 (1.20-1.34)	1.30 (1.23-1.38)	1.28 (1.21-1.35)	1.31 (1.23-1.39)
**Age**				
Adolescents (13-17 years old)	1.00	1.00	1.00	1.00
Young (17-23.9 years old)	3.84 (3.20-4.61)	3.75 (3.10-4.55)	3.85 (3.21-4.62)	3.77 (3.12-4.56)
24-34.9 years old	9.30 (7.93-10.90)	9.11 (7.73-1.07)	9.26 (7.92-1.08)	9.09 (7.74-1.07)
35-44.9 years	12.31 (10.30-14.70)	1.19 (9.86-1.43)	1.22 (1.02-1.45)	1.18 (9.83-1.42)
45-54.9 years	9.53 (8.02-11.32)	9.52 (7.95-1.14)	9.51 (8.01-1.13)	9.50 (7.94-1.14)
55-64.9 years	5.35 (4.43-6.47)	5.30 (4.34-6.47)	5.34 (4.43-6.45)	5.29 (4.34-6.44)
Elderly (65 years or older)	2.32 (1.83-2.93)	2.33 (1.82-2.99)	2.34 (1.85-2.95)	2.34 (1.83-3.00)
**Year of admission to the cohort**				
2007	1.00	1.00	1.00	1.00
2008	1.17 (1.08-1.27)	1.16 (1.06-1.26)	1.18 (1.10-1.27)	1.16 (1.08-1.26)
2009	1.10 (1.01-1.20)	1.07 (0.98-1.18)	1.11 (1.01-1.21)	1.07 (0.98-1.18)
2010	0.96 (0.88-1.05)	0.97 (0.88-1.07)	0.98 (0.89-1.08)	0.98 (0.89-1.09)
2011	1.11 (0.97-1.26)	1.07 (0.94-1.23)	1.15 (1.01-1.31)	1.10 (0.96-1.26)
2012	1.04 (0.92-1.17)	1.02 (0.90-1.16)	1.08 (0.96-1.22)	1.05 (0.92-1.20)
2013	0.89 (0.77-1.02)	0.88 (0.76-1.01)	0.94 (0.82-1.09)	0.92 (0.79-1.07)
2014	0.89 (0.74-1.08)	0.84 (0.69-1.03)	0.96 (0.80-1.15)	0.88 (0.72-1.07)
2015	1.01 (0.77-1.32)	1.01 (0.77-1.33)	1.08 (0.83-1.40)	1.06 (0.80-1.39)
**Municipal aggregate variables**				
Municipal HIV/AIDS endemicity and surveillance in the period	1.10 (1.081.12)	1.10 (1.08-1.12)	1.10 (1.08-1.12)	1.10 (1.08-1.11)
Coverage of Family Health Strategy - FHS	-	-	0.86 (0.70-1.06)	0.84 (0.66-1.07)
Specialized clinics rate	-	-	1.25 (0.84-1.86)	1.23 (0.81-1.86)
Physicians per 1K	-	-	1.01 (0.93-1.08)	1.01 (0.94-1.09)
Hospital beds per 1K	-	-	1.00 (0.97-1.02)	1.00 (0.97-1.02)
Gini index	-	-	0.50 (0.08-3.12)	0.45 (0.05-3.74)
Extreme poverty rate	-	-	0.42 (0.19-0.89)	0.33 (0.14-0.77)
Unemployment rate	-	-	7.81 (1.90-3.20)	5.32 (1.07-2.66)

Model 1: Original model with all municipalities;
Model 2: Original model only with municipality with adequate Quality of Vital Information;
Model 3: Model with all municipalities and inclusion of municipal aggregate variables for adjustment;
Model 4: Model only with municipality with adequate Quality of Vital Information and inclusion of aggregate municipal variables for adjustment.
Abbreviations: RR: Rate Ratios; CI: confidence interval.

Table S6. Sensitivity Analyses of model AIDS case-fatality rate over the study period: Quality of Vital Information and aggregate municipal variables for adjustment

	Poisson regressions			
	**Model 1** (N =11,815)	**Model 2** (N=11,244)	**Model 3** (N=11,794)	**Model 4** (N=11,234)
	**RR (95% CI)**	**RR (95% CI)**	**RR (95% CI)**	**RR (95% CI)**
**BLOCK 1–GEOGRAPHIC FACTORS**				
**Region of residence**				
North	1.00	1.00	1.00	
Northeast	0.96 (0.76-1.21)	1.00 (0.79-1.27)	1.01 (0.79-1.30)	1.04 (0.80-1.35)
Southeast	0.81 (0.64-1.03)	0.82 (0.64-1.05)	0.89 (0.68-1.17)	0.90 (0.68-1.20)
South	0.92 (0.71-1.18)	0.93 (0.71-1.20)	0.98 (0.72-1.34)	0.98 (0.71-1.36)
Mid-West	1.09 (0.82-1.45)	0.99 (0.75-1.32)	1.11 (0.82-1.50)	1.02 (0.74-1.39)
**BLOCK 2–SOCIOECONOMIC STATUS**				
**Education**				
Higher education	1.00	1.00	1.00	1.00
High school	1.35 (0.75-2.42)	1.28 (0.71-2.30)	1.36 (0.76-2.45)	1.29 (0.72-2.33)
Elementary school	1.73 (0.97-3.06)	1.63 (0.92-2.89)	1.75 (0.98-3.11)	1.64 (0.92-2.91)
Literate/pre-school	1.23 (0.52-2.90)	1.15 (0.47-2.79)	1.21 (0.52-2.85)	1.13 (0.47-2.75)
Illiterate/Never went to school	1.86 (1.02-3.41)	1.82 (0.99-3.33)	1.85 (1.01-3.41)	1.80 (0.98-3.30)
**Race/skincolor**				
White/Asian	1.00	1.00	1.00	1.00
Pardo	1.09 (0.96-1.24)	1.08 (0.95-1.24)	1.09 (0.96-1.24)	1.09 (0.96-1.24)
Black	1.14 (1.01-1.30)	1.17 (1.04-1.33)	1.14 (1.00-1.29)	1.17 (1.03-1.32)
Indigenous	0.58 (0.22-1.55)	0.69 (0.26-1.86)	0.58 (0.22-1.57)	0.69 (0.26-1.87)
**Wealth levels**				
Level1 (Higher wealth)	1.00	1.00	1.00	1.00
Level2	1.18 (0.90-1.53)	1.11 (0.85-1.45)	1.18 (0.91-1.54)	1.12 (0.85-1.47)
Level3	1.07 (0.79-1.45)	1.06 (0.78-1.44)	1.08 (0.79-1.47)	1.07 (0.78-1.46)
Level4	1.01 (0.78-1.32)	0.95 (0.73-1.24)	1.01 (0.77-1.31)	0.96 (0.73-1.25)
Level5 (Lower wealth)	1.09 (0.81-1.45)	1.03 (0.77-1.38)	1.07 (0.80-1.43)	1.02 (0.76-1.38)
**Receipt time of *Bolsa Família***				
Does not receive	1.00	1.00	1.00	1.00
Less than 2years	1.23 (1.02-1.50)	1.25 (1.02-1.53)	1.23 (1.01-1.50)	1.25 (1.02-1.54)
Between 2 and 5 years	1.02 (0.87-1.21)	1.04 (0.87-1.23)	1.02 (0.86-1.21)	1.03 (0.87-1.23)
Between 5 and 10 years	0.77 (0.65-0.91)	0.76 (0.63-0.90)	0.77 (0.65-0.91)	0.75 (0.63-0.89)
More than 10 years	0.57 (0.46-0.72)	0.58 (0.46-0.73)	0.56 (0.44-0.70)	0.57 (0.45-0.72)
**BLOCK 3–HOUSEHOLD CONDITIONS**				
**Household density (**residents/room)				
Up to 2	1.00	1.00	1.00	1.00
More than 2	1.35 (1.12-1.63)	1.32 (1.09-1.61)	1.38 (1.14-1.66)	1.36 (1.12-1.65)
**Sewage system**				
Sewage network	1.00	1.00	1.00	1.00

Septictank	1.05 (0.89-1.24)	1.04 (0.88-1.23)	1.05 (0.89-1.24)	1.04 (0.87-1.23)
Others	1.17 (1.03-1.33)	1.19 (1.05-1.36)	1.16 (1.02-1.32)	1.18 (1.04-1.34)
**BLOCK 4–HEALTH AND BEHAVIORAL ASPECTS**				
**Treatment**				
Yes	1.00	1.00	1.00	1.00
No	2.58 (2.22-3.00)	2.52 (2.16-2.93)	2.54 (2.16-2.99)	2.49 (2.12-2.93)
**Exposure Category**				
Homosexual	1.00	1.00	1.00	1.00
Bisexual	1.06 (0.79-1.43)	1.03 (0.77-1.38)	1.08 (0.80-1.44)	1.04 (0.78-1.39)
Heterosexual	1.56 (1.29-1.90)	1.49 (1.22-1.81)	1.55 (1.28-1.89)	1.48 (1.21-1.80)
People who inject drugs	3.05 (2.36-3.94)	3.01 (2.32-3.90)	3.09 (2.39-3.99)	3.04 (2.35-3.94)
**ADJUSTMENT VARIABLES**				
**Gender**				
Female	1.00	1.00	1.00	1.00
Male	1.36 (1.21-1.54)	1.33 (1.18-1.50)	1.35 (1.20-1.53)	1.31 (1.17-1.48)
**Age**				
Adolescents (13-17yearsold)	1.00	1.00	1.00	1.00
Young (17-23.9 years old)	0.88 (0.66-1.18)	0.86 (0.64-1.15)	0.87 (0.65-1.17)	0.85 (0.63-1.13)
24-34.9 years old	1.11 (0.82-1.50)	1.08 (0.80-1.47)	1.10 (0.81-1.48)	1.07 (0.79-1.44)
35-44.9 years	1.24 (0.92-1.67)	1.21 (0.89-1.62)	1.23 (0.92-1.66)	1.20 (0.89-1.61)
45-54.9years	1.39 (1.01-1.93)	1.33 (0.96-1.84)	1.39 (1.01-1.93)	1.32 (0.95-1.83)
55-64.9 years	2.46 (1.75-3.46)	2.34 (1.66-3.30)	2.43 (1.73-3.42)	2.32 (1.64-3.26)
Elderly (65 years or older)	3.25 (2.05-5.15)	2.87 (1.79-4.59)	3.27 (2.06-5.19)	2.87 (1.79-4.59)
**Year of admission to the cohort**				
2007	1.00	1.00	1.00	1.00
2008	1.11 (0.95-1.29)	1.10 (0.94-1.29)	1.12 (0.97-1.31)	1.12 (0.96-1.31)
2009	1.24 (1.07-1.44)	1.24 (1.06-1.44)	1.25 (1.07-1.46)	1.24 (1.06-1.46)
2010	1.04 (0.85-1.27)	1.04 (0.85-1.27)	1.05 (0.86-1.29)	1.04 (0.84-1.29)
2011	1.13 (0.86-1.48)	1.09 (0.82-1.44)	1.12 (0.84-1.49)	1.08 (0.80-1.45)
2012	1.28 (1.01-1.64)	1.25 (0.97-1.60)	1.27 (0.97-1.65)	1.23 (0.93-1.63)
2013	1.35 (0.93-1.97)	1.41 (0.97-2.05)	1.33 (0.89-1.97)	1.38 (0.93-2.06)
2014	2.74 (1.96-3.81)	2.68 (1.91-3.76)	2.65 (1.85-3.79)	2.60 (1.79-3.77)
2015	4.59 (2.29-9.20)	4.53 (2.26-9.08)	4.58 (2.24-9.39)	4.55 (2.22-9.35)
**Municipal aggregate variables**				
Municipal HIV/AIDS endemicity and surveillance in the period	1.02 (1.01-1.02)	1.02 (1.01-1.02)	1.02 (1.01-1.02)	1.02 (1.01-1.02)
Coverage of Family Health Strategy - FHS	-	-	0.82 (0.58-1.16)	0.84 (0.59-1.21)
Specialized clinics rate	-	-	0.77 (0.45-1.34)	0.85 (0.48-1.50)
Physicians per 1K	-	-	0.94 (0.85-1.03)	0.96 (0.87-1.05)
Hospital beds per 1K	-	-	1.05 (1.01-1.09)	1.05 (1.01-1.08)
Gini index	-	-	1.65 (0.46-5.99)	1.23 (0.33-4.53)
Extreme poverty rate	-	-	1.24 (0.36-4.26)	1.94 (0.53-7.07)
Unemployment rate	-	-	0.19 (0.02-2.25)	0.23 (0.02-3.16)

Model 1: Original model with all municipalities;

Model 2: Original model only with municipality with adequate Quality of Vital Information;
Model 3: Model with all municipalities and inclusion of municipal aggregate variables for adjustment;
Model 4: Model only with municipality with adequate Quality of Vital Information and inclusion of aggregate municipal variables for adjustment.
Abbreviations: RR: Rate Ratios; CI: confidence interval

Considering possible changes over time and possible interferences in the effects of SDH on AIDS outcomes, we conducted stratified analyses by time period as sensitivity analysis. For this purpose, two approaches were considered:

(a) We compared the population strata by the year of entry into the cohort, considering the middle of the period. Thus those who entered the cohort between 2007 and 2010 were compared to those who entered between 2011 and 2015. No major changes in the estimates were observed which reveal that over time the effects of SDH on AIDS outcomes hold. It is observed that for some of the social determinants, such as education, race/skin color, wealth levels, and time of receipt of *Bolsa Família*, there is an increase in the effects on AIDS cases and deaths over time (Tables S7 and S8). This also allows us to infer that after 2015 the AIDS outcomes are still influenced by the SDH, and there may be an increase in the association forces if no intervention is made.

b) We also made comparisons considering the year 2013 as a temporal change point, since between 2013 and 2014 in Brazil there were some changes in notification policies - inclusion of compulsory notification for HIV in the entire population (Ordinance No. 1,271, June 6, 2014)[11] - and initiation of ART - all individuals diagnosed with HIV start treatment regardless of laboratory tests.[12] Thus, those who entered the cohort between 2007 and 2013 were compared with those who entered between 2014 and 2015. There were also no major changes in the estimates, with increased effects for some of the determinants assessed, such as level of wealth and time of receipt of Bolsa Familia (Tables S7 and S8). These findings confirm that there is no change in our findings, even with the policy changes that have occurred. This was expected since the policies are aimed at people diagnosed with HIV and not AIDS, which is our diagnosis of interest.

Table S7. Sensitivity Analyses of model AIDS incidence rate: Effects over time

	**Poisson regressions models by admission periods in the cohort**			
	**2007 to 2010** (N =10,194,822)	**2011 to 2015** (N=12,274,060)	**2007 to 2013** (N=17,669,849)	**2014 to 2015** (N=4,799,033)
	**RR (95% CI)**	**RR (95% CI)**	**RR (95% CI)**	**RR (95% CI)**
**Failures (AIDS cases)**	20,317	7,583	26,793	1,107
**Person-years (py) at risk**	100,550,213	37,436,768.6	132,286,559	5,700,422.9
AIDS incidence rate **/100,000 py**	20.21 (19.93-20.49)	20.25 (19.80-20.72)	20,25 (20,01-20,50)	19.41 (18.31-20.60)
**BLOCK 1 – GEOGRAPHIC FACTORS**				
**Region of residence**				
North	1.00	1.00	1.00	1.00
Northeast	1.27 (1.13-1.44)	1.10 (0.95-1.27)	1.23 (1.11-1.36)	1.23 (0.96-1.59)
Southeast	1.38 (1.23-1.54)	1.03 (0.89-1.20)	1.27 (1.16-1.40)	1.12 (0.87-1.43)
South	1.60 (1.42-1.80)	1.25 (1.06-1.48)	1.51 (1.37-1.66)	1.13 (0.82-1.56)
Mid-West	1.35 (1.18-1.55)	1.13 (0.93-1.37)	1.29 (1.14-1.45)	1.13 (0.77-1.65)
**Area of residence**				
Rural	1.00	1.00	1.00	1.00
Urbana	2.21 (2.05-2.38)	2.00 (1.76-2.26)	2.18 (2.02-2.34)	1.84 (1.43-2.38)
**BLOCK 2 – SOCIOECONOMIC STATUS**				
**Education**				
Higher education	1.00	1.00	1.00	1.00
High school	1.06 (0.89-1.26)	0.86 (0.72-1.03)	0.95 (0.83-1.09)	0.96 (0.67-1.36)
Elementary school	1.51 (1.27-1.79)	1.28 (1.05-1.56)	1.41 (1.22-1.63)	1.42 (0.99-2.05)
Literate/pre-school	1.29 (1.00-1.67)	0.85 (0.59-1.22)	1.09 (0.87-1.36)	0.93 (0.41-2.14)
Illiterate/Never went to school	1.54 (1.29-1.84)	1.36 (1.12-1.66)	1.43 (1.23-1.66)	1.64 (1.05-2.56)
**Race/skin color**				
White/Asian	1.00	1.00	1.00	1.00
Pardo	1.14 (1.09-1.19)	1.23 (1.13-1.34)	1.17 (1.12-1.22)	1.20 (1.00-1.45)
Black	1.48 (1.40-1.57)	1.64 (1.49-1.80)	1.53 (1.46-1.62)	1.49 (1.18-1.89)

Indigenous	1.08 (0.81-1.44)	1.20 (0.79-1.82)	1.10 (0.85-1.43)	1.37 (0.48-3.93)
**Wealth levels**				
Level 1 (Higher wealth)	1.00	1.00	1.00	1.00
Level 2	1.01 (0.83-1.22)	1.06 (0.98-1.13)	1.06 (0.99-1.14)	1.08 (0.90-1.30)
Level 3	1.00 (0.84-1.20)	1.29 (1.18-1.41)	1.22 (1.13-1.33)	1.45 (1.19-1.76)
Level 4	0.92 (0.77-1.09)	1.79 (1.61-1.99)	1.25 (1.15-1.35)	2.05 (1.56-2.68)
Level 5 (Lower wealth)	1.13 (0.96-1.35)	2.45 (2.04-2.95)	1.47 (1.36-1.60)	3.52 (2.52-4.93)
**Receipt time of *Bolsa Família***				
Does not receive	1.00	1.00	1.00	1.00
Less than 2 years	1.09 (1.01-1.19)	1.09 (1.01-1.17)	1.09 (1.02-1.15)	1.03 (0.88-1.19)
Between 2 and 5 years	1.09 (1.02-1.16)	0.98 (0.92-1.06)	1.05 (1.00-1.10)	0.95 (0.71-1.27)
Between 5 and 10 years	0.86 (0.80-0.93)	0.74 (0.67-0.83)	0.86 (0.81-0.91)	0.52 (0.17-1.60)
More than 10 years	0.71 (0.65-0.77)	0.51 (0.40-0.65)	0.70 (0.66-0.75)	0.50 (0.16-1.56)
**BLOCK 3 – HOUSEHOLD CONDITIONS**				
**Household material**				
Brick	1.00	1.00	1.00	1.00
Wood	1.20 (1.12-1.29)	1.22 (1.11-1.34)	1.20 (1.13-1.27)	1.43 (1.14-1.79)
Mud and others	1.11 (1.04-1.19)	0.81 (0.66-0.99)	1.07 (1.00-1.14)	1.07 (0.69-1.67)
**Electrical power**				
Yes	1.00	1.00	1.00	1.00
No	1.19 (1.13-1.25)	1.03 (0.94-1.13)	1.15 (1.09-1.21)	1.01 (0.81-1.26)
**Household density (**residents/room)				
up to 2	1.00	1.00	1.00	1.00
more than 2	1.15 (1.08-1.22)	1.11 (0.94-1.31)	1.16 (1.09-1.23)	0.75 (0.45-1.24)
**ADJUSTMENT VARIABLES**				
**Gender**				
Female	1.00	1.00	1.00	1.00
Male	1.03 (0.97-1.09)	1.79 (1.61-1.98)	1.16 (1.09-1.23)	2.48 (2.03-3.03)
**Age**				
Adolescents (13-17 years old)	1.00	1.00	1.00	1.00

Young (17-23.9 years old)	2.67 (2.46-2.90)	5.91 (4.60-7.59)	2.88 (2.66-3.12)	10.42 (3.92-27.75)
24-34.9 years old	4.46 (4.12-4.83)	10.55 (8.10-13.76)	4.77 (4.41-5.16)	20.02 (8.19-48.93)
35-44.9 years	4.60 (4.24-4.99)	13.30 (10.31-17.16)	5.14 (4.76-5.55)	25.55 (10.37-62.96)
45-54.9 years	3.13 (2.83-3.47)	11.60 (9.00-14.96)	3.79 (3.45-4.17)	24.99 (10.67-58.52)
55-64.9 years	1.68 (1.48-1.91)	6.01 (4.54-7.95)	2.07 (1.84-2.33)	10.73 (4.34-26.53)
Elderly (65 years or older)	0.59 (0.48-0.71)	2.58 (1.94-3.42)	0.80 (0.70-0.92)	5.61 (2.14-14.70)
**Year of admission to the cohort**				
2007	1.00		1.00	
2008	1.12 (1.07-1.18)		1.13 (1.07-1.18)	
2009	1.10 (1.04-1.17		1.10 (1.04-1.17)	
2010	1.04 (0.98-1.11)		1.06 (1.00-1.13)	
2011		1.00	1.08 (0.99-1.16)	
2012		0.94 (0.87-1.00)	1.05 (0.97-1.14)	
2013		0.84 (0.77-0.92)	0.96 (0.88-1.05)	
2014		0.83 (0.76-0.90)		1.00
2015		0.75 (0.63-0.89)		0.90 (0.77-1.06)
**Municipal HIV/AIDS endemicity and surveillance in the period (2007-2015)**				
Average AIDS incidence rate	1.03 (1.03-1.04)	1.03 (1.03-1.03)	1.03 (1.03-1.04)	1.03 (1.02-1.03)

Abbreviations: RR: Rate Ratios; CI: confidence interval

Table S8. Sensitivity Analyses of model AIDS mortality rate: Effects over time

	Poisson regressions models by admission periods in the cohort			
	**2007 to 2010** (N =10,194,830)	**2011 to 2015** (N=12,274,073)	**2007 to 2013** (N=17,669,863)	**2014 to 2015** (N=4,799,040)
	**RR (95% CI)**	**RR (95% CI)**	**RR (95% CI)**	**RR (95% CI)**
**Failures (AIDS death)**	7,036	2,492	9,169	359
**Person-years (py) at risk**	100,617,276	37,451,244.2	132,366,857	5,701,663
**AIDS Mortality rate /100,000 py**	6.99 (6.83-7.16)	6.65 (6.40-6.92)	6.93 (6.79-7.07)	6.30 (5.68-6.98)
**BLOCK 1 – GEOGRAPHIC FACTORS**				
**Region of residence**				
North	1.00	1.00	1.00	1.00
Northeast	1.22 (1.06-1.40)	0.98 (0.80-1.20)	1.17 (1.04-1.33)	0.96 (0.64-1.44)
Southeast	1.55 (1.29-1.87)	1.22 (0.91-1.62)	1.47 (1.21-1.78)	1.38 (0.89-2.15)
South	1.47 (1.22-1.78)	1.13 (0.87-1.46)	1.38 (1.16-1.65)	1.12 (0.65-1.94)
Mid-West	1.29 (1.08-1.55)	1.01 (0.79-1.30)	1.23 (1.04-1.45)	0.88 (0.50-1.54)
**Area of residence**				
Rural	1.00	1.00	1.00	1.00
Urbana	2.40 (2.13-2.71)	2.16 (1.77-2.64)	2.39 (2.14-2.67)	1.51 (1.01-2.29)
**BLOCK 2 – SOCIOECONOMIC STATUS**				
**Education**				
Higher education	1.00	1.00	1.00	1.00
High school	1.14 (0.79-1.66)	1.29 (0.80-2.07)	1.20 (0.85-1.68)	1.38 (0.59-3.25)
Elementary school	2.25 (1.59-3.20)	2.41 (1.52-3.84)	2.44 (1.77-3.36)	2.28 (1.02-5.09)
Literate/pre-school	1.51 (0.91-2.51)	1.19 (0.57-2.49)	1.37 (0.88-2.14)	2.49 (0.71-8.74)
Illiterate/Never went to school	2.44 (1.68-3.55)	2.96 (1.84-4.74)	2.71 (1.94-3.80)	2.37 (0.96-5.87)
**Race/skin color**				
White/Asian	1.00	1.00	1.00	1.00
Pardo	1.16 (1.08-1.24)	1.29 (1.14-1.46)	1.18 (1.11-1.27)	1.47 (1.12-1.92)
Black	1.67 (1.54-1.81)	1.75 (1.49-2.05)	1.70 (1.57-1.83)	1.65 (1.13-2.41)
Indigenous	0.91 (0.56-1.47)	2.32 (1.23-4.38)	1.07 (0.71-1.63)	4.21 (1.24-14.28)
**Wealth levels**				
Level 1 (Higher wealth)	1.00	1.00	1.00	1.00
Level 2	1.08 (0.77-1.51)	1.18 (1.03-1.34)	1.16 (1.03-1.31)	1.21 (0.85-1.72)
Level 3	1.06 (0.77-1.47)	1.52 (1.29-1.78)	1.37 (1.17-1.60)	1.65 (1.11-2.44)
Level 4	0.96 (0.71-1.31)	2.53 (2.12-3.02)	1.44 (1.25-1.67)	3.24 (2.16-4.84)
Level 5 (Lower wealth)	1.29 (0.95-1.75)	3.53 (2.57-4.85)	1.84 (1.56-2.17)	5.70 (3.39-9.58)
**Receipt time of *Bolsa Família***				
Does not receive	1.00	1.00	1.00	1.00
Less than 2 years	1.27 (1.13-1.43)	1.09 (0.96-1.24)	1.21 (1.10-1.32)	0.91 (0.72-1.16)
Between 2 and 5 years	1.16 (1.06-1.27)	0.87 (0.76-1.00)	1.05 (0.97-1.15)	0.80 (0.46-1.39)
Between 5 and 10 years	0.69 (0.63-0.77)	0.47 (0.38-0.57)	0.67 (0.61-0.72)	0.00 (0.00-0.00)
More than 10 years	0.49 (0.43-0.57)	0.27 (0.17-0.44)	0.47 (0.42-0.53)	0.00 (0.00-0.00)

**BLOCK 3 – HOUSEHOLD CONDITIONS**				
**Household material**				
Brick	1.00	1.00	1.00	1.00
Wood	1.31 (1.18-1.47)	1.22 (1.04-1.44)	1.30 (1.19-1.42)	1.39 (0.91-2.13)
Mud and others	1.16 (1.04-1.28)	0.73 (0.52-1.01)	1.10 (1.00-1.22)	0.95 (0.43-2.11)
**Electrical power**				
Yes	1.00	1.00	1.00	1.00
No	1.31 (1.21-1.41)	1.04 (0.91-1.19)	1.24 (1.15-1.33)	1.29 (0.89-1.86)
**Household density (**residents/room)				
up to 2	1.00	1.00	1.00	1.00
more than 2	1.20 (1.09-1.33)	1.32 (1.02-1.72)	1.24 (1.13-1.37)	0.48 (0.19-1.22)
**ADJUSTMENT VARIABLES**				
**Gender**				
Female	1.00	1.00	1.00	1.00
Male	1.08 (1.01-1.15)	2.01 (1.81-2.23)	1.23 (1.16-1.30)	2.45 (1.86-3.22)
**Age**				
Adolescents (13-17 years old)	1.00	1.00	1.00	1.00
Young (17-23.9 years old)	3.74 (3.07-4.56)	4.65 (2.67-8.10)	3.86 (3.22-4.62)	3.34 (0.74-15.00)
24-34.9 years old	9.29 (7.80-1.11)	11.79 (6.83-20.35)	9.29 (7.93-10.88)	11.63 (3.19-42.37)
35-44.9 years	11.27 (9.33-1.36)	22.04 (12.27-39.61)	12.14 (10.19-14.47)	22.84 (6.03-86.54)
45-54.9 years	7.66 (6.35-9.25)	20.92 (11.52-37.98)	9.16 (7.70-10.89)	25.78 (7.34-90.53)
55-64.9 years	4.38 (3.55-5.39)	10.92 (5.89-20.23)	5.18 (4.27-6.27)	12.73 (3.42-47.38)
Elderly (65 years or older)	1.67 (1.23-2.25)	5.14 (2.78-9.52)	2.22 (1.76-2.80)	6.33 (1.55-25.93)
**Year of admission to the cohort**				
2007	1.00		1.00	
2008	1.17 (1.08-1.26)		1.17 (1.09-1.27)	
2009	1.10 (1.00-1.20)		1.10 (1.01-1.20)	
2010	0.93 (0.85-1.02)		0.96 (0.87-1.05)	
2011		1.00	1.05 (0.92-1.20)	
2012		0.87 (0.77-0.98)	0.99 (0.87-1.12)	
2013		0.73 (0.63-0.84	0.84 (0.73-0.97)	
2014		0.70 (0.58-0.85)		1.00
2015		0.76 (0.58-1.00)		1.09 (0.86-1.38)
**Municipal HIV/AIDS endemicity and surveillance in the period (2007-2015)**				
Average AIDS mortality rate	1.11 (1.08-1.13)	1.09 (1.07-1.12)	1.10 (1.08-1.13)	1.08 (1.05-1.12)

Abbreviations: RR: Rate Ratios; CI: confidence interval

These analyses by periods are limited because AIDS is a disease with a long latency period, that is, from infection to progression to advanced disease, and from this to death it takes several years. The analyses with a short follow-up period certainly would not allow us to see the effects on HIV infection, but only on advanced disease (AIDS) and deaths from this cause. This may explain the loss of statistical significance in some associations for the later periods. Thus, we chose to maintain the model that considers the 9 years of follow-up.

4. Descriptive analysis

The population of the cohort studied was mostly female (52.69%), young adults (24-34.9 years old) (29.41%), residing in the Northeast (32.88%) and Southeast (36.77%) regions and in the urban area of residence (80.38%). As for the socioeconomic status (Block 2), most individuals of the cohort had Elementary school (59%), were Pardo (57.52%), categorized as level 5 of wealth (lower wealth) (28.07%) and receipted of *Bolsa Família* (64.75%). It should be noted that the variable race/skin color originally had five response categories (White, Yellow/Asian, Pardo, Black, and Indigenous), however the yellow/Asian category represents a very small portion of the Brazilian population, in our cohort only 0.47% of individuals declare themselves yellow/Asian and also represents a small number of cases and deaths from AIDS (Table S8). Due to the small number of representatives, for having, in their majority, socioeconomic characteristics similar to those of individuals who self-declare white in the country, and for presenting low AIDS incidence and mortality rates (lower than those of whites, the reference group) (Table S10), we opted to join these two categories of race/skin color (White/Asian) and emphasize those who represent the most vulnerable groups: Pardo, Black, and Indigenous.

It was identified that most individuals in the cohort lived in favorable household conditions (Block 3), with the majority living in households with Brick material (81.34%), with Electrical power (88.88%), with low Household density (up to 2 residents for room) (95.5%), with Public network Water supply (76.10%), public collection of Waste disposal (80.78%) and with sewage system sanitary (52.18%) (Table S8).

For the AIDS cases similar proportions are observed in the regions, with more cases in the Northeast (28.63%) and Southeast (33.98%) regions. Higher proportions of AIDS cases than in the general population were observed in males (54.03%) and young adults (24-34.9 years old) (38.76 %), in the urban area of residence (90.96%), for those with Elementary school (70.32%), the Black race (12.98%) and Lower wealth (42.32%). Similarly, for AIDS death is observed in males (56.61%) and young adults (24-34.9 years old) (35.26%), in the Southeast (41.32%), in the urban area of residence (91.56%), those with Elementary school (73.89%), the Black race (14.84) and Lower wealth (43.48%). In both cases, few differences in concentration were observed in Block 3 (Table S8).

Among People Living with AIDS (PLWA) who died, 80.28% were on treatment, 79.39% were heterosexual, 79.27% way of transmission for sexual, with person of the opposite sex (Table S8).

Table S8. Description of Brazilians aged 13 years and older followed (N = 28,318,532) from the 100 million cohort, Brazil, 2007-2015.

	**Cohort 2007-2015**		**AIDS cases 2007-2015**		**AIDS death 2007-2015**	
	**N = 28,318,532**		**N= 27,919 (0.10%)**		**N= 9,530 (0.03%)**	
	**n**	**%**	**n**	**%**	**n**	**%**
**BLOCK 1 – GEOGRAPHIC FACTORS**						
**Region of residence**	N=28,317,056		N = 27,915		N = 9,530	
North	2,977,321	10.51	2,534	9.08	751	7.88
Northeast	9,309,822	32.88	7,993	28.63	2,372	24.89
Southeast	10,413,346	36.77	9,486	33.98	3,938	41.32
South	3,294,344	11.63	5,967	21.38	1,875	19.67
Mid-West	2,322,223	8.20	1,935	6.93	594	6.23
**Area of residence**	N=28,226,698		N = 27,619		N = 9,457	
Rural	5,537,797	19.62	2,498	9.04	798	8.44
Urbana	22,688,901	80.38	25,121	90.96	8,659	91.56
**BLOCK 2 – SOCIOECONOMIC STATUS**						
**Education**	N= 25,791,042		N = 24,460		N = 8,517	
Illiterate/Never went to school	2,643,471	10.25	2,329	9.52	1,011	11.87
Literate/pre-school	256,290	0.99	161	0.66	48	0.56

Elementary school		15,216,511	59.00		17,200	70.32		6,293	73.89
High school		7,117,514	27.60		4,417	18.06		1,096	12.87
Higher education		557,256	2.16		353	1.44		69	0.81
**Race/skin color**	N= 26,703,914			N=26,428			N= 9,075		
White/		8,987,033	33.66		8,589	32.50		2,947	32.47
Yellow/Asian		126,189	0,47		82	0.31		27	0.30
White/Asian		9,113,222	34.13		8,671	32.81		2,974	32.77
Pardo		15,360,569	57.52		14,223	53.82		4,721	52.02
Black		2,075,977	7.77		3,431	12.98		1,347	14.84
Indigenous		154,146	0.58		103	0.39		33	0.36
**Wealth levels**	N= 28,311,231			N = 27,912			N = 9,528		
Level 1 (Higher wealth)		3,404,700	12.03		1,735	6.22		502	5.27
Level 2		6,558,655	23.17		3,707	13.28		1,227	12.88
Level 3		4,105,170	14.50		2860	10.25		947	9.94
Level 4		6,295,870	22.24		7,797	27.93		2,709	28.43
Level 5 (Lower wealth)		7,946,836	28.07		11,813	42.32		4,143	43.48
**Receipt time of *Bolsa Família***	N= 28,318,532			N = 27,919			N = 9,530		
Does not receive		10,123,253	35.75		6,183	22.15		2,228	23.38
Less than 2 years		4,274,260	15.09		4,174	14.95		1,679	17.62
Between 2 and 5 years		5,014,892	17.71		6,345	22.73		2,373	24.90
Between 5 and 10 years		6,234,167	22.01		8,326	29.82		2,462	25.83
More than 10 years		2,671,960	9.44		2,891	10.35		788	8.27
**BLOCK 3 – HOUSEHOLD CONDITIONS**									
**Household material**	N = 27,554,318			N = 27,120			N = 9,280		
Brick		22,414,010	81.34		21,236	78.30		7,319	78.87
Wood		2,850,419	10.34		3,849	14.19		1,309	14.11
Mud and others		2,289,889	8.31		2,035	7.50		652	7.03
**Electrical power**	N = 27,554,758			N = 27,117			N = 9,278		
Yes		24,490,897	88.88		22,911	84.49		7,750	83.53
No		3,063,861	11.12		4,206	15.51		1,528	16.47
**Household density** (residents/room)	N = 25,864,665			N = 25,334			N = 8,686		
up to 2		24,712,132	95.50		23,526	92.86		8,047	92.64
more than 2		1,152,533	4.50		1,808	7.14		639	7.36
**Water supply**	N = 27,554,776			N = 27,116			N = 9,277		
Public network		20,968,506	76.10		21,935	80.89		7,478	80.61
Others		6,586,270	23.90		5,181	19.11		1,799	19.39
**Waste disposal**	N= 27,554,439			N = 27,117			N = 9,278		
Public collection		22,257,375	80.78		23,886	88.08		8,193	88.31
Not collected		5,297,064	19.22		3,231	11.92		1,085	11.69
**Sewage system**	N= 26,969,569			N = 26,847			N = 9,180		
Sewage network		14,073,902	52.18		15,485	57.68		5,507	59.99
Septic tank		3,942,157	14.62		3,724	13.87		1,169	12.73
Others		8,953,510	33.20		7,638	28.45		2,504	27.28

**BLOCK 4 – HEALTH AND BEHAVIORAL ASPECTS (**only for cases notified in SINAN)						
**Treatment**					N= 23.,385	
Yes	-	-	-	-	18,773	80.28
No	-	-	-	-	4,612	19.72
**Exposure Category**					N=19,205	
Homosexual	-	-	-	-	2,391	12.45
Bisexual	-	-	-	-	843	4.39
Heterosexual			-	-	15,247	79.39
People who inject drugs	-	-	-	-	724	3.77
**Means of transmission**					N=19,232	
Blood transfusion	-	-	-	-	19	0.10
Accident with biological material	-	-	-	-	3	0.02
Sexual, with person of the opposite sex	-	-	-	-	15,246	79.27
Sexual, with person of the same sex or both	-	-	-	-	3,238	16.84
Injecting drugs	-	-	-	-	726	3.77
**ADJUSTMENT VARIABLES**						
**Gender**	N=28,318,531		N = 27,919		N = 9,530	
Female	14,920,049	52.69	12,834	45.97	4,135	43.39
Male	13,398,482	47.31	15,085	54.03	5,395	56.61
**Age**	N = 28,318,529		N = 27,919		N = 9,530	
Mean (sd)		36.18 (16.96)		34.69 (11.80)		37.71 (11.92)
Adolescents (13-17 years old)	2,846,567	10.05	996	3.57	172	1.80
Young (17-23.9 years old)	4,946,257	17.47	4,073	14.59	848	8.90
24-34.9 years old	8,328,072	29.41	10,822	38.76	3,360	35.26
35-44.9 years	4,541,078	16.04	6,82	24.43	2,782	29.19
45-54.9 years	3,032,523	10.71	3,455	12.38	1,511	15.86
55-64.9 years	2,422,982	8.56	1,346	4.82	643	6.75
Elderly (65 years or older)	2,201,050	7.77	407	1.46	214	2.25
**Year of admission to the cohort**	N= 28,318,531		N = 27,919		N = 9,530	
2007	5,986,255	21.14	8,975	32.15	3,076	32.28
2008	2,681,090	9.47	4,583	16.42	1,687	17.70
2009	2,550,458	9.01	3,572	12.79	1,230	12.91
2010	2,521,809	8.91	3,196	11.45	1,044	10.95
2011	2,419,291	8.54	2,202	7.89	730	7.66
2012	3,892,533	13.75	2,764	9.90	934	9.80
2013	3,155,799	11.14	1,517	5.43	470	4.93
2014	3,051,647	10.78	899	3.22	282	2.96
2015	2,059,649	7.27	211	0.76	77	0.81
**Municipal HIV/AIDS endemicity and surveillance in the period (2007-2015)**						
**Average AIDS incidence rate**	N = 28,313,579		N = 27,915		N = 9,530	
Mean (sd)	19.14 (13.98)		29.35 (20.67)		28.98 (19.36)	
Median (IQR)	17.30 (8,25-27,27)		26.72 (16.25-36.23)		27.03 (16.45-36.54)	
**Average AIDS mortality rate**	N =28,313,579		N= 27,915		N=9,530	

Mean (sd)	5.65 (4.36)	8.71 (6.64)	8.98 (6.23)
Median (IQR)	5.02 (2,38-8,10)	7.17 (4.67-10.81)	7.95 (4.96-11.29)
**Average AIDS case-fatality rate**	N = 19,643,624	N = 23,683	N= 8,179
Mean (sd)	30.88 (8.54)	30.60 (7.88)	31.98 (8.19)
Median (IQR)	31.04 (25,52-34,43)	30.60 (25.63-33.08)	31.89 (27.24-34.89)

Abbreviations: sd: standard deviation; IQR: Interquartile Range.

As this is a closed cohort, loss to follow-up in the cohort occurred through death, the rates of loss to follow-up are shown in Figure S1.

Figure S1. Annual loss-to-follow-up rates

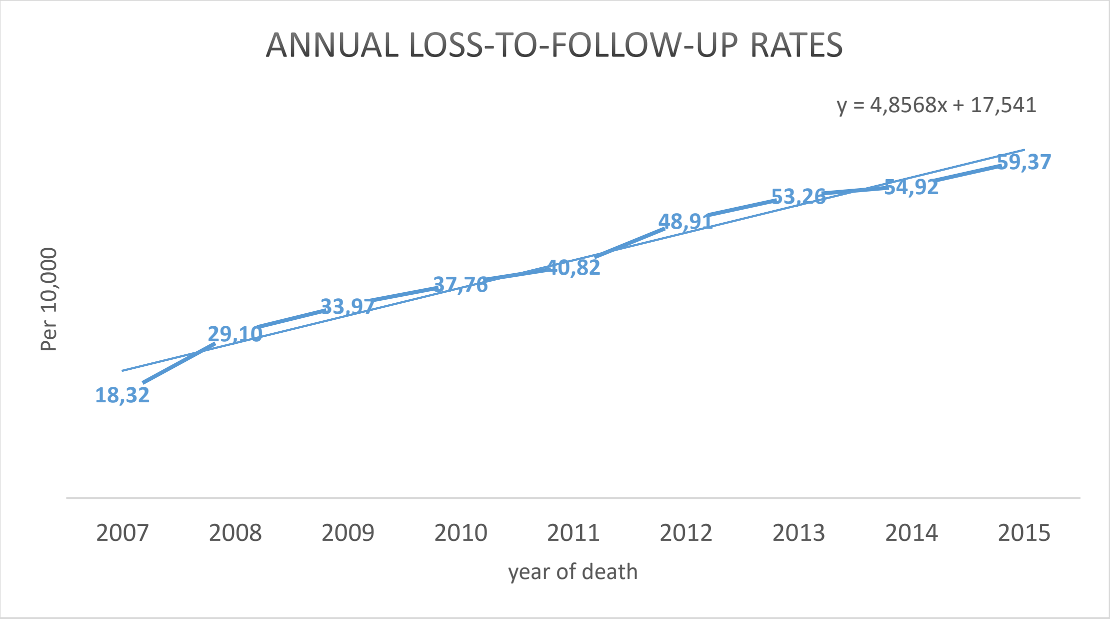

The predominant characteristics of those who died during the follow-up time (n= 717,202; 2.53%), whether from AIDS or other causes, are similar to those observed in the total sample. As for the total sample, those who died mostly residing in the Northeast (30.87%) and Southeast (38.84%) regions and in the urban area of residence (83.68%). As for the socioeconomic status (Block 2), most individuals of the cohort had Elementary school (60.69%), were Pardo (52.41%), categorized as level 5 of wealth (lower wealth) (33.94%) and recipients of Bolsa Família (50.84%). It was identified that most individuals in the cohort lived in favorable household conditions (Block 3), with the majority living in households with Brick material (80.71%), with Electrical power (90.24%), with low Household density (up to 2 residents for room) (96.29%), with Public network

Water supply (78.96%), public collection of Waste disposal (83.14%) and with sewage system sanitary (54.74%). Variations, expected, were observed for gender and age, with a predominance of males (62.34%) and elderly (65 years and older) (39.33%) among those who died during the follow-up period (Table S9).

Table S9. Description of Brazilians aged 13 years and older who died (n = 717,202) during the cohort, Brazil, 2007-2015.

	Loss-to-follow-up cohort 2007-2015		
	N = 717,202		
		**n**	**%**
**BLOCK 1 – GEOGRAPHIC FACTORS**			
**Region of residence**	N=717,183		
North		53,984	7.53
Northeast		221,379	30.87
Southeast		278,522	38.84
South		102,314	14.27
Mid-West		60,984	8.50
**Area of residence**	N=714,837		
Rural		116,661	16.32
Urbana		598,176	83.68
**BLOCK 2 – SOCIOECONOMIC STATUS**			
**Education**	N=669,107		
Illiterate/Never went to school		200,766	30.01
Literate/pre-school		10,632	1.59
Elementary school		406,059	60.69
High school		47,360	7.08
Higher education		4,290	0.64
**Race/skin color**	N=683,265		
White/Asian		256,02	37.47
Pardo		358,084	52.41
Black		66,516	9.74
Indigenous		2,645	0.39
**Wealth levels**	N=716,876		
Level 1 (Higher wealth)		72,803	10.16
Level 2		135,696	18.93
Level 3		75,406	10.52
Level 4		189,690	26.46
Level 5 (Lower wealth)		243,281	33.94
**Receipt time of *Bolsa Família***	N=717,202		
Does not receive		352,606	49.16
Less than 2 years		87,425	12.19
Between 2 and 5 years		107,573	15.00
Between 5 and 10 years		120,671	16.83
More than 10 years		48,927	6.82

**BLOCK 3 – HOUSEHOLD CONDITIONS**			
**Household material**	N=700,740		
Brick		565,542	80.71
Wood		75,217	10.73
Mud and others		59,981	8.56
**Electrical power**	N=700,759		
Yes		632,338	90.24
No		68,421	9.76
**Household density** (residents/room)	N=650,131		
up to 2		626,014	96.29
more than 2		24,117	3.71
**Water supply**	N=700,751		
Public network		553,296	78.96
Others		147,455	21.04
**Waste disposal**	N=700,736		
Public collection		582,624	83.14
Not collected		118,112	16.86
**Sewage system**	N=691,933		
Sewage network		378,787	54.74
Septic tank		99,542	14.39
Others		213,604	30.87
**ADJUSTMENT VARIABLES**			
**Gender**	N=717,202		
Female		270,069	37.66
Male		447,133	62.34
**Age**	N=717,192		
Adolescents (13-17 years old)		20,687	2.88
Young (17-23.9 years old)		38,489	5.37
24-34.9 years old		75,209	10.49
35-44.9 years		72,225	10.07
45-54.9 years		96,186	13.41
55-64.9 years		132,312	18.45
Elderly (65 years or older)		282,084	39.33
**Year of admission to the cohort**	N=717,202		
2007		193,515	26.98
2008		107,789	15.03
2009		79,708	11.11
2010		67,057	9.35
2011		78,240	10.91
2012		107,624	15.01
2013		45,531	6.35
2014		29,535	4.12
2015		8,203	1.14

**Municipal HIV/AIDS endemicity and surveillance in the period (2007-2015)**		
**Average AIDS incidence rate**	N = 717,144	
Mean (sd)		19.12 (14.08)
Median (IQR)		16.91 (8.42-27.27)
**Average AIDS mortality rate**	N = 717,144	
Mean (sd)		5.73 (4.46)
Median (IQR)		5.09 (2.46-8.10)
**Average AIDS case-fatality rate**	N = 492,375	
Mean (sd)		31.45 (8.98)
Median (IQR)		31.06 (25.69-34.85)

Abbreviations: sd: standard deviation; IQR: Interquartile Range.

We also evaluated the characteristics of individuals who were not part of the final model analyses because they were missing from the set of variables included in the Poisson regressions (incomplete cases). Table S10 shows that the characteristics between the study population with complete and incomplete cases for analyses of the final models for each AIDS outcome are similar, suggesting that the missing data are random. Thus, it was possible to use listwise deletion to deal with the missing data for each model estimated in this study.

Table S10. Analyses of missing data: comparing characteristics between the study population with complete and incomplete cases for analyses of the final models for each AIDS outcome.

	**AIDS incidence rate outcome**				**AIDS mortality rate outcome**				**AIDS case fatality rate outcome**			
	**Complete cases (N= 22,468,882)**		**Incomplete cases (N= 5,849,650)**		**Complete cases (N= 22,468,903)**		**Incomplete cases (N= 5,849,629)**		**Complete cases (N=11,815)**		**Incomplete cases (N= 11,570)**	
	**n**	**%**	**n**	**%**	**n**	**%**	**n**	**%**	**n**	**%**	**n**	**%**
**BLOCK 1 – GEOGRAPHIC FACTORS**												
**Region of residence**	N= 22,468,882		N= 5,848,174		N= 22,468,903		N= 5,848,153		N= 11,815		N= 11,566	
North	2,331,725	10.38	645,600	11.04	2,331,726	10.38	645,600	11.04	1,149	9.72	1,039	8.98
Northeast	7,319,456	32.58	1,990,366	34.03	7,319,458	32.58	1,990,364	34.03	3,126	26.46	3,944	34.10
Southeast	8,275,579	36.83	2,137,767	36.55	8,275,588	36.83	2,137,758	36.55	3,753	31.76	3,582	30.97
South	2,666,968	11.87	627,400	10.73	2,666,976	11.87	627,370	10.73	2,897	24.52	2,176	18.81
Mid-West	1,875,154	8.35	447,100	7.64	1,875,155	8.35	447,070	7.64	890	7.53	825	7.13
**Area of residence**	N= 22,468,882		N= 5,757,816		N= 22,468,903		N= 5,757,795		N= 11,815		N= 11,312	
Rural	4,528,542	20.15	1,009,255	17.53	4,528,544	20.15	1,009,253	17.53	720	6.09	1,425	12.60
Urbana	17,940,340	79.85	4,748,561	82.47	17,940,359	79.85	4,748,542	82.47	11,095	93.91	9,887	87.40
**BLOCK 2 – SOCIOECONOMIC STATUS**												
**Education**	N= 22,468,882		N= 3,322,160		N= 22,468,903		N= 3,322,139		N= 11,815		N= 8,555	
Illiterate/Never went to school	2,317,794	10.32	325,700	9.80	2,317,795	10.32	325,680	9.80	945	8.00	912	10.66
Literate/pre-school	234,411	1.04	21,880	0.66	234,411	1.04	21,879	0.66	81	0.69	57	0.67

Elementary school	13,486,515	60.02	1,729,996	52.07	13,486,529	60.02	1,729,982	52.07	8,311	70.34	5,844	68.31
High school	5,956,358	26.51	1,161,156	34.95	5,956,363	26.51	1,161,151	34.95	2,281	19.31	1,620	18.94
Higher education	473,804	2.11	83,45	2.51	473,805	2.11	83,451	2.51	197	1.67	122	1.43
**Race/skin color**	N= 22,468,882		N= 4,235,032		N= 22,468,903		N= 4,235,011		N= 11,815		N= 10,274	
White/Asian	7,755,124	34.51	1,358,098	32.07	7,755,132	34.51	1,358,090	32.07	4	33.86	3,168	30.84
Pardo	12,848,613	57.18	2,511,956	59.31	12,848,624	57.18	2,511,945	59.31	6,220	52.64	5,857	57.01
Black	1,738,665	7.74	337,300	7.96	1,738,667	7.74	337,310	7.96	1,555	13.16	1,201	11.69
Indigenous	126,48	0.56	27,67	0.65	126,48	0.56	27,666	0.65	40	0.34	48	0.47
**Wealth levels**	N= 22,468,882		N= 5,842,349		N= 22,468,903		N= 5,842,328		N= 11,815		N= 11,563	
Level 1 (Higher wealth)	2,870,218	12.77	534,500	9.15	2,870,220	12.77	534,480	9.15	821	6.95	649	5.61
Level 2	5,381,013	23.95	1,177,642	20.16	5,381,018	23.95	1,177,637	20.16	1,604	13.58	1,499	12.96
Level 3	3,247,846	14.45	857,300	14.67	3,247,849	14.45	857,320	14.67	1,155	9.78	1,240	10.72
Level 4	4,986,050	22.19	1,309,820	22.42	4,986,056	22.19	1,309,814	22.42	3,522	29.81	3,025	26.16
Level 5 (Lower wealth)	5,983,755	26.63	1,963,081	33.60	5,983,760	26.63	1,963,076	33.60	4,713	39.89	5,150	44.54
**Receipt time of Bolsa Família**	N= 22,468,882		N= 5,849,650		N= 22,468,903		N= 5,849,629		N= 11,815		N= 11,570	
Does not receive	8,318,006	37.02	1,805,247	30.86	8,318,014	37.02	1,805,239	30.86	2,492	21.09	2,526	21.83
Less than 2 years	3,435,927	15.29	838,300	14.33	3,435,931	15.29	838,330	14.33	1,564	13.24	1,791	15.48
Between 2 and 5 years	3,805,984	16.94	1,208,908	20.67	3,805,988	16.94	1,208,904	20.67	2,634	22.29	2,577	22.27
Between 5 and 10 years	4,766,581	21.21	1,467,586	25.09	4,766,586	21.21	1,467,581	25.09	3,858	32.65	3,410	29.47
More than 10 years	2,142,384	9.53	529,600	9.05	2,142,384	9.53	529,580	9.05	1,267	10.72	1,266	10.94
**BLOCK 3 – HOUSEHOLD CONDITIONS**												
**Household material**	N= 22,468,882		N= 5,085,436		N= 22,468,903		N= 5,085,415		N= 11,815		N= 10,907	
Brick	18,414,583	81.96	3,999,427	78.64	18,414,598	81.96	3,999,412	78.64	9,348	79.12	8,369	76.73
Wood	2,412,535	10.74	437,900	8.61	2,412,540	10.74	437,880	8.61	1,868	15.81	1,394	12.78
Mud and others	1,641,764	7.31	648,100	12.74	1,641,765	7.31	648,120	12.74	599	5.07	1,144	10.49
**Electrical power**	N= 22,468,882		N= 5,085,876		N= 22,468,903		N= 5,085,855		N= 11,815		N= 10,904	
Yes	19,894,871	88.54	4,596,026	90.37	19,894,885	88.54	4,596,012	90.37	9,882	83.64	9,307	85.35
No	2,574,011	11.46	489,900	9.63	2,574,018	11.46	489,840	9.63	1,933	16.36	1,597	14.65
**Household density (**residents/room)	N= 22,468,882		N= 3,395,783		N= 22,468,903		N= 3,395,762		N= 11,815		N= 9,397	
up to 2	21,449,573	95.46	3,262,559	96.08	21,449,593	95.46	3,262,539	96.08	10,887	92.15	8,791	93.55
more than 2	1,019,309	4.54	133,200	3.92	1,019,310	4.54	133,220	3.92	928	7.85	606	6.45
**Water supply**	N= 22,468,780		N= 5,085,996		N= 22,468,801		N= 5,085,975		N= 11,815		N= 10,904	
Public network	16,990,669	75.62	3,977,837	78.21	16,990,687	75.62	3,977,819	78.21	9,740	82.44	8,622	79.07
Others	5,478,111	24.38	1,108,159	21.79	5,478,114	24.38	1,108,156	21.79	2,075	17.56	2,282	20.93

**Waste disposal**	N= 22,468,769		N= 5,085,670		N= 22,468,790		N= 5,085,649		N= 11,815		N= 10,905	
Public collection	18,252,332	81.23	4,005,043	78.75	18,252,353	81.23	4,005,022	78.75	10,942	92.61	9,042	82.92
Not collected	4,216,437	18.77	1,080,627	21.25	4,216,437	18.77	1,080,627	21.25	873	7.39	1,863	17.08
**Sewage system**	N= 21,915,720		N= 5,053,849		N= 21,915,740		N= 5,053,829		N= 11,815		N= 10,687	
Sewage network	11,355,407	51.81	2,718,495	53.79	11,355,422	51.81	2,718,480	53.79	7,002	59.26	5,690	53.24
Septic tank	3,212,555	14.66	729,600	14.44	3,212,556	14.66	729,600	14.44	1,656	14.02	1,565	14.64
Others	7,347,758	33.53	1,605,752	31.77	7,347,762	33.53	1,605,748	31.77	3,157	26.72	3,432	32.11
**ADJUSTMENT VARIABLES**												
**Gender**	N= 22,468,882		N= 5,849,649		N= 22,468,903		N= 5,849,628		N= 11,815		N= 11,570	
Female	11,807,067	52.55	3,112,982	53.22	11,807,076	52.55	3,112,973	53.22	6,126	51.85	4,669	40.35
Male	10,661,815	47.45	2,736,667	46.78	10,661,827	47.45	2,736,655	46.78	5,689	48.15	6,901	59.65
**Age**	N= 22,468,882		N= 5,847,745		N= 22,468,903		N= 5,847,724		N= 11,815		N= 11,569	
Adolescents (13-17 years old)	2,486,277	11.07	360,300	6.16	2,486,278	11.07	360,290	6.16	581	4.92	343	2.96
Young (17-23.9 years old)	3,793,636	16.88	1,152,621	19.71	3,793,637	16.88	1,152,620	19.71	1,865	15.79	1,849	15.98
24-34.9 years old	6,274,728	27.93	2,053,344	35.11	6,274,734	27.93	2,053,338	35.11	4,725	39.99	4,607	39.82
35-44.9 years	3,552,751	15.81	988,300	16.90	3,552,758	15.81	988,320	16.90	2,657	22.49	2,758	23.84
45-54.9 years	2,444,388	10.88	588,100	10.06	2,444,394	10.88	588,130	10.06	1,310	11.09	1,355	11.71
55-64.9 years	2,019,831	8.99	403,200	6.89	2,019,831	8.99	403,150	6.89	529	4.48	512	4.43
Elderly (65 years or older)	1,897,271	8.44	301,900	5.16	1,897,271	8.44	301,880	5.16	148	1.25	145	1.25
**Year of admission to the cohort**	N= 22,468,882		N= 5,849,649		N= 22,468,903			N= 5,849,628		N= 11,815		N= 11,570
2007	4,846,874	21.57	1,139,381	19.48	4,846,877	21.57	1,139,378	19.48	3,957	33.49	3,630	31.37
2008	2,050,336	9.13	630,800	10.78	2,050,338	9.13	630,750	10.78	1,957	16.56	1,831	15.83
2009	1,919,481	8.54	631	10.79	1,919,481	8.54	630,980	10.79	1,559	13.20	1,426	12.32
2010	1,378,131	6.13	1,143,678	19.55	1,378,134	6.13	1,143,675	19.55	1,089	9.22	1,629	14.08
2011	1,697,892	7.56	721,400	12.33	1,697,894	7.56	721,400	12.33	881	7.46	957	8.27
2012	3,638,919	16.20	253,600	4.34	3,638,923	16.20	253,610	4.34	1,364	11.54	914	7.90
2013	2,138,216	9.52	1,017,583	17.40	2,138,216	9.52	1,017,583	17.40	480	4.06	791	6.84
2014	2,845,103	12.66	206,500	3.53	2,845,109	12.66	206,540	3.53	444	3.76	317	2.74
2015	1,953,930	8.70	105,700	1.81	1,953,931	8.70	105,720	1.81	84	0.71	75	0.65

5. Crude Analyses: Incidence, mortality, and case fatality rates from AIDS by SDH strata

Among the 28.3 million Brazilians studied in the period 2007-2015, AIDS incidence was 20.22; 95% CI: 19.98-20.46) per 100,000 Person-years, mortality 6.90; 95% CI: 6.76-7.04) per 100,000 Person-years, and case-fatality rate 6.90; 95% CI: 6.69- 7.12) per 100 Person-years (Table S11).

Higher incidence rates were observed in the south region (40.07), with more than twice as many occurrences compared to the north region (RR: 2.37; 95% CI: 2.26-2.48). The urban area of residence showed an incidence almost 3 times higher than the rural area (RR: 2.91; 95% CI: 2.80-3.04). For mortality, higher rates were also observed in the southern region (RR: 2.51; 95% CI: 2.31-2.73) and urban area (RR: 3.15; 95% CI: 2.93-3.38). For the case-fatality rate the opposite was observed, the south region and the urban area of residence had the lowest rates (RR: 0.95; 95% CI: 0.84-1.08), RR: 0.95; 95% CI: 0.85-1.05), respectively), despite the absence of statistical significance (Table S11).

For the data of socioeconomic status statistical differences were observed in the crude analysis for race skin color with black race showing higher incidence (RR: 1.56; 95% CI: 1.50-1.63), for wealth levels, with the lower levels (levels 3 to 5) showing higher incidences (RR: 1.09; 95% CI: 1.03 - 1.16; RR: 1.14; 95% CI: 1.08-1.20; RR: 1.07; 95% CI: 1.02-1.12, respectively) when compared to the more wealth level (level 1). For mortality, a statistical association was observed, with higher rates among those illiterate/never went to school (RR: 1.88; 95% CI: 1.47-2.39), elementary school (RR: 1. 98; 95% CI: 1.56-2.51), black race (RR: 1.79; 95% CI: 1.68-1.91), the four lower levels of wealth (RR:1.17; 95% CI: 1.05-1.29; RR: 1.25; 95% CI: 1.12-1.39; RR: 1.36; 95% CI: 1.24-1.50; RR: 1.29; 95% CI: 1.18-1.42, respectively). For case-fatality rate, there were higher rates with statistical associations for those illiterate/never went to school (RR: 2.58; 95% CI: 1.75-3.81), elementary school (RR: 1.85; 95% CI: 1.27-2.70), black race (RR: 1.21; 95% CI: 1.09-1.34) and those at wealth level 2 (RR: 1.26; 95% CI: 1.06-1.49) (Table S11).

For those who had received *Bolsa Família* for longer, a protective effect ware observed for all three AIDS outcomes. Lower AIDS incidence was observed for those who had received *Bolsa Família* for more than 10 years (RR: 0.78; 95% CI: 0.75-0.82). Lower AIDS mortality ware observed for those receiving *Bolsa Família* between 5 and 10 years or those with more than 10 years (RR: 0.90; 95% CI: 0.85-0.95; RR: 0.59; 95% CI: 0.54-0.64, respectively). As well, a lower case-fatality rate was observed for those receiving *Bolsa Família* between 5 and 10 years or those with more than 10 years (RR: 0.66; 95% CI: 0.60-0.72; RR: 0.56; 95% CI: 0.50-0.64, respectively). (Table S11).

About household conditions (Block 3), higher AIDS incidence rates were observed in the groups residing in households with wood materials (RR: 1.24; 95% CI: 1.20-1.29), without electrical power (RR: 1.23; 95% CI: 1.19-1.27), with higher household density (more than 2) (RR: 1.23; 95% CI: 1.17-1.29). Also higher AIDS mortality rates were observed in these groups, i.e., those who resided in households with wood material (RR: 1.23; 95% CI: 1.16-1.30), without electrical power (RR: 1.32; 95% CI: 1.25-1.39), with higher household density (more than 2) (RR: 1.27; 95% CI: 1.17-1.37). For AIDS case-fatality rate statistical significance and higher rates were observed for those without electrical power (RR: 1.13; 95% CI: 1.04-1.22) and with others sanitary sewage system (RR: 1.10; 95% CI: 1.02-1.18) (Table S11).

Other characteristics of the household considered precarious presented themselves as protective factors for the incidence and mortality outcomes, probably because they were more common in the rural area of residence, namely: a) lower AIDS incidence rates were observed for those who resided in households with mud and other household materials (RR: 0. 69; 95% CI: 0.66-0.72), water supply not public (RR: 0.63; 95% CI: 0.62-0.66), no waste disposal (RR: 0.45; 95% CI: 0.43-0.47), septic tank sanitary sewage (RR: 0.76; 95% CI: 0.74-0.79) or others (RR: 0.64; 95% CI: 0.62-0.66); b) as well as lower mortality rates were observed in the same groups for those who resided in households with mud and other household materials (RR: 0.64; 95% CI: 0.59-0. 69), with water supply that was not public (RR: 0.65; 95% CI: 0.61-0.68), without waste disposal (RR: 0.44; 95% CI: 0.41-0.47), with septic tank sanitary sewage (RR: 0.67; 95% CI: 0.63-0.72) or others (RR: 0.59; 95% CI: 0.56-0.62) (Table S11).

Regarding health and behavioral aspects (block 4), almost three times the AIDS case-fatality rate were observed for those PLWA who did not start treatment (RR: 2.74; 95% CI: 2.56-2.93) and for People who inject drugs (RR: 2.96; 95% CI: 2.45-3.57) (Table S11).

Table S11. Incidence, mortality, and case fatality rates from AIDS in Brazilians aged 13 years or older (N = 28,318,532) recorded in CadÚnico from 2007 to 2015, stratified by geographic, socioeconomic, household characteristics and health and behavioral aspects (crude analyses).

	**AIDS cases in cohort**			**AIDS death in cohort**			**AIDS death in PLWA cohort**		
	**n**	**Incidence /100,000 (95% CI)**	**RR (95% CI)**	**n**	**Mortality/100,000 (95% CI)**	**RR (95% CI)**	**n**	**Case-fatality rate/100 (95% CI)**	**RR (95% CI)**
**Person-years at risk**	137986981			138068520			58,517.92		
**Cohort**	27,900	20.22 (19.98-20.46)		9,528	6.90 (6.76-7.04)		4,040	6.90 (6.69-7.12)	
**Follow-up time**									
Mean (sd)	4.87 (2.75)			4.88 (2.75)			2.62 (2.15)		
Median (IQR)	4.59 (2.49-7.51)			4.60 (2.49-7.52)			2.21 (0.75-4.02)		
**BLOCK 1 – GEOGRAPHIC FACTORS**									
**Region of residence**	N=28,316,715			N=28,316,748			N=22,334		
North	2533	16.93 (16.28-17.60)	1.00	751	5.02 (4.67-5.39)	1.00	339	6.57 (5.90-7.30)	1.00
Northeast	7992	15.66 (15.32-16.00)	0.92 (0.88-0.97)	2372	4.64 (4.46-4.84)	0.93 (0.85-1.01)	1193	6.71 (6.34-7.11)	1.02 (0.91-1.15)
Southeast	9480	20.27 (19.86-20.68)	1.20 (1.15-1.25)	3937	8.41 (8.15-8.68)	1.68 (1.55-1.81)	1383	7.46 (7.07-7.86)	1.14 (1.01-1.28)
South	5958	40.07 (39.06-41.10)	2.37 (2.26-2.48)	1875	12.59 (12.04-13.18)	2.51 (2.31-2.73)	819	6.27 (5.85-6.71)	0.95 (0.84-1.08)
Mid-West	1933	18.73 (17.92-19.59)	1.11 (1.04-1.17)	593	5.74 (5.30-6.23)	1.15 (1.03-1.27)	306	7.71 (6.89-8.62)	1.17 (1.01-1.37)
**Area of residence**	N=28,226,360			N=28,316,748			N=22,084		
Rural	2497	8.08 (7.77–8.40)	1.00	798	2.58 (20.41-2.77)	1	387	7.25 (6.56-8.01)	
Urban	25103	23.57 (23.28-23.86)	2.91 (2.80-3.04)	8657	8.12 (7.95-8.30)	3.15 (2.93-3.38)	3626	6.89 (6.66-7.11)	0.95 (0.85-1.05)
**BLOCK 2 – SOCIOECONOMIC STATUS**									
**Education**	N=25,790,719			N=25,790,750			N=19,430		
Illiterate/Never went to school	2329	17.68 (16.98-18.42)	0.84 (0.76-0.94)	1011	7.67 (7.21-8.16)	1.88 (1.47-2.39)	448	10.18 (9.28-11.17)	2.58 (1.75-3.81)
Literate/pre-school	161	14.13 (12.11-16.49)	0.67 (0.56-0.81)	48	4.21 (31.17-5.59)	1.03 (0.71-1.49)	19	6.11 (3.90-9.58)	1.55 (0.86-2.79)
Elementary school	17186	22.18 (21.85-22.51)	1.06 (0.95-1.18)	6291	8.11 (7.91-8.32)	1.98 (1.56-2.51)	2604	7.30 (7.02-7.58)	1.85 (1.27-2.70)
High school	4413	16.86 (16.37-17.36)	0.81 (0.72-0.90)	1096	4.18 (3.94-4.44)	1.23 (0.80-1.30)	471	5.48 (5.01-6.00)	1.39 (0.94-2.05)
Higher education	353	20.94 (18.87-23.24)	1.00	69	4.09 (3.23-5.18)	1.00	27	3.94 (2.70-5.75)	1
**Race/skin color**	N=26,703,594			N=26,703,624			N=21,105		
White	8587	20.84 (20.41-21.29)	1.00	2945	7.15 (6.895-7.41)	1.00	-		-
Yellow/Asian	82	17.14 (13.80-21.28)	0.82 (0.66-1.02)	27	5.64 (3.87-8.23)	0.79 (0.54-1.15)	-		-
White/Asian	8663	20.80 (20.37-21.24)	1.00	2972	7.13 (6.88-7.39)	1.00	1200	6.45 (6.10-6.83)	1
Pardo	14215	18.32 (18.02-18.62)	0.88 (0.86-0.90)	4721	6.08 (5.61-6.26)	0.85 (0.81-0-89)	2092	7.01 (6.71-7.31)	1.09 (1.01-1.16)
Black	3429	32.51 (31.44-33.61)	1.56 (1.50-1.63)	1347	12.76 (12.09-13.46)	1.79 (1.68-1.91)	534	7.80 (7.17-8.49)	1.21 (1.09-1.34)
Indigenous	103	13.05 (10.76-15.83)	0.63 (0.52-0.76)	33	4.18 (2.97-5.88)	0.59 (0.42-0.83)	11	4.69 (2.60-8.47)	0.7390.40-1.32)
**Wealth levels**	N=28,310,890			N=28,310,923			N=22,331		
Level 1 (Higher wealth)	1731	18.77 (17.91-19.68)	1.00	501	5.43 (4.98-5.93)	1.00	184	7.16 (6.19-8.27)	1
Level 2	3704	19.12 (18.52-19.75)	1.02 (0.96-1.08)	1227	6.33 (5.99-6.70)	1.17 (1.05-1.29)	506	9.01 (8.26-9.83)	1.26 (1.06-1.49)
Level 3	2858	20.47 (19.74-21.24)	1.09 (1.03-1.16)	947	6.78 (6.36-7.23)	1.25 (1.12-1.39)	383	8.18 (7.40-9.04)	1.14 (0.96-1.36)
Level 4	7793	21.33 (20.87-21.81)	1.14 (1.08-1.20)	2709	7.41 (7.14-7.70)	1.36 (1.24-1.50)	1150	6.55 (6.18-6.94)	0.91 (0.78-1.07)
Level 5 (Lower wealth)	11807	20.05 (19.70-20.42)	1.07 (1.02-1.12)	4142	7.03 (6.82-7.25)	1.29 (1.18-1.42)	1815	6.47 (6.18-6.77)	0.90 (0.78-1.05)
**Receipt time of *Bolsa Família***	N = 28,318,190			N=28,318,223			N=22,338		
Does not receive	6176	18.19 (17.75-18.65)	1.00	2227	6.56 (6.29-6.84)	1.00	856	8.24 (7.71-8.82)	1.00

Less than 2 years	4168	25.45 (24.69-26.23)	1.40 (1.34-1.45)	1678	10.24 (9.76-0.74)	1.56 (1.47-1.66)	683	9.54 (8.85-10.28)	1.15 (1.05-1.28)
Between 2 and 5 years	6342	24.76 (24.16-25.38)	1.36 (1.31-1.41)	2373	9.26 (8.89-9.64)	1.41 (1.33-1.50)	1000	8.18 (7.68-8.70)	0.99 (0.90-1.09)
Between 5 and 10 years	8323	19.96 (19.54-20.40)	1.10 (1.06-1.13)	2462	5.90 (5.67-6.14)	0.90 (0.85-0.95)	1136	5.44 (5.13-5.77)	0.66 (0.60-0.72)
More than 10 years	2891	14.20 (13.69-14.72)	0.78 (0.75-0.82)	788	3.87 (3.61-4.15)	0.59 (0.54-0.64)	365	4.64 (4.19-5.14)	0.56 (0.50-0.64)
**BLOCK 3 – HOUSEHOLD CONDITIONS**									
**Household material**	N== 27,553,988			N=27,554,016			N=21,704		
Brick	21226	20.11 (19.84-20.38)	1.00	7317	6.93 (6.77-7.09)	1.00	3054	6.87 (6.63-7.12)	1
Wood	3844	25.02 (24.24-25.82)	1.24 (1.20-1.29)	1309	8.51 (8.06-8.99)	1.23 (1.16-1.30)	597	7.10 (6.55-7.69)	1.03 (0.95-1.13)
Mud and others	2033	13.87 (13.28-14.49)	0.69 (0.66-0.72)	652	4.45 (4.12-4.80)	0.64 (0.59-0.69)	303	6.56 (5.86-7.34)	0.95 (0.85-1.07)
**Electrical power**	N= 27,554,428			N=27,554,456			N=21,701		
Yes	22899	19.42 (19.17-19.67)	1.00	7748	6.57 (6.42-6.71)	1.00	3270	6.74 (6.52-6.98)	1.00
No	4201	23.82 (23.11-24.55)	1.23 (1.19-1.27)	1528	8.66 (8.23-9.10)	1.32 (1.25-1,39)	682	7.59 (7.04-8.19)	1.13 (1.04-1.22)
**Household density** (residents/room)	N== 25,864,364			N=25,864,390			N=20,271		
up to 2	23511	19.68 (19.43-19.94)	1.00	8045	6.73 (6.59-6.88)	1.00	3409	6.83 (6.10-7.07)	1.00
more than 2	1807	24.15 (23.06-25.29)	1.23 (1.17-1.29)	639	8.53 (7.90-9.22)	1.27 (1.17-1.37)	297	7.14 (6.38-8.01)	1.04 (0.93-1.18)
**Water supply**	N= 27,554,446			N=27,554,474			N=21,701		
Public network	21922	22.18 (21.89-22.47)	1.00	7476	7.56 (7.39-7.73)	1.00	3149	6.80 (6.56-7.04)	1.00
Others	5177	14.10 (13.72-14.49)	0.63 (0.62-0.66)	1799	4.90 (4.68-5.13)	0.65 (0.61-0.68)	803	7.22 (6.73-7.73)	1.06 (0.98-1.15)
**Waste disposal**	N= 27,554,109			N=27,554,137			N=21,702		
Public collection	23870	22.90 (22.61-23.19)	1.00	8191	7.85 (7.68-8.02)	1.00	3463	6.85 (6.62-7.08)	1.00
Not collected	3230	10.31 (9.96-10.67)	0.45 (0.43-0.47)	1085	3.46 (3.26-3.67)	0.44 (0.41-0.47)	490	7.12 (6.51-7.77)	1.04 (0.94-1.14)
**Sewage system**	N= 26,969,245			N=26,969,272			N=21,493		
Sewage network	15474	24.08 (23.70-24.46)	1.00	5506	8.56 (8.34-8.79)	1.00	2181	6.66 (6.39-6.95)	1.00
Septic tank	3723	18.39 (17.81-18.99)	0.76 (0.74-0.79)	1168	5.77 (5.44-6.11)	0.67 (0.63-0.72)	539	6.65 (6.11-6.95)	1.00 (0.91-1.10)
Others	7633	15.43 (15.09-15.78)	0.64 (0.62-0.66)	2504	5.06 (4.87-5.26)	0.59 (0.56-0,62)	1195	7.32 (6.92-7.75)	1.10 (1.02-1.18)
**BLOCK 4 – HEALTH AND BEHAVIORAL ASPECTS**									
**Treatment**							N=22,338		
Yes	-	-	-	-	-	-	2855	5.62 (5.42-5.83)	1.00
No	-	-	-	-	-	-	1185	15.39 (14.54-16.29)	2.74 (2.56-2.93)
**Exposure Category**							N=18,823		
Homosexual	-	-	-	-	-	-	224	3.91 (3.43-4.45)	1.00
Bisexual	-	-	-	-	-	-	107	4.60 (3.81-5.56)	1.18 (0.94-1.48)
Heterosexual	-	-	-	-	-	-	2414	5.79 (5.56-6.02)	1.48 (1.29-1.70)
People who inject drugs	-	-	-	-	-	-	210	11.56 (10.10-13.23)	2.96 (2.45-3.57)
**Means of transmission**							N=18,849		
Blood transfusion							4	7.725 (2.90-20.58)	1.00
Accident with biological material							0	-	-
Sexual, with person of the opposite sex							2,413	5.78 (5.56-6.02)	0.75 (0.28-2.00)
Sexual, with person of the same sex or both							332	4.12 (3.70-4.59)	0.53 (0.20-1.43)
Injecting drugs							211	11.54 (10.08-13.20)	1.49 (0.55-4.02)
**ADJUSTMENT VARIABLES**									

**Gender**	N=28,318,190			N=28,318,223			N=22,338		
Female	12825	18.03 (17.72-18.35)	1.00	4134	5.81 (5.63-5.99)	1.00	1672	5.72 (5.45-6.00)	1.00
Male	15075	22.55 (22.19-22.91)	1.25 (1.22-1.28)	5394	8.06 (7.85-8.28)	1.39 (1.33-1.44)	2368	8.09 (7.77-8.42)	1.41 (1.33-1.51)
**Age**	N=28,316,286			N=28,316,319			N=22,337		
Adolescents (13-17 years old)	995	6.09 (5.72-6.48)	1.00	172	1.05 (0.91-1.22)	1.00	89	4.75 (3.86-5.85)	1.00
Young (17-23.9 years old)	4073	14.79 (14.34-15.25)	2.43 (2.27-2.60)	848	3.08 (2.88-3.29)	2.92 (2.48-3.44	408	4.07 (3.69-4.48)	0.86 (0.68-1.08)
24-34.9 years old	10816	25.94 (25.45-26.43)	4.26 (3.99-4.55)	3360	8.05 (7.78-8.33)	7.65 (6.56-8.92	1539	6.21 (5.91-6.58)	1.31 (1.06-1.62)
35-44.9 years	6813	31.77 (21.02-32.53)	5.22 (4.88-5.58)	2781	12.96 (12.48-13.45)	12.31 (10.55-14.36)	1070	7.89 (7.43-8.38)	1.66 (1.34-2.06)
45-54.9 years	3451	25.78 (24.93-26.65)	4.23 (3.95-4.54)	1511	11.28 (10.72-11.86)	10.72 (9.15-12.55	574	9.91 (9.13-10.75)	2.08 (1.67-2.61)
55-64.9 years	1345	14.00 (13.27-14-77)	2.30 (2.12-2.50)	642	6.68 (6.18-7.22)	6.35 (5.36-7.51	278	14.14 (12.57-15.90)	2.98 (2.34-3.78)
Elderly (65 years or older)	406	5.10 (4.63-5.62)	0.84 (0.75-0.94)	214	2.69 (2.35-3.07)	2.56 (2.09-3.12	82	15.94 (12.84-19.79)	3.36 (2.49-4.53)
**Year of admission to the cohort**	N=28,318,190			N=28,318,223			N=22,338		
2007	8973	17.59 (17.23-17.96)	1.00	3076	6.03 (5.82-6.24)	1.00	1389	5.76 (5.47-6.07)	1.00
2008	4581	23.24 (22.57-23.92)	1.32 (1.27-1.37)	1687	8.55 (8.15-8.97)	1.42 (1.34-1.51	732	6.59 (6.12-7.08)	1.14 (1.04-1.25)
2009	3570	22.12 (21.40-22.85)	1.26 (1.21-1.31)	1229	7.61 (7.19-8.05)	1.26 (1.18-1.35	528	6.82 (6.26-7.43)	1.18 (1.07-1.31)
2010	3193	23.32 (22.53-24.15)	1.36 (1.27-1.38)	1044	7.62 (7.17-8.10)	1.26 (1.18-1.36	438	6.93 (6.31-7.61)	1.20 (1.08-1.34)
2011	2201	20.96 (20.11-21.86)	1.19 (1.14-1.25)	730	6.95 (6.46-7.47)	1.15 (1.06-1.25	292	8.07 (7.19-9.05)	1.40 (1.23-1.59)
2012	2761	20.43 (19.68-21.21)	1.16 (1.11-1.21)	933	6.90 (6.47-7.36)	1.14 (1.06-1.23	359	10.01 (9.02-11.10)	1.74 (1.55-1.95)
2013	1514	19.60 (18.63-20.61)	1.11 (1.05-1.18)	470	6.08 (5.56-6.66)	1.01 (0.92-1.11	166	11.58 (9.95-13.48)	2.01 (1.71-2.36)
2014	897	19.68 (18.43-21.01)	1.12 (1.04-1.20)	282	6.19 (5.50-6.95)	1.03 (0.91-1.16	116	21.82 (18.19-26.17)	3.79 (3.13-4.58)
2015	210	18.38 (16.05-21.04)	1.04 (0.91-1.20)	77	6.74 (5.39-8.42)	1.12 (0.89-1.40	20	36.10 (23.29-55.96)	6.26 (4.03-9.74)
**Municipal HIV/AIDS endemicity and surveillance in the period (2007-2015)**									
Average AIDS morbidity rate	N= 28,313,238		1.03 (1.03-1.04)		-	-		-	-
Average AIDS mortality rate		-	-	N = 28,313,271		1.12 (1.11-1.12)		-	-
Average AIDS case-fatality rate		-	-		-	-	N= 18,823		1.02 (1.01-1.02)

Notes:“-” variable not included in the model

Abbreviations: PLWA: People Living with AIDS; CI: confidence interval; RR: Rate Ratio; sd: standard deviation; IQR: Interquartile Range.

6. Trend of AIDS morbidity and mortality indicators

Official data from the Brazilian Ministry of Health[13] show AIDS morbidity rates higher than those found here (Figure S2) and with a decreasing trend (21.7/100,000 inhabitants in 2008 - 20.1/100,000 inhabitants in 2015). Two possible factors support these observed differences: (a) the population of the studied cohort is composed of the portion of poorer Brazilians and, therefore, with greater lack of information and access to health services, which may be reflected in a lower detection of the disease (late diagnosis and underreporting)[14]; and (b) The data shown are from two of the information systems (SINAN and SIM, as per their Portuguese acronyms) that hold HIV/AIDS surveillance in Brazil, while two other important systems, the Laboratory Test Control System (SISCEL, as per its Portuguese acronym) and the Logistic Control of Medicines (SICLOM, as per its Portuguese acronym) capture an expressive amount of AIDS cases that are outside the SINAN scope.[13]

Despite the growth trend, in the last year of follow-up, it was observed a drop-in AIDS morbidity rate (Figure S2), also observed in another national study, which was attributed to the implementation of the Test and Treat Initiative in Brazil in the year 2014.[15]

As for the AIDS mortality rates, it is observed a national stationary trend in the official data[13] (6.2/100,000 inhabitants in 2008 and in 2015), but with rates similar to those found by means of this investigation (Figure S2), which reflects a better quality and universality of the SIM system in AIDS death surveillance in Brazil.

Official data from Brazil are not available for the AIDS case-fatality rates; however, it is assumed that its decreasing trend (Figure S2) is linked to universal access to the ART, since 1996, and to the evolution of eligibility criteria for its beginning. Firstly, treatment was indicated for those individuals diagnosed with AIDS and with CD4 cell counts ≥500. In 2014, the Test and Treat Initiative was implemented,[16] which contributed greatly to reducing mortality and transmission of the disease.[15]

Figure S2. Trend of AIDS morbidity and mortality indicators of Brazilians aged 13 years and older registered in CadÚnico in the period from 2007 to 2015.

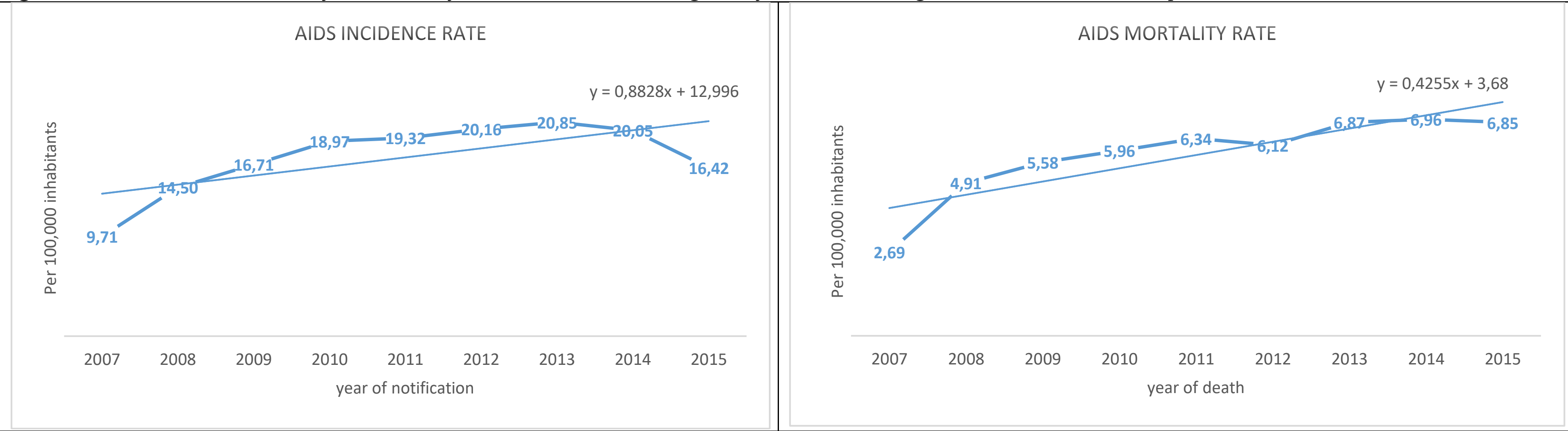

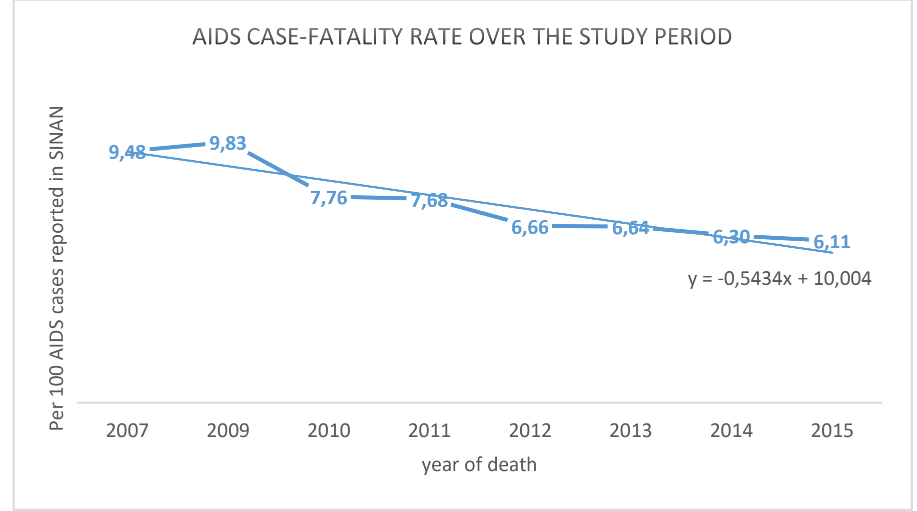

$$\textit{AIDS incidence rate} = \frac{\textit{reported cases of AIDS per year}}{\textit{number of individuals in the cohort per year}\,*}\, x\ 100.000$$

$$\textit{AIDS mortality rate} = \frac{\textit{registered AIDS deaths per year}}{\textit{number of individuals in the cohort per year}\,*}\, x\ 100.000$$

$$\textit{AIDS case} - \textit{fatality rate over the study period} = \frac{\textit{registered AIDS deaths per year}}{\textit{number of individuals living with AIDS in the cohort per year}\,**}\, x\ 100$$

*number of individuals n in the cohort per year=n in the year of entering the cohort + n in the previous year - n of deaths in the previous year
**n living with AIDS in the cohort per year= n of notified cases in the year + n of cases in the previous year- n of deaths in the previous year

REFERENCES

1. Barreto M, Ichihara MY, Pescarini JM, et al. Cohort Profile: The 100 Million Brazilian Cohort. Int J Epidemiol. 2022;51 (2):e27-e38.
2. Sanni Ali M, Ichihara MY, Lopes LC, et al. Administrative data linkage in Brazil: Potentials for health technology assessment. Front Pharmacol 2019;10 (SEP):1–20.
3. Pita R, Pinto C, Sena S, et al. On the Accuracy and Scalability of Probabilistic Data Linkage over the Brazilian 114 Million Cohort. IEEE J Biomed Heal Informatics 2018;22 (2):346–53.
4. Pinto C, Pita R, Barbosa G, et al. Probabilistic Integration of Large Brazilian Socioeconomic and Clinical Databases. Proc - IEEE Symp Comput Med Syst 2017;2017–June:515–20.
5. Barreto M, Alves A, Sena S, Fiaccone R, Amorim L, Ichihara MY, Barreto M. Assessing the accuracy of probabilistic record linkage of social and health databases in the 100 million Brazilian cohort. Int J Popul Data Sci 2017; (0):23889.
6. Pita R, Pinto C, Barreto M, et al. Design and evaluation of probabilistic record linkage methods supporting the Brazilian 100-million cohort initiative. Int J Popul Data Sci 2017;1 (1):23889.
7. Brazil. Brazilian Ministry of Health. Secretariat of Health Surveillance. Programa Nacional de DST e Aids. Critérios de definição de casos de aids em adultos e crianças. [National STD and AIDS Program. Criteria for defining AIDS cases in adults and children.] Brasília: Ministry of Health, 2003. *https://bvsms.saude.gov.br/bvs/publicacoes/criterios_definicao_AIDS_adultos_criancas.pdf.* Accessed December 10, 2020.
8. World Health Organization. International statistical classification of diseases and related health problems, 10th revision, Fifth edition, 2016. *https://apps.who.int/iris/handle/10665/246208.* Accessed January 20, 2021.
9. Norton EC, Miller MM, Kleinman LC. Computing adjusted risk ratios and risk differences in Stata. *The Stata Journal.* 2013;13 (3):492–509.
10. Andrade CLT, Szwarcwald CL. Socio-spatial inequalities in the adequacy of Ministry of Health data on births and deaths at the municipal level in Brazil, 2000-2002. Cad. Saúde Pública, 2007; 23 (5):1207-1216.
11. Brazil. Brazilian Ministry of Health: Minister's Office. Ordinance No. 1,271, June 6, 2014. *https://bvsms.saude.gov.br/bvs/saudelegis/gm/2014/prt1271_06_06_2014.html*
12. Brazil. Brazilian Ministry of Health: Department of STD, AIDS and Viral Hepatitis. Protocolo Clínico e Diretrizes Terapêuticas para Manejo da Infecção pelo HIV em Adultos. [Clinical Protocol and Therapeutic Guidelines for Management of HIV Infection in Adults]. Brasília: Ministry of Health, 2013. *https://bvsms.saude.gov.br/bvs/publicacoes/protocolo_clinico_manejo_hiv_adultos.pdf*
13. Brazil. Brazilian Ministry of Health: Department of Chronic Conditions Diseases and Sexually Transmitted Infections - DCCI. Indicadores e Dados Básicos do HIV/AIDS nos Municípios Brasileiros. [Indicators and Basic Data on HIV/AIDS in Brazilian Municipalities]. *http://indicadores.aids.gov.br/*. Accessed May 25, 2021.
14. Vincent W, Sevelius J, Lippman SA, Linnemayr S, Arnold EA. Identifying Opportunities for Collaboration Across the Social Sciences to Reach the 10-10-10: A Multilevel Approach. *J Acquir Immune Defic Syndr.* 2019;82 Suppl 2 (2):S118-S123.
15. Pereira G, Sabidó M, Caruso A, Benzaken AS. Decline in reported AIDS cases in Brazil after implementation of the test and treat initiative. *BMC infectious diseases.* 2019;19 (1):579-586.
16. Brazil. Brazilian Ministry of Health. Secretariat of Health Surveillance: : Department of STD, AIDS and Viral Hepatitis. Protocolo clínico e diretrizes terapêuticas para o manejo da infecção pelo HIV em adultos. [Clinical Protocol and Therapeutic Guidelines for Management of HIV Infection in Adults]. Brasília: Ministry of Health, 2018. *http://www.aids.gov.br/pt-br/pub/2013/protocolo-clinico-e-diretrizes-terapeuticas-para-manejo-da-infeccao-pelo-hiv-em-adultos*. Accessed May 25, 2021.
